# Principles and Overview of Sampling Methods for Modeling Macromolecular Structure and Dynamics

**DOI:** 10.1371/journal.pcbi.1004619

**Published:** 2016-04-28

**Authors:** Tatiana Maximova, Ryan Moffatt, Buyong Ma, Ruth Nussinov, Amarda Shehu

**Affiliations:** 1 Department of Computer Science, George Mason University, Fairfax, Virginia, United States of America; 2 Basic Science Program, Leidos Biomedical Research, Inc. Cancer and Inflammation Program, National Cancer Institute, Frederick, Maryland, United States of America; 3 Sackler Institute of Molecular Medicine, Department of Human Genetics and Molecular Medicine, Sackler School of Medicine, Tel Aviv University, Tel Aviv, Israel; 4 Department of Biongineering, George Mason University, Fairfax, Virginia, United States of America; 5 School of Systems Biology, George Mason University, Manassas, Virginia, United States of America; Max Planck Institute for Biophysical Chemistry, GERMANY

## Abstract

Investigation of macromolecular structure and dynamics is fundamental to understanding how macromolecules carry out their functions in the cell. Significant advances have been made toward this end in silico, with a growing number of computational methods proposed yearly to study and simulate various aspects of macromolecular structure and dynamics. This review aims to provide an overview of recent advances, focusing primarily on methods proposed for exploring the structure space of macromolecules in isolation and in assemblies for the purpose of characterizing equilibrium structure and dynamics. In addition to surveying recent applications that showcase current capabilities of computational methods, this review highlights state-of-the-art algorithmic techniques proposed to overcome challenges posed in silico by the disparate spatial and time scales accessed by dynamic macromolecules. This review is not meant to be exhaustive, as such an endeavor is impossible, but rather aims to balance breadth and depth of strategies for modeling macromolecular structure and dynamics for a broad audience of novices and experts.

## Author Summary

This paper provides an overview of recent advancements in computational methods for modeling macromolecular structure and dynamics. The focus is on methods aimed at providing efficient representations of macromolecular structure spaces for the purpose of characterizing equilibrium dynamics. The overview is meant to provide a summary of state-of-the-art capabilities of these methods from an application point of view, as well as highlight important algorithmic contributions responsible for recent advances in macromolecular structure and dynamics modeling.

## Introduction

A detailed understanding of how fundamental biological macromolecules, such as proteins and nucleic acids, carry out their biological functions is central to obtaining a detailed and complete picture of molecular mechanisms in the healthy and diseased cell. Furthering our understanding of macromolecules is central to understanding our own biology, as proteins and nucleic acids are central components of cellular organization and function. Many abnormalities involve macromolecules incapable of performing their biological function [1–4], either due to external perturbations, such as environmental changes, or internal perturbations, such as mutations [5–10], affecting their ability to assume specific function-carrying structures.

It has long been known that the ability of a macromolecule to carry out its biological function is dependent on its ability to assume a specific three-dimensional structure (in other words, structure carries function) [11,12]. However, an increasing number of experimental, theoretical, and computational studies have demonstrated that function is the result of a complex yet precise relationship between macromolecular structure and dynamics [13–21]. Most notably, in proteins, the ability to access and switch between different structural states is key to biomolecular recognition and function modulation [22,23].

The intrinsic dynamic personality of macromolecules [18] is not surprising and can indeed be derived from first principles. Feynman highlighted the jiggling and wiggling of atoms well before wet-laboratory techniques provided evidence of macromolecular dynamics [24]. In the late 1970s and early 1980s, it became clear that treating macromolecules as thermodynamic systems and employing basic principles allowed anticipating and simulating their intrinsic state of perpetual motion [25,26]. The thermodynamic uncertainty principle was coined by Cooper in [26] to refer to the inherent uncertainty about the particular state a macromolecule is or will evolve to at any given time. Cooper was among the first to employ tools from statistical thermodynamics to show that macromolecular fluctuations are a direct result of thermal interaction with the environment and that any detailed description of macromolecular structure and dynamics entailed employing probability distributions. Further work by Wolynes and colleagues continued in this spirit, popularizing a statistical treatment of macromolecules with tools borrowed from statistical mechanics and culminating in the energy landscape view [5,13,27,28].

Great advances have been made in the wet laboratory to elucidate macromolecular structure and dynamics. Nowadays, techniques such as X-ray crystallography, Nuclear Magnetic Resonance (NMR), and cryo-Electron Microscopy (cryo-EM) can resolve equilibrium structures and quantify equilibrium dynamics. Macroscopic measurements obtained in the wet laboratory are Boltzmann-weighted averages over microstates/structures populated by a macromolecule at equilibrium. Though in principle wet-laboratory techniques are limited in their description of equilibrium structures and dynamics to the time scales probed in the wet laboratory (a problem also known as ensemble-averaging), much progress has been made [29–31]. The ensemble of structures contributing to macroscopic measurements obtained in the wet laboratory can be unraveled with complementary computational techniques [32–36]. In addition, wet-laboratory techniques, such as NMR spectroscopy, can on their own directly elucidate picosecond-millisecond long relaxation phenomena [37,38]. Indeed, recent single-molecule techniques have achieved great success at bypassing the ensemble averaging problem and elucidating equilibrium dynamics [31,39–47].

Transitions of a macromolecule between successive structural states can be captured in the wet laboratory [31,46,48–53]. Wet-laboratory techniques can resolve key well-populated intermediate structures along a transition [52,54], but they are generally unable to span all the time scales involved in a transition and so fully account for a macromolecule’s equilibrium dynamics. A complete characterization of macromolecular dynamics remains elusive in the wet laboratory due to the disparate time scales that may be involved. Dwell times at successive states along a reaction may be too short to be detected in the wet laboratory. The actual time a macromolecule spends during a transition event can be short compared to its dwell time in any particular thermodynamically stable or meta-stable structural state. Indeed, neither wet- nor dry-laboratory techniques can, on their own, span all spatial and time scales involved in dynamic macromolecular processes [55].

Macromolecular modeling research in silico is driven by the need to complement wet-laboratory techniques and obtain a comprehensive and detailed characterization of equilibrium dynamics. Such a characterization poses outstanding challenges in silico. In principle, a full account of macromolecular dynamics requires a comprehensive characterization of both the structure space available to a macromolecule at equilibrium as well as the underlying free energy surface that governs accessibility of structures and transitions between structures. Early work on protein modeling focused on short protein chains and simplified representations models that laid out amino-acid chains on lattices. These distinct choices made it possible to perform interesting calculations revealing key properties of protein folding and unfolding [56], as well as predict quantities of importance in protein stability and function, such as pKas of ionizable groups [57]. On-lattice models incidentally also allowed key theoretical findings on the computational complexity associated with computing lowest free-energy states in the context of ab initio (now also known as de novo) protein structure prediction [58–60]. The computational complexity of finding the global minimum energy conformation was shown to be NP-hard. These findings made the case that sophisticated algorithms would be needed to complement wet-laboratory characterizations of macromolecular structure and dynamics for the purpose of elucidating biological function.

The advent of Molecular Dynamics (MD) simulations and the concept of an energy function promised to revolutionize macromolecular modeling, as in principle the entire equilibrium dynamics could be simulated by simply following the motions of the atoms constituting a macromolecule down the slope of the energy function. Research in this direction was made possible by a growing set of equilibrium structures resolved in the wet laboratory, from myoglobin [61,62] and lysozyme [63] by 1967 to more than a hundred thousand structures now freely available for anyone in the Protein Data Bank (PDB) [64]. Seminal work in the Karplus laboratory on the MD method and in the Lifson laboratory on the design of consistent energy functions and simplified molecular models set the stage for a computational revolution in structural biology. Commercialization of computers was critical to this revolution.

MD simulations had been shown successful in reproducing equilibrium properties of argon [65], but it was McCammon and Karplus who provided the earliest demonstration in 1977 of the power of MD-based modeling to simulate protein dynamics [25]: a short 9.2 picosecond-long trajectory was obtained showing in-vacuum, atomistic fluctuations of the bovine pancreatic trypsin inhibitor around its native, folded structure. Realizing the power of MD simulations to extract precious information on macromolecular structure and dynamics, the Karplus laboratory democratized modeling by offering the CHARMM program to the computational community [66]. Further work by Karplus and McCammon showed that significant features of protein dynamics would only emerge over longer time scales. The simulation in [67] reached 100 picoseconds, but it would soon become clear that MD-based probings of macromolecular structure and dynamics were in practice limited by both macromolecular size (spatial scale) and time of a phenomenon under investigation (time scale). A significant body of complementary work in macromolecular structure and modeling investigated non-MD based methods. In fact, two years earlier to the 1977 MD simulation by Karplus of equilibrium fluctuations of the bovine pancreatic trypsin inhibitor, Levitt and Warshel had presented a computer simulation of the folding of the same inhibitor through a simplified (now known as coarse-grained) model, in which each residue was reduced to one pseudo-atom, and an algorithm based on steepest descent [68]. Reproducibility of this work has so far remained elusive.

Further work by Levitt and Warshel, prompted by the visionary Lifson at the Weizmann Institute of Science, focused on the design of a consistent energy function for proteins [69]. The idea was to come up with a small number of consistent parameters that could be transferable from molecule to molecule and not depend on the local environment of an atom. Once such an energy function was implemented, simple algorithms could then be put together by making use of the function, its first derivative (the force vector), and the second derivative (the curvature of the energy surface). It is interesting to note that though Lifson and Warshel were the first to introduce a consistent energy function, they did so for small organic hydrocarbon molecules. It was Levitt who realized that their parameters could be used to carry out calculations on proteins. In 1969, Levitt published the first non-MD, steepest descent algorithm on a simplified model encoding only heavy atoms of the X-ray structures of hemoglobin and lysozyme [70]. This work was seminal for Levitt and Warshel to claim the first simulation of protein folding [68]. The algorithm used in these simulations was quite sophisticated, changing torsion angles, as proposed by Scheraga [71], and using normal modes to rapidly compute low-energy paths out of local minima [72].

Further work on coarse-grained and multiscale models built with the quantum mechanics (QM)/molecular mechanics (MM) method proposed by Warshel [73] was seminal in allowing simulation to reach longer spatial and time scales. Warshel, who had a background in quantum mechanics, realized that large molecular systems could be spatially divided into a region demanding quantum mechanical calculations (e.g., due to bonds being broken) with the rest sufficiently represented by empirical force fields. This method remains the cornerstone of modern multiscale modeling [74–80] and, together with the idea of representing complex systems in different resolutions at different time and length scales [76], has allowed simulations to elucidate structures, dynamics, and the biological activity of systems of increasing complexity, from enzymes [74,77,81] to complex molecular machines [82–91].

In tandem with these developments, a new method, Metropolis Monte Carlo (MC) [92,93], made its debut in computational structural biology. In 1987, important work in the Scheraga laboratory introduced an MC-based minimization method to simulate protein folding [94]. In 1996, the Karplus laboratory demonstrated the ability of MC simulations on a cubic lattice to simulate the folding mechanism of a protein-like heteropolymer of 125 beads [95]. Following work in the Scheraga laboratory further made the case for the utility of MC-based methods in studies of macromolecular structure and dynamics [96–98]. Kinetic MC methods were designed to address the lack of kinetics in the classic MC framework [99]. In light of contributions that gave birth to computational structural biology [100], it is no surprise that the Nobel 2013 prize in chemistry recognized computational scientists, namely, Karplus, Warshel, and Levitt for their seminal work in the development of multiscale models for complex chemical systems [101–103].

Improvements in hardware over the last forty years have been critical to extending the reach of MD- and MC-based modeling. For example, MD-based studies have expanded their scope, scale, and thus applicability due to specialized architectures, such as Anton [104,105], Graphics Processing Units (GPUs) [106–109], and petascale national supercomputers, such as BlueWaters, Titan, Mira, Stampede [110,111]. The pervasiveness of supercomputing has spurred great advances in algorithmic techniques to effectively parallelize MD. Typically, in parallel MD, the interacting particles are spatially divided into subdomains that are assigned to different processors. In this framework, load balancing becomes an issue for large-scale MD simulations now performed on thousands of processors and involving billions of particles [112]. Many techniques now exist for dynamic load balancing [113]. In addition, while each processor is responsible for advancing its own particles in time, processors need to exchange information; accurate force calculations require knowledge of neighbor particle positions. Work in [114] describes recent strategies for efficient neighbor searches in parallel MD. Other techniques that permit parallelization of MD address and optimize force splitting in the context of the particle-mesh Ewald algorithm [115]. It is worth noting that many of these techniques are now integrated in publicly-available parallel MD code, such as NAMD [116].

Important contributions in enhancing exploration capability have also been made from non-MD or non-MC frameworks but rather adaptations of stochastic optimization frameworks often designed for modeling other complex, non-biological systems. These frameworks, though less mature than MD and MC, are summarized here in the interest of introducing readers to interesting complementary ideas. Algorithmic advances, whether to extend the applicability of MD- and MC-based frameworks or adapt other frameworks for macromolecular modeling, now allow predicting native structures of given protein amino-acid sequences [117–120], mapping equilibrium ensembles, structures spaces and underlying energy landscapes of macromolecules [6,8,121–126], revealing detailed transitions between stable and meta-stable structures [127–134], modeling binding and docking reactions [135–137], revealing not only equilibrium structures of bound protein-ligand or protein-protein assemblies but also calculating association and disassociation rates [138,139], and more.

This review aims to provide an overview of such advances. Given the rapidly growing body of research in macromolecular modeling, aiming to provide an exhaustive review would be a task in futility. For instance, while the development of molecular force fields is recognized as crucial to accurate modeling [140,141], this review does not focus on force field development. Other important contributions due to the development of ever-accurate coarse-grained representations of macromolecules, solvent models, and multiscaling techniques are acknowledged, but the reader is referred to existing comprehensive reviews on these topics [76,142–144]. Instead, this review focuses on sampling methods for the exploration of macromolecular structure spaces and underlying energy surfaces for the purpose of characterizing equilibrium structure and dynamics. This focus is warranted due to the recognition that sampling remains a problem [102,128,145]. The goal is to introduce a broad audience of researchers both to most recent and exciting research from an application point of view, as well as highlight important algorithmic contributions responsible for recent advancements in modeling macromolecular structure and dynamics.

## Recent Applications Made Possible by Hardware and Algorithmic Advancements

There is by now a wealth of computational studies aimed at extracting information on equilibrium structures and dynamics of macromolecules in molecular assemblies or isolation. Non-MD based studies can extract information about thermodynamically stable or meta-stable structures while foregoing simulations of a system’s dynamics. On the other hand, MD-based studies readily provide information on the dynamics but can only elucidate structures accessible within the time of the simulation. While non-MD based methods have made it possible to predict, for instance, biologically active structures of proteins given their amino-acid sequences, a problem known as de novo structure prediction, only MD-based methods can provide detailed information on protein folding and unfolding. Different aspects of protein-ligand binding, protein-DNA, protein-protein docking, equilibrium fluctuations, structure prediction, folding, and unfolding can be modeled with MD and non-MD methods.

Disparate time scales are involved in macromolecular dynamics, and they constitute the main challenge in describing macromolecular dynamics in fullness and detail via MD-based simulations. For instance, bond vibrations occur on the femtosecond time scale, solvent effects take anywhere from a few picoseconds up to a few nanoseconds, transitions in side-chain rotation and secondary structure occur on the 10–100 nanosecond time scale, large global structural transitions can occur on the microsecond time scale, ligand binding and allosteric regulation are usually on the millisecond time scale, and protein folding takes anywhere from a few microseconds to a few seconds, depending on protein size. In extreme cases, natural ligand and drug binding is a much longer event that can occur on the hours scale [146].

Despite such challenges, much progress has been made. Equilibrium, atomistic, MD simulations can reproduce in detail microsecond-long folding events for small proteins on specially-designed supercomputers [104,105,147,148]. Protein-ligand binding with full ligand flexibility and protein flexibility limited to the binding site can be simulated up to 100 microseconds [146,149]. Brownian dynamics simulations can capture events that occur in the microsecond time scale; when coupled with enhanced sampling techniques, these simulations have been reported to capture slow events of large proteins binding and sliding on DNA at 25 microseconds at a coarse resolution [150]. Longer simulations of an estimated time scale of more than 48 milliseconds of the *lac* repressor sliding on DNA have been reported via atomistic MD in explicit solvent [151].

Coarse-grained modeling and longer time steps can can further increase time scales but often at the cost of essential details [152]. However, multiscale MC simulations have been reported to allow studying in detail processes that occur in the range of milliseconds [76,78]. Organizations of short MD or MC trajectories in Markov state models (MSMs) can extract precious information on structure and dynamics for events that occur on longer time scales, from a few milliseconds to a few seconds [146,153].

In the following we provide a short overview of the current applications pursued by MD and non-MD methods without describing in detail the algorithmic ingredients of such methods. We highlight key examples where recent advances in MD and non-MD methods have made it possible to address problems and systems not possible before due to the large spatial and time scales involved. Descriptions of the algorithmic ingredients responsible for such computational advancements follow.

### Simulation and Modeling of Macromolecular Interactions

Simulating interactions of macromolecules with other macromolecules or small molecules is important to understand the molecular basis of mechanisms in the healthy and diseased cell. Typically, three categories of interactions are of interest to researchers: those of a protein with a small ligand, those of a protein with another protein, and those of a protein with other molecular systems that include DNA, RNA, and membranes. These specific applications can be approached in two different ways. One considers simply the problem of predicting the three-dimensional native structure of the complexed system from knowledge of the structures of the unbound units, whereas the other additionally simulates the process of the units diffusing towards and then binding with one another. For the problem of structure prediction, non-MD based methods are currently the norm. They include algorithms enhancing MC or adapting other stochastic optimization frameworks under the umbrella of evolutionary computation. For the problem of actually simulating the dynamics of interacting units, MD-based studies provide more detail but typically require more computational resources or algorithmic enhancements in order to surpass the long time scale often needed for a complexation (binding) event to occur.

One of the challenges with modeling and simulating macromolecular interactions with other small molecules or macromolecules is the possibility of induced fit. Induced fit, introduced by Koshland in [154], refers to the mechanism of an initially loose complex that induces a conformational change in either one or all loosely bound units, which then triggers a cascade of rearrangements ultimately resulting in a tighter-bound complex. The induced fit mechanism seems to question the idea that structure-guided studies can focus on shape complementarity first, but many wet-laboratory studies, as well as the success of complementarity-driven methods, have demonstrated that induced fit cannot describe all binding events [155].

In response, inspired by the free energy landscape view presented by Frauenfelder and Wolynes [13,27], Nussinov and colleagues proposed a new concept to explain binding events, that of conformational selection, also known as population shift [156–158]. Conformational selection refers to the idea that all conformational states of an unbound unit are present and accessible by the bound unit. The binding or docking event causes a shift in the populations observed in the unbound ensembles towards the specific bound conformational state. Though Nussinov and colleagues were inspired by the free energy landscape view of Frauenfelder and Wolynes, it is worth noting that the conformational selection model is a generalization of a much earlier model, the Monod-Wyman-Changeaux (MWC) model [159]. The MWC model, also known as the concerted or symmetry model, proposed the idea that regulated proteins exist in different interconvertible states in the absence of any regulator, and that the ratio of the different states is determined by the thermal equilibrium. The MWC model has been credited with introducing the concept of conformational equilibrium and selection by ligand binding, though in its original formulation the model was restricted to two distinct symmetric states and to proteins made up of identical subunits.

The review in [23] summarizes many studies that observe conformational selection for protein-ligand, protein-protein, protein-DNA, protein-RNA and RNA-ligand interactions. We highlight work in [160], where unfolded structures of uncomplexed ubiquitin in explicit solvent were subjected simultaneously to restraints from NMR Nuclear Overhauser Effect (NOE) and Residual Dipolar Coupling (RDC) data comprising solution dynamics up to microseconds. The obtained ensemble of structures covered the structural homogeneity observed in 46 crystal structures of ubiquitin at the time; the majority of the crystal structures were in complex with other proteins. These results suggest that conformational selection rather than induced fit suffices to explain the molecular recognition dynamics of ubiquitin.

While at face value the concepts of induced fit and conformational selection appear mutually exclusive, studies have shown that versions of each are indeed observed; for instance, conformational selection is usually followed by slight conformational adjustments. In 2010, Nussinov and colleagues presented an extended view of binding events where conformational selection and induced fit were seen as complementary to each other [161]. In many cases, following conformational selection, minor adjustments of side chains and backbone are observed to take place to optimize interactions [161]. Based on such observations, extended models have been proposed that combine conformational selection, induced fit, and the classical lock-and-key mechanisms [162]. A better understanding of contributions of each of these three mechanisms has contributed over the years to several effective methods for modeling and simulating binding and docking events. A detailed review in the context of protein-ligand binding for structure-based drug discovery is presented in [163].

The overview below summarizes methods based on the lock-and-key mechanism, as well as methods based on the induced-fit and conformational selection mechanisms. While the lock-and-key mechanism allows disregarding flexibility, the other mechanisms clearly make the case for modeling the flexibility of the units participating in the complexation event. While the induced-fit mechanism seems to suggest that only MD-based methods can describe a complexation event, the conformational selection mechanism has inspired many non-MD methods to integrate flexibility during or prior to complexation, thus contributing to a rich and still growing literature. In the following we provide an overview of this work, guided by applications on protein-ligand binding, protein-protein docking, and protein-DNA docking.

#### Protein-ligand binding

In protein-ligand binding, the structure prediction problem involves predicting both the binding site, unless this is known, the pose of the ligand, and its configuration. Established and widely-adopted software now exist and include DOCK [164], FlexX [165,166], GOLD [167,168], Autodock [169–171], Glide [172], RosettaLigand [173,174], SwissDock [175], Surflex-Dock [176], DOCKLASP [177], rDock [178], istar [179], and more. The majority of existing software employ evolutionary algorithms that approach the problem of protein-ligand binding under stochastic optimization, where the goal is to find the lowest-energy structure of the complex of bound units. Evolutionary algorithms have been demonstrated more effective than other MD- or MC-based algorithms at finding the lowest-energy binding pose (position and orientation) and configuration of a ligand on a macromolecule. For instance, while earlier versions of the well-known Autodock software employed MC simulated annealing (MC-SA), Autodock 3.0.5 and onwards switched to the Lamarckian Genetic Algorithm (GA) due its higher efficiency and robustness over the MC-SA of earlier versions for binding flexible ligands onto rigid receptors [180].

The superiority of evolutionary algorithms for binding flexible ligands onto rigid receptors is additionally demonstrated in a high-throughput screening setting. In this context, we note representative work in the Caflisch laboratory [181], where a set of publicly-available tools have been developed for high-throughput screening of large sets of small ligand molecules by fragment-based docking for the purpose of computer-assisted drug discovery (CADD). The high-throughput setting is made possible due to a fast decomposition of a flexible ligand into rigid fragments, fast docking and evaluation of binding free energy of docked fragments, and efficient docking of a full flexible ligand through a GA rapidly searching over poses of fragment triplets and evaluating poses with an efficient scoring function. Fragment-based docking can be traced back to Karplus, whose work with Miranker on the minimization of multiple copies of functional groups in the MCSS force field is considered the first fragment-based procedure for drug discovery [182].

Fragment-based high-throughput binding is leading to significant advances in CADD. For instance, recent work in [183] identifies inhibitor chemotypes for the EphA3 tyrosine kinase, a transmembrane protein belonging to the class of erythropoietin-producing hepatocellular receptors with deregulations implicated in severe human pathologies such as atherosclerosis, diabetes, and Alzheimer’s disease.

While the majority of protein-ligand binding software can handle flexible ligands, the computational costs that would be incurred by fully flexible receptors remain impractical in most settings. Fortunately, a significant number of binding modes fall under the lock-and-key mechanism, which has been demonstrated effective in cases of predicting structures of enzyme-inhibitor complexes with largely static binding interfaces [184–188]. As expected, however, rigid receptor docking algorithms are ineffective in cases of induced fit, where structural flexibility during binding is not limited to the ligand.

To take into account ligand and receptor flexibility without incurring impractical computational costs, many protein-ligand binding algorithms implement soft docking, where some overlap between the flexible, bound ligand and the rigid receptor is allowed during docking. Unfavorable interactions due to the overlap are resolved in a post-processing stage on selected bound complexes, effectively providing some localized flexibility to the bound receptor. This approach is practical and warranted in settings where the goal is to screen large libraries of potential drug compounds [189–191]. An extensive review of the unique challenges in these settings can be found in [163,192].

One way to control computational cost while taking into account both ligand and receptor flexibility is by limiting flexibility to specific dihedral angles [193–197]. Typically, existing approaches limit receptor flexibility to side-chain and/or backbone bonds of receptor amino acids on or near the binding site.

Other methods attempt to take into account full receptor flexibility without explicitly modeling it during binding. These methods, known as ensemble or conformer docking, obtain an ensemble of low-energy conformations/conformers of the receptor prior to the binding simulation [198]. The ensemble is obtained via any conformational sampling methods, whether MD- or non-MD based (reviewed below). The ligand or a library of ligands are then bound to each of the receptor conformers [199]. While effective at controlling computational cost, these methods are limited in what aspects of flexibility they model [200]. It is worth noting that they make use of the conformational selection principle of which there is now increasing evidence [201].

Methods that consider full receptor flexibility and go beyond ensemble docking exist, and are based on MC or MD. MC-based methods are represented by the RosettaLigand software [173,174]. Work in [202] employs long, unbiased MD simulations to simulate the physical process by which a ligand diffuses and then binds a protein target. Studies on specific protein-ligand complexes provide an opportunity for MD-based methods to reveal the kinetics of ligand-receptor interactions and estimate binding affinities from a large number of MD simulations of the binding process. Yet, even in such studies computational cost needs to be controlled, as binding can be too slow to observe on the time scales routinely accessible via MD [203].

Given the time scale challenge, many enhanced sampling strategies have been proposed for MD simulations. These include accelerated MD, replica-exchange MD, umbrella sampling MD, and metadynamics methods [8,149,203–206]. Replica exchange MD and metadynamics methods are among the most popular to simulate binding. To control computational cost, the simulation is limited to the immediate binding and unbinding events. To discourage spending computational resources on the diffusion process, the ligand is either tethered (through distance restraints) to the receptor, or many short MD simulations are conducted at various placements of the ligand relative to the receptor. In the former, explicit geometric restraints are enforced on the ligand to keep it within the binding volume and save the MD simulation from wasting precious computational time on simulating the diffusion process [149]. In the latter, the sampled receptor and ligand configurations are organized in an MSM, which allows obtaining estimates of association and disassociation rates [139]. Other approaches include the powerful self-guided Langevin dynamics method and the accelerated adaptive integration method, among others. A description of these methods and others is provided later in this review. In summary, the goal of all these methods is to enhance sampling of the receptor and ligand poses so that the binding event can be observed within a reasonable computational budget.

Here we highlight some successful protein-ligand binding simulations. One concerns the GTP and GDP nucleotide binding that is accompanied with a conformational switch in the Ras and Rho proteins, which was studied in [207] due to the central role of these proteins in cell growth regulation and a variety of human cancers [122]. In [207], MD is used to simulate the ligand-free Ras and Rho proteins. In the absence of the ligand, these proteins show intrinsic flexibility and are able to convert between different conformations. The presence of the nucleotide restricts the conformation space accessible by the GTP-bound structure. Significant coupling is observed in the bound state between motions on the nucleotide-binding site and motions of the membrane-interacting C-terminus via the highly flexible loop 3. The importance of this loop was originally suggested in [208]. Classic MD simulations with a double loop 3 mutant of Ras confer greater flexibility during conformational switching. This provides evidence that loop 3 may represent a potential allosteric site in Ras and other monomeric G-protein coupled receptors. This information, pieced together from various studies, is valuable for structure-based drug design, because it highlights relevant receptor structures for CADD [163].

Another successful example of the utility of computational methods for protein-ligand binding concerns drug prediction for the influenza virus. Several inhibitors have been widely used as anti-influenza drugs. However, due to naturally-occurring drug-resistant mutations [209], their inhibition ability has gradually decreased. The family of influenza virus proteins, like M2, H1-H9, attaches itself to sialic acids on the surface of epithelial cells of the upper respiratory tract of the host using its own proteins that cover the surface of the virus, hemagglutinin and neuraminidase [210,211]. Inhibitors bind to the active sites of hemagglutinin and neuraminidase, preventing linkage of the virus to epithelial cells.

**Fig 1:**
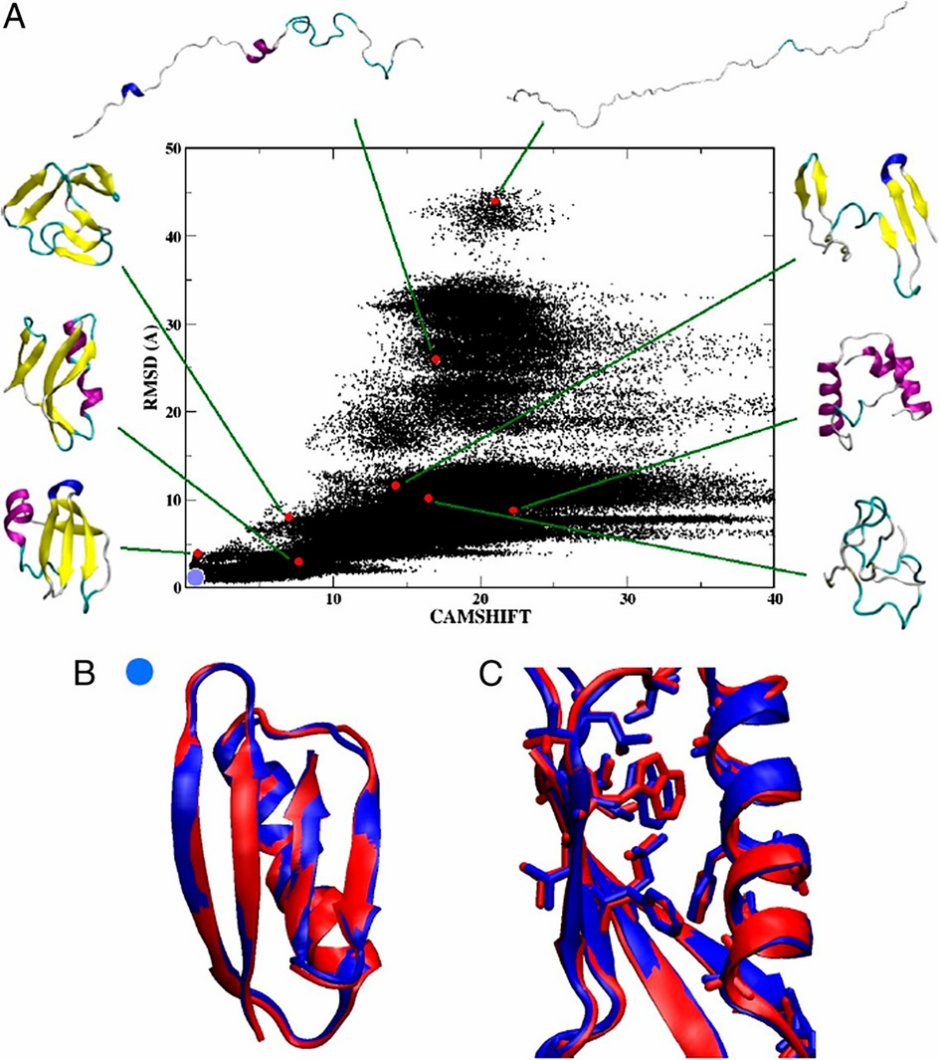
Free-energy landscape of GB3 obtained with work in [302] using chemical shifts as collective variables. Panel A shows a two-dimensional projection of sampled conformations. The *x*-axis shows values of the CamShift collective variables for each conformation, which measures the difference between the wet-laboratory and calculated chemical shifts for the backbone. The *y*-axis shows the backbone RMSD between each conformation and the reference structure (PDB ID 2oed). Some selected conformations, from extended to compact, are highlighted, drawn with the Visual Molecular Dynamics (VMD) software [303]. Panel B shows a conformation with the lowest backbone RMSD (0.5 Å) from the reference structure. Such native-like conformations are visited multiple times by the method. Panel C draws hydrophobic side chains to illustrate that the internal packing of these side chains is practically identical to that observed in the reference structure. This figure is reproduced with permission of the executive editor of *PNAS* from article Granata et al., 2013 [302].

Protein-ligand docking via MD simulations is being used to model inhibitor binding to the influenza virus (or only the surface proteins hemagglutinin and neuraminidase). One group of methods focuses on finding new inhibitors (ligands) that can bind to the continuously mutating hemagglutinin and neuraminidase active sites [210,211]. Representative findings are illustrated in Fig 1.

In particular, work in [211] focuses on finding new inhibitors for hemagglutinin. Several ligands are considered to bind to the hemagglutinin H5 and H7 trimers. The exposed position of the binding site is used to guide the development of a trimeric ligand with a centrally positioned core structure with radial topology. The core structure of the ligands mimicks the C3 symmetry of the trimers. A specific ligand, referred to as ligand 1, is found to bind to all three binding sites on H5 (deposited in the PDB under PDB ID 3M5G) at two different times of an MD simulation. Motion is predominantly found at the core structure, while all three sialic acid residues remain in their binding site during the simulation, indicating that 1 is also a good ligand for H7. Ligand 1 also has a *K*_*D*_ in the high nanomolar range and is therefore a compound with one of the best reported affinities.

Another group of methods aims to modify (add new residues or suggest mutations) to already known inhibitors in order to increase their binding ability [212,213]. Finally, some methods focus on calculating binding free energies by quantum mechanics/molecular mechanics simulations to predict binding abilities of possible inhibitors [214]. The combined result of all these methods has been to suggest a mechanism through which the inhibitor-virus binding can significantly influence viral neutralization.

In addition to MD simulation methods, we draw attention to Brownian Dynamics methods [215], which have been employed to simulate protein-ligand [216] and protein-protein [217,218] binding. In these methods, the net force experienced by a modeled particle contains a random element, which models the implicit interactions with solvent molecules. The norm of the random element is chosen from a probability distribution function that is a solution to the Einstein diffusion equation (a list of already built probability distribution functions can be found in [219]). By coarse-graining out the fast motions, Brownian dynamics methods can simulate longer time scales than can be typically approached in a classic MD simulation [220]. However, the particle-based part still necessitates using relatively small time steps for an accurate description of the particle interactions. The Reaction Before Move method determines reaction probability functions that extend time steps and further speed up such simulations [219].

The importance of accounting for receptor flexibility in protein-ligand binding is further appreciated in light of allosteric effects. Allostery refers to couplings between the active site and a regulatory, allosteric site, which is typically far away from the active site, but causes chemical and/or physical changes in the active site that affect binding. A detailed review of all observed interactions between allosteric and binding sites is presented in [221]. The structural view of allostery considers interactions among residues responsible for the allosteric coupling between allosteric and binding sites. Uncovering allosteric communication among residues is becoming increasingly important in CADD, as residues that mediate the allosteric communication may make for druggable binding sites. Many methods are devoted to uncovering allosteric communication, and a review of such methods is presented in [137]. Successful methods include early ones based mainly on topological analyses of structures resolved in the wet laboratory, such as graph theory, statistical coupling analysis, and perturbation algorithms [222–227], and methods based on analyses of simulation trajectories. While MD and enhanced versions of MD-based methods are used for the simulations, the analysis is conducted with normal mode analysis (NMA) [228–230], correlation matrices [231–233], community-network analysis [234], mutual information [235], and dynamical network analysis [236–238]. MC-based methods have also been applied. The MCPath method introduced in [239] models a receptor as a weighted network of interacting residues and builds an MC trajectory by repeatedly applying MC moves that directly propagate a signal between two interacting residues. MCPath is able to uncover allostery pathways as well as allostery sites.

#### Protein-nucleic acid and protein-protein docking

The computational challenges incurred when modeling protein-ligand binding grow more severe when modeling interactions between macromolecules due to the much larger spatial scales involved. Most current research addresses only the dimeric setting, where the number of bound units is limited to two. In addition, the majority of methods applied to the pairwise docking setting are non-MD based methods focused on obtaining the native structure of the complex without information on the kinetics of the docking process. Methods implementing MC or evolutionary algorithms are by now the most popular. This is not surprising, given the overwhelming number of atoms whose motions would have to be followed in an MD simulation. Specific MD-based studies on dimeric systems of known proteins exist, and typically some information is employed from wet-laboratory studies on the docking site to orient the units favorably and additionally tether them to each other so as to steer the simulation towards the docking event [240,241]. In general, however, even when foregoing kinetics, predicting the correct native structure of the bound units remains challenging.

Computational research in structure prediction for macromolecular pairwise docking is active, and there are now many methods [242–255] driven by the community-wide CAPRI experiment [256,257]. The focused computational setting of a protein dimer has allowed the application of demanding energy-driven optimization methods and even modeling of structural flexibility for high-accuracy docking [243,251,258]. In the light of variable interfaces, such as antibody-antigen interfaces [259], accounting for flexibility is key but exceptionally expensive. Methods such as RosettaDock [260] allow full flexibility and employ various models of increasing detail (from low-resolution, to centroid-mode, coarse-grained, and then all-atom). RosettaDock has been reported to achieve docking funnels for 63% of antibody-antigen targets, 62% of enzyme-inhibitor targets, and 35% of other targets; funnels are achieved on only 14% of targets deemed difficult, on which substantial conformational changes are expected to accompany docking [261]. Other methods that consider ensemble docking have also been applied, though with limited success due to the difficulty of obtaining a conformational ensemble representative of the intrinsic structural flexibility of a macromolecule [262].

Several CAPRI summaries make the case that high-accuracy pairwise docking is to remain challenging for the near future [257,263,264]. There is great difficulty, for instance, in locating the native interaction interface or even part of it, with top methods shown to predict only 30%–58% of the correct interface in any given target [257]. An energy-based treatment is not guaranteed to drive the optimization process towards the right interface. Much research is invested in this direction. Machine learning methods, though not the focus of this review, are showing promise in elucidating features of native interaction interfaces so as to bypass the employment of interaction energy functions at a global layer [265–268]. For instance, work in [269] proposes a learned model to be used as a top filter to label sampled protein-protein dimers before attempting to refine them with more accurate and computationally costly interaction energy functions. Rather than employing information from machine learning models, methods such as HADDOCK [243], the Integrative Modeling Platform (IMP) [270] and others [271,272], employ wet-laboratory data to restrict sampling of bound conformations to those that reproduce the wet-laboratory data. Work in [273] uses chemical shifts from NMR to predict conformational changes upon complex formation in a class of engineered binding proteins known as affibodies. Similarly, Haddock also restricts sampling through NMR chemical shifts [243], whereas the IMP software provides more versatility by allowing the integration of different types of wet-laboratory, biochemical and biophysical data and the employment of models of various resolutions [270]. It is worth noting that, while the majority of protein-protein docking algorithms are restricted to the dimeric setting, the IMP software allows modeling multimeric assemblies of an arbitrary number of units. Work in [274], for instance, reveals the native structure of the nuclear pore complex, a 50 MDA complex comprised of 456 proteins. Work in [275] reveals a higher-resolution structure of a heptameric module in the yeast NPC by satisfying spatial restraints derived from negative-stain electron microscopy and protein domain-mapping data.

While wet-laboratory techniques such as X-ray crystallography can provide high-resolution structures for protein-protein dimers and even multimers, protein-DNA dimers are typically difficult to crystallize. There is great need for docking methods to reveal both binding mechanisms and final bound structures of protein-DNA complexes. In contrast to the diversity of protein-protein interaction interfaces, protein-DNA interaction interfaces often exhibit conserved sequence motifs and are thus accurately detected with machine learning techniques [276,277]. Knowledge, even if partial, of the interaction interface has greatly helped the applicability of docking methods for protein-DNA binding [278,279]. Haddock, for instance, already a top protein-protein docking method, has been demonstrated effective for protein-DNA docking [280]. By now, comprehensive maps of protein-DNA binding landscapes have been put together for the largest class of metazoan DNA-binding domains, known as zinc fingers [281]. These landscapes are essential to support efforts to determine, predict, and engineer DNA-binding specificities. For instance, work in [282] studying interactions that proteins make with nucleic acids, small molecules, ions, and peptides reveals genes that are rich in mutations in the binding sites of proteins for which they encode and are thus functionally-important in cancer.

The setting of modeling macromolecular interactions naturally suggests expanding the focus beyond dimeric docking to multimeric docking. Elucidating structural details of oligomers suggested by wet-laboratory studies is indeed key to advancing further research on the role of oligomerization in the healthy and diseased cell [283,284] and is expected to keep motivating the design of algorithms for multimeric docking. Computationally-demanding optimization and willingness to spend significant computational resources on a dimeric assembly make application of current pairwise docking methods to protein assemblies of an arbitrary number of units impractical. Adaptations of these methods to extend their applicability to the multimeric setting are neither trivial nor obvious.

Early work by Nussinov and colleagues introduced a greedy, systematic algorithm, CombDock, for the problem of multimeric docking [285,286]. The algorithm is general and can handle heteromeric and asymmetric complexes but is challenged by the combinatorial explosion in the number of dimensions of the space of configurations with increasing number of units. Other following work narrows the focus to symmetric complexes and applies search and bound techniques from AI with additional information of distance-based constraints from NMR to control the size of the search space [287–291]. Work in the Sali lab, culminating in the IMP software [270], focuses exclusively on the setting where integration of wet-laboratory data is key to narrow the search space and model assemblies of hundreds of units at a low resolution. Research on multimeric docking in the absence of wet-laboratory data is sparse.

In [292], an evolutionary algorithm, Multi-LZerD, is proposed that operates in the absence of wet-laboratory data but is guided by interaction energy. Its success varies with complex size. The mixed results obtained by Multi-LZerD reflect the mixed state of the art in multimeric docking. In addition to successful cases, where the native multimeric structure is reproduced, Multi-LZerD reports in various cases decoys that do not reproduce the known native structures. While the decoys can be as far as 23.59 Å away from a particular native structure, typically, the decoys contain correct subcomplexes within 4.0 Å. It is worth noting that the evolutionary algorithm is also computationally demanding. Time concerns as well as the quality of current predictions suggest that there is much room for improvement in multimeric docking.

### Modeling of Macromolecular Structural Flexibility

Modeling the structural flexibility of uncomplexed proteins is key not only to allow application of methods such as ensemble docking to the protein-ligand and protein-protein docking problems, but also to obtain detailed information on the role of protein sequence on structure, dynamics, and function. While it is in principle very difficult to map the entire conformation space and underlying energy landscape of a protein sequence, many methods are dedicated to specialized sub-problems. For instance, literature is rich in methods that obtain a sample-based representation of the equilibrium conformation ensemble of a protein. Other methods extend this characterization to proteins that exhibit not only local fluctuations around an average, wet-laboratory, equilibrium structure but indeed are characterized by multi-basin landscapes where distinct structural states have comparable Boltzmann probabilities. Many methods focus on such proteins and particularly on modeling transitions between similarly stable structural states as a way to obtain information on function modulation and changes to function upon sequence mutations. Other methods are dedicated to capturing allosteric regulation and identifying coupled motions not in the vicinity of binding sites. Yet others focus on obtaining detailed structural characterizations of meta-stable states and other states present at low populations, even in natively unfolded proteins, as a way to understand aggregation, misfunction, and other disorders. In the following we provide an overview of these applications, highlighting selected ones to showcase current capabilities.

#### Sampling of equilibrium conformation ensembles

In principle, complete information about structure and dynamics can be obtained from mapping the energy landscape of a given macromolecular sequence. Despite advances in atomistic MD simulations, this remains an insurmountable computational task but for the smallest peptides. As such, we separate here the discussion of work on sampling the ensemble of folded conformations from work that focuses on protein folding and/or structure prediction. Methods that initiate their search for other conformations of the equilibrium ensemble from one or a few given conformations or wet-laboratory data are in practice more efficient and have been employed to characterize both local fluctuations and large-scale motions connecting conformations of the equilibrium or native state in proteins.

We highlight here work that builds over the MD or MC frameworks but restricts sampling in conformation space to regions that reproduce wet-laboratory data. In particular, chemical shifts, which are NMR observables measured under a wide range of conditions and with great accuracy, are proving very useful to methods in generating conformation ensembles that capture macromolecular dynamics in solution. For instance, work in [293,294] uses chemical shifts for backbone atoms as restraints in a replica-averaged MD simulation. Work in [295] additionally incorporates NMR chemical shifts for side chains and demonstrates as a result great agreement between reconstructed conformation ensembles and wet-laboratory data, thus improving the accuracy of computational methods and ability to make useful predictions on macromolecular structure and dynamics. Work in [296] characterizes in detail the native conformation ensemble of the src-SH3 domain and role of water. Work in [297] incorporates diffuse X-ray scattering data to characterize the conformational dynamics of a crystalline protein at the μs time scale. In other works [129,298–301], restraints from wet-laboratory data are employed to improve the quality and thus accuracy of simulation methods.

In the above works, the main idea is to incorporate the wet-laboratory data into a restraint potential that is added to a molecular mechanics force field. In [302], the free energy landscapes of small-size proteins are characterized by using the NMR chemical shifts as collective variables, also known as reaction coordinates in slight abuse of terminology) in metadynamics simulations. Doing so enhances sampling and allows visiting multiple free energy minima not typically reached by classic MD simulations [302]. The free-energy landscape reconstructed for the third Ig-binding domain of protein G from streptococcal bacteria (GB3) in [302] is shown in Fig 1. In [34], the interdomain motions of the hen lysozome are characterized using RDC data to restrain MD simulations.

The idea of incorporating wet-laboratory data in energy functions, thus resulting in pseudo-energy functions, has been popular for over a decade and demonstrated effective not only in the context of MD sampling but also of MC sampling for reconstructing equilibrium conformation ensembles (and even structure prediction, as we review below). For instance, work in [304] demonstrates that the use of replica-averaged structural restraints in MD simulations with a particular force field and a set of wet-laboratory data can provide an accurate approximation of the Boltzmann distribution of a macromolecule. Though NMR chemical shifts are proving more general at capturing the extensive equilibrium dynamics, NOE, RDCs, *S*^2^ order parameters, J couplings, and hydrogen exchange data have been used to restrain both MD and MC sampling and obtain detailed information on structure and dynamics of equilibrium states and transition states in proteins [32,35,36,305–313]. The main advantage of incorporating wet-laboratory data is to remedy inherent biases in force fields and guide the sampling of the conformation space to relevant regions. Concerns of accuracy then entirely shift on the breadth of sampling and the generality of the wet-laboratory data to capture the equilibrium dynamics. Recent work affirms that NMR chemical shifts are very powerful in this regard, and combined with enhanced sampling techniques for MD and MC, allow sampling equilibrium conformation ensembles and thus faithfully capturing equilibrium dynamics [273,293–295,314]. It is worth noting that there is great difficulty in the wet laboratory in calculating chemical shifts, J-couplings, and other measurements from structures. A central issue is the large uncertainty inherent in such calculations. One way in which computational methods address this issue is by integrating different types of experimental data [315,316].

Other non-MD based methods have also been applied, particularly to model internal, equilibrium structural fluctuations of uncomplexed proteins. These methods, such as CONCOORD [317], FIRST/FRODA [318,319], and PEM [320–322], are designed to rapidly populate the conformation space in a neighborhood around a given structure. They typically restrict an underlying stochastic optimization process based on MC or other non-MD algorithms with geometric constraints. The constraints are obtained from analysis of a given structure resolved in the wet laboratory and considered representative of the equilibrium conformation ensemble. For instance, work in [317] repeatedly generates and then corrects random conformations until a set of upper and lower geometric bounds obtained from the given structure are satisfied. Work in [318,319,323] is based on constraint theory and models a given structure as a bar and joint framework. This model allows employing rigidity analysis to reveal underconstrained backbone angles on which sampling focuses to obtain inherent internal fluctuations. Work in [320–322] is based on the treatment of inverse kinematics in robotics and computes local fluctuations by restricting ends of consecutive overlapping segments of the protein chain to positions in the given structure.

Structure-guided methods, while useful at probing regions of a conformation space around a given structure, are not readily useful when the goal is to populate a highly heterogeneous equilibrium ensemble for which there may not be sufficient representative structures. On such proteins, often referred to as multi-basin proteins due to the existence of potentially comparably-deep basins in the free-energy landscape, large conformational changes are observed between basins. Detailed reconstruction of the energy landscape of a protein is at this point challenging. Non-MD methods have been devised and applied to capture thermodynamically stable and semi-stable structural states in multi-basin proteins [125,126]. In [126], an MC-SA method is devised that employs multiple scales of representational detail and the fragment replacement technique popular in de novo structure prediction to map the energy landscape of the uncomplexed adenylate kinase (AdK) protein. However, only a subset of the known states are captured, pointing to the general challenge to devise enhance sampling techniques capable of reconstructing energy landscapes of proteins in the absence of any a priori information. Fortunately, significant, even if partial, information now exists from wet-laboratory techniques on stable or semi-stable states of wildtype and variant sequences of proteins. The method in [324] exploits this information to define a lower-dimensional search space on which extensive sampling can be afforded to reveal diverse thermodynamically stable and semi-stable structural states. We note that such states are stable in the lower-dimensional space, as no information is available on the true potential energy surface.

While MD-based methods are challenged in a de novo setting, they are particularly suitable to reveal the detailed structural transitions connecting two known structural states. Providing detailed transitions is key to understanding the mechanistic basis of several disorders linked to transition-modifying mutations. This promise has attracted other non-MD methods that can sample conformational paths connecting two structural states of interest without direct time-scale information on the transition. In the following we provide an overview of work in modeling and simulating structural transitions.

### Modeling of Structural Transitions

Many proteins undergo large conformational changes that allow them to tune their biological function by transitioning between different structural states, effectively acting as dynamic molecular machines [325]. Since it is generally difficult for wet-laboratory techniques to elucidate a transition in terms of intermediate conformations (though successful examples exist [326]), computational techniques provide an alternative approach [327]. However, transition trajectories may span multiple length and time scales, connecting structural states more than 100Å apart. This length scale is up to two orders of magnitude larger than a typical interatomic distance of 2 Å. Transitions can also demand micro-millisecond time scales, which is six to 12 orders of magnitude larger than typical atomic oscillations of the femto-pico second time scale.

Typically, three types of methods are applied to model structural transitions, MD-based methods, morphing-based methods, and robotics-inspired methods.

MD-based methods typically have to employ powerful algorithmic enhancements to surpass high-energy barriers in structural transitions. However, cases exist when classic MD methods have been able to capture spontaneous transitions of allosteric proteins by monitoring the structural relaxation upon removal of the bound molecule from the binding pocket [328,329]. These works further highlight the utility of the conformational selection or population shift principle, as removal of the bound molecule prompts spontaneous movement towards a new equilibrium state.

In cases of high-energy barriers, biased or targeted MD methods are useful to expedite transitions between given structures [127,330], but the concern with such methods is that the transition trajectory may not correspond to the true one, as these methods modify the underlying energy landscape; the order of events in transition paths computed via targeted MD methods depends on the direction in which the MD simulations are performed. For example, an application of biased MD to capture transitions of Ras between its active and inactive structures resulted in unrealistic, high-energy structures [330]. It is worth noting, however, that recent work in [331] has proposed a technique to remove the length-scale bias from targeted MD simulations. Essentially, the technique formulates local restraints, each acting on a small connected portion of the protein sequence, resulting in a number of potentials that are then used in targeted MD simulations. The technique has been demonstrated effective on an application to the open ↔ closed transition in the protein calmodulin. The free energy barriers associated with the computed paths have been shown comparable to those obtained with a finite-temperature string method.

**Fig 2:**
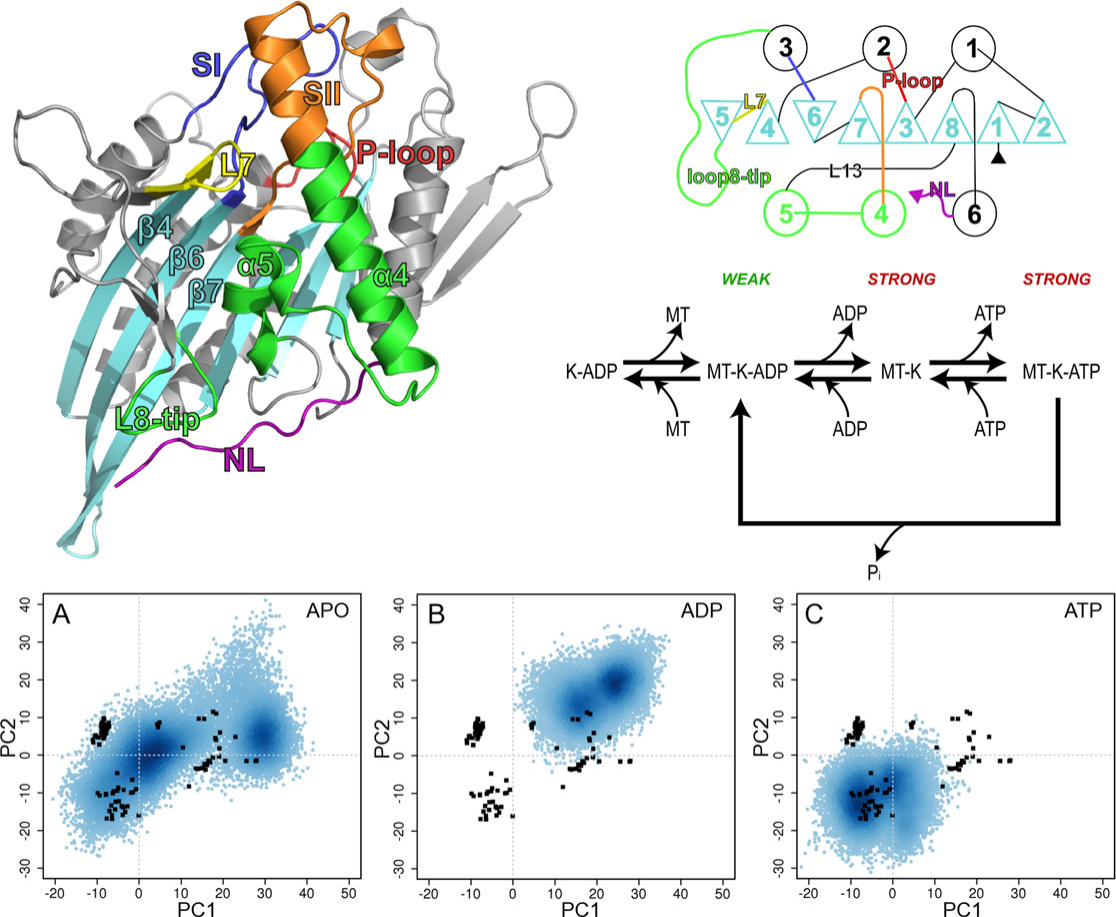
Probing of coupled motions in the Eg5 kinesin motor domains in [91] through accelerated MD simulations. The top panel shows the structure and catalytic cycle of the kinesin motor domain. The ATPase catalytic site sits at the top of the *β*-sheet, flanked by three highly-conserved loops (P-loop, SI, and SII) connected to helices (also annotated) on either side of the sheet. The secondary structure topology is drawn, with *β* -strands drawn as triangles and *α*-helices as circles. The kinesin catalytic cycle is shown: Kinesin (K) has a weak affinity for the microtubule in the ADP-state. ADP release is followed by strong microtubule-binding. ATP binding may occur followed by hydrolysis and product release to regenerate the weakly-bound ADP state. The bottom panel projects conformations sampled by 200 nanosecond-long accelerated MD every 20 picoseconds on the two principal modes of motion. The latter are obtained through principal component analysis of collected X-ray structures for wildtype and variant Eg5. Three simulations are highlighted, the nucleotide-free (APO) one in (A), ADP-bound one in (B), and ATP-bound one in (C). The nucleotide-free simulation covers more of the conformation space, whereas restricted sampling is observed when Eg5 is bound to ATP or ADP. One of the conclusions in [91] is that structural changes from the ADP- to ATP-bound states which are evident in the collection of X-ray structures, are encoded in the intrinsic dynamics of the nucleotide-free motor domain; the nucleotides effectively rigidify the motor domain by narrowing the conformation space accessible by it, as evident in the restricted sampling observed through accelerated MD. This figure is reused from Scarabelli et al., 2013. CC-BY PLOS ONE [91].

In contrast to biased MD methods, accelerated MD methods do not change the entire landscape but only the relative height of the basins corresponding to the structures that need connecting with intermediate conformations [332]. Accelerated MD has been applied to several proteins to capture the transition of H-Ras between the inactive and active structural states [10], map the structural and dynamical features of kinesin motor domains [91], compute domain opening and dynamic coupling in alpha subunit of heterotrimeric G proteins [333], and more. Representative results on an application of accelerated MD for capturing the dynamics of the Eg5 kinesin motor domain are shown in Fig 2.

Even accelerated MD methods are limited in their ability to elucidate transition trajectories that cross high energy barriers [10]. In contrast, the dynamic importance sampling (DIMS) MD method [334,335] is more effective at simulating macromolecular transitions with energy barriers. In DIMS, the next conformational state sampled to obtain a transition from a state A to a state B will be chosen to satisfy the most productive movement to B and cross the energy barrier. The productive movement is indicated by a robust progress variable, the instantaneous RMSD over heavy atoms between a conformation and the target structure. DIMS is integrated in CHARMM and has been tested on several systems [336], including modeling of slow transitions in AdK [334], folding of protein A and protein G, and conformational changes in the calcium sensor S100A6, the glucose–galactose-binding protein, maltodextrin, and lactoferrin, showing good agreement between sampled intermediates and experimental data [336].

**Fig 3:**
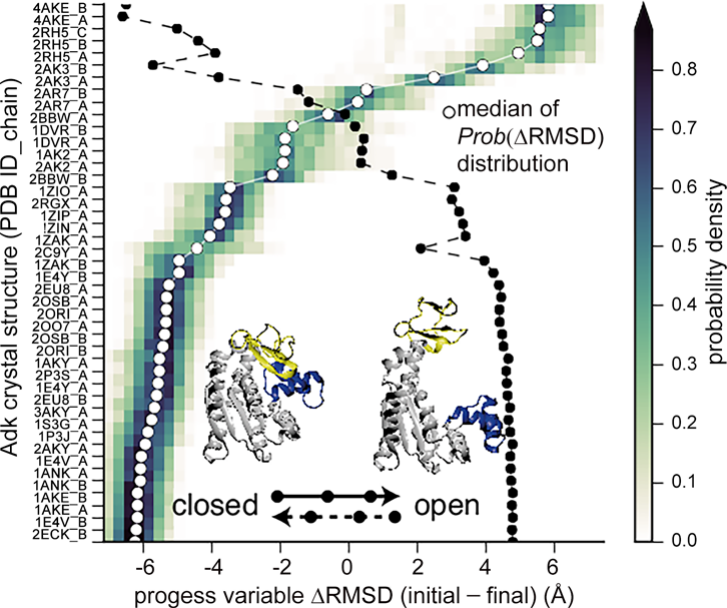
Sampling of the ensemble of closed-to-open and open-to-closed transition trajectories in AdK through the DIMS method [334]. An ensemble of 330 DIMS trajectories is compared to 45 *Escherichia coli* AdK X-ray structures. The conformations in each trajectory are projected onto a progress variable *δ*RMSD measured as the RMSD of the conformation from the closed AdK structure (PDB ID 1ake:A) minus the RMSD of the conformation from the open AdK structure (PDB ID 4ake:A). For each of the 45 collected X-ray structures and each trajectory, the conformation in the trajectory closest in backbone RMSD to an X-ray structure is recorded, and the *δ*RMSD value of the conformation along a trajectory is recorded. A probability distribution is then constructed for each X-ray structure over all DIMS trajectories to indicate where an X-ray structure is located along the simulated trajectories. The color bar indicates the probability density. The median of each distribution is marked by a white circle. The X-ray structures whose PDB IDs are listed on the *y*-axis are rank ordered based on the median. The second white line traces the location of the median when the simulations are repeated to sample open-to-closed transition trajectories. Out of 45 structures sorted by *δ*RMSD, about 24 are closed-state structures, four are open, and 17 are intermediates. This work is an example of the capability of computational methods to elucidate transitions in detail and accurately map the location of experimentally determined structures in the transitions. This figure is adapted from Beckstein et al., 2009 [334]. The image was created by O. Beckstein.

In particular, in [334], DIMS is applied to sample the ensemble of open-to-closed transitions for AdK. AdK is an enzyme that regulates the concentration of free adenylate nucleotides in the cell by catalyzing the conversion of ATP and AMP into two ADP molecules. The enzyme undergoes a large conformational change in its transition between an open and a closed structural states, and this change has been observed even in the absence of a substrate. As a result, AdK is one of the few proteins for which wet-laboratory studies have been able to capture a great number of intermediate structures populated during the open-to-closed transition. For this reason, AdK is a poster system to measure the capability of computational methods to reproduce transitions in great structural detail. Work in [334] is one of the few to provide atomistic detail, as well as reproduce and map with great accuracy the location of known intermediate structures along the transition. Representative results are shown in Fig 3.

Morphing- and string-based methods provide an alternative way to compute transition trajectories. Morphing-based methods include MolMov [337], FATCAT [338], NOMAD-Ref [339], MinAction [130], Climber [340], and more. In Climber, the interresidue distances in a given start structure are pulled towards distances in the goal structure, using harmonic restraints incorporated in a pseudo-energy function. MolMov and FATCAT interpolate linearly in Cartesian space or over rigid-body motions. NOMAD-Ref uses elastic normal modes and interpolates interresidue distances per the elastic network algorithm in [341]. MinAction solves action minimization equations at each of the provided structures assuming a harmonic potential at them. Other methods include those based on elastic network models (ENMs) [131,341], the nudged elastic band, zero- and finite-temperature string methods [340,342–347]. In particular, the string-based methods make use of the committor function to account for not generally knowing the collective variables underlying the transition [343], whereas methods based on ENMs show the ability of coarse-grained models at capturing allosteric transitions in supramolecular systems on the order of megadaltons [131]. In general, while efficient, all these methods tend to reproduce similar conformational paths in independent runs rather than provide a possibly heterogeneous ensemble of conformational paths realizing the transition.

Work in [348,349] tackles this issue of possibly high inter-run path correlations with the weighted ensemble method (WEM). WEM, originally proposed in [350], has been shown a useful enhanced sampling method for off-equilibrium and equilibrium processes. WEM uses a multiple-trajectory strategy where MC trajectories spawn new ones upon reaching new regions of the conformation space. One of the first applications of WEM to path sampling was on a 72-residue domain of the calmodulin protein. Coupled with a united residue model, WEM was able to capture the transition between the calcium-bound and calcium-free structural states and compare well with brute force simulations in a fraction of brute-force simulation time. In [349], WEM is used to investigate the mechanism of the conformational change that the 5HIR benzylhydantoin transporter Mhp1 undergoes from a state poised to bind extracellular substrates to a state that is competent to deliver substrate to the cytoplasm. WEM reveals a heterogeneous ensemble of outward-to-inward conformational paths and identifies two distinct modes of transport.

Robotics-inspired methods have also been applied to model structural transitions. They rely on deep analogies between robot motion planning and macromolecular motion simulation. In particular, the T-RRT [351] and PDST [352] methods, adapted from tree-based robot motion planning frameworks, have focused on the problem of computing conformational changes connecting two given structures in small and large proteins. While T-RRT has been shown to connect known low-energy states of the dialanine peptide (two amino acids long) [351], the PDST method has been shown to produce credible information on the order of conformational changes connecting stable structural states of large proteins (200–500 amino acids long) [352]. Both methods control the dimensionality of the conformation space by either focusing on systems with few amino acids [351] or by employing very coarse-grained representations to limit the number of modeled parameters in large proteins [352]. Work in [353] extends the capability of these frameworks to address large conformational changes in proteins, such as calmodulin and AdK, while providing high-resolution intermediate conformations by employing fragment-based moves. Other work detaches the sampling of the structure space from analysis of motions [354]. MSM-based analysis of sampled conformations is conducted to compute average properties of interest, such as expected number of transitions connecting two given structural states in lieu of direct time-scale information.

### Protein Folding and Structure Prediction

Protein folding and structure prediction are often treated as two sides of the same coin. Protein folding, however, focuses on uncovering the detailed series of conformational changes that a protein goes through from a denatured, unfolded state to its long-lived, equilibrium, folded state. The folded or native structure is the end-result of this process, but not the only goal. Indeed, there are many protein folding algorithms that employ information about the native structure in order to expedite the search for the folding mechanism. Structure prediction algorithms focus more on the end result; that is, the goal is to uncover the native, folded structure even if the process by which these methods do so does not resemble the physical folding one. In its broadest context, the protein folding problem aims to shed light on the physical code by which a protein amino-acid sequence determines the native structure, the speed with which proteins fold, and the design of effective algorithms for predicting the native structure from sequence.

An extensive review of protein folding is presented in [355]. The credit with introducing the problem to the computational biology community goes to Kendrew and coworkers, who published the first structure of a globular protein, myoglobin and showed the complexity and lack of symmetry or regularity in protein native structures [61]. Since then, a general mechanism for folding has been elusive. Various paradigms have been proposed, evolving from the early days when folding was thought to proceed deterministically, through a unique series of conformations for a protein at hand, to the free energy landscape view founded upon description of an inherently stochastic but biased process. The latter emerged from polymer statistical thermodynamics and built evidence that protein folding energy landscapes are funnel-like, narrower at the bottom, as the freedom of the protein to populate low-energy regions is gradually restricted [5,28,356]. While the energy landscape view has inspired many folding and structure prediction algorithms, in itself there is no suggestion of a mechanism that can be followed to efficiently fold proteins in silico.

Application of MD simulations to observe the rare transition of a protein from an unfolded state to a folded state have come a long way in both the size of the proteins that can be handled and the time scales that can be modeled. Hardware advances, improvements in force fields, coarse-grained models, multiscaling techniques, and novel enhanced sampling techniques for MD have been crucial to surpassing spatial and time scales. Atomistic MD simulations can now be afforded [357], with supercomputers such as ANTON allowing running folding simulations of proteins of 50–100 amino acids for milliseconds [358], and software such as GROMACS [359], NAMD [116], and AMBER [360] becoming more accessible and easy to use to many researchers. In the following we elect to highlight recent work that showcases the state of protein folding. We then proceed with an overview of complementary work in de novo structure prediction.

Protein folding. Some of the most striking advances in protein folding with atomistic, equilibrium MD simulations in the presence of water molecules have come from the Pande group, particularly through the Folding@Home project [148,361–364]. In 2005, van der Spoel and colleagues provided the first folding simulation that also predicted the native structure of a peptide based on the Gibbs energy landscape [365]. In 2010, Shaw and colleagues successfully modeled the folding of a 35-residue protein in explicit solvent [147]. Soon afterward, Lindorff-Larsen and colleagues in the Shaw group managed to fold 12 fast-folding proteins of length up to 80 amino acids and diverse native topologies with atomistic detail and in explicit solvent [105]. Some striking observations were made from analysis of the folding trajectories of these small proteins, which generated much discussion in the protein folding community [366]. In addition to matching folding rates measured in the wet laboratory, work in [105] demonstrated that the folding trajectories contained discrete transitions between native and unfolded states, in agreement with barrier-limited cooperative folding. Pathway heterogeneity was shown to be minimal for nine of the 12 proteins, with pathways sharing more than 60% of the native contacts. These results naturally suggested that the pathways observed in simulation were variations of a single underlying folding pathway.

The conclusions in [105] were also supported by wet-laboratory work in [367], which detected a limited set of pathways and only four intermediates for the folding of the calmodulin. Moreover, in [105] it was observed that long-range contacts locking in place the native fold formed early along, together with a significant amount of secondary structures and surface burial. This was confirmed in other folding simulations, as well [368]. While the amount of residual structure is questioned by wet-laboratory studies and may possibly be the result of the bias of current force fields [366], the observations in [105] build the case for sequential stabilization as a mechanism for the folding of small, fast-folding proteins. The term sequential stabilization, coined in [369], refers to the fact that folding may not be completely cooperative but is characterized by small-scale events that add secondary structure elements named foldons [370] in a stepwise manner. Because foldons are intrinsically unstable, low-energy paths are likely to involve foldons building on top of existing structures, thus resulting in sequential stabilization.

Demonstration of the contribution and role of long-range native contacts early on in folding provided further justification for the use of Gō-models and other coarse-grained models that assume native contacts are the only ones that are kinetically-relevant [143]. However, while the wet-laboratory study of the folding of calmodulin in [367] demonstrated the presence of non-native intermediates in larger, more complex proteins, which is certainly observed in de novo structure prediction algorithms in the richness of non-native local minima. It is worth noting that a growing body of wet-laboratory studies are adding to the list of proteins known to fold through distinct native-like intermediates [371].

From a methodological point of view, a significant body of recent work in protein folding employs long, equilibrium, atomistic MD simulations in explicit solvent to observe multiple, spontaneous folding and unfolding events and reliably measure thermodynamic and kinetic quantities, such as folding rates, free energies, folding enthalpies, heat capacities, *ϕ*-values, and temperature-jump relaxation profiles [104,105,368]. While generally short, off-equilibrium MD simulations can at best sufficiently capture a single folding event, recent work that embeds many short off-equilibrium runs in coarse-grained kinetic models, such as MSMs, is able to approximate well the underlying folding dynamics [123,133,372]. Methods that embed many short simulations (MD or other stochastic optimization methods) in MSMs for the calculation of system dynamics is gaining ground in diverse applications, from folding, to structural transitions, to binding [128,132,354,373–375].

#### De novo protein structure prediction

The de novo structure prediction problem is perhaps one of the most popular and recognized ones in computational biology. The goal is to compute a structure that is representative of the protein native state given the amino-acid sequence of a protein with no known sequence homologs. This problem sprung from Anfinsen’s findings that the amino-acid sequence determines to a great extent the native state of a protein [11]. Knowing the native structure of a protein is central to protein-ligand binding studies, particularly in the context of CADD. The significant technological advances that have made high-throughput sequencing possible have also resulted in 1,000-fold more sequences than structures known for proteins.

Advances in in silico structure prediction can be attributed to Moult and colleagues, who founded the important Critical Assessment of protein Structure Prediction (CASP) competition to spur research in the structure prediction community in a competitive setting. At CASP gatherings, structures resolved in the wet laboratory and withheld from computational competitors are later revealed and compared with predictions. Community evaluations are then published and serve as a good measure of the progress in structure prediction. For instance, the latest review of structure prediction methods in [376] demonstrates that overall performance in CASP 10 improved substantially compared to previous competitions.

An exponential growth in the number of structures solved in the wet laboratory has had a dramatic effect on the utility of comparative modeling methods, which model structures of a target protein sequence after known structures/templates of proteins with similar sequences to the target; homologous structures can now be detected for most proteins [376]. HHPred is one of the most successful template-based predictors in CASP [377]. Nevertheless, de novo (or template-free, free, ab initio) modeling remains of great interest. Techniques used in de novo algorithms to model conformations of variable regions, such as loops, are also employed in template-based methods to fill in incomplete models [378]. Second, the goal of obtaining information on the equilibrium structure(s) of a protein from its amino-acid sequence is key to understanding function and changes to function upon perturbations.

Currently, state-of-the-art methods for de novo structure prediction rely on usage of the fragment replacement technique also known as fragment assembly. The technique allows simplifying and discretizing the conformation space explored by algorithms by essentially modifying a bundle of consecutive parameters, typically backbone angles of consecutive amino acids, simultaneously, as opposed to modifying individual backbone angles separately. A stretch of consecutive backbone angles is known as a fragment, and any protein conformation can yield a new one if a fragment can be selected in it and its configuration replaced with a new one. Originally introduced by Baker [379], the new configurations were obtained from a pre-compiled library configurations built over known protein structures in the PDB. Essentially, known protein structures are excised in consecutive overlapping fragments, and their configurations are recorded in a library indexed by the amino-acid sequence of a fragment. Replacement of fragment configurations naturally makes for a move or step in the context of an MC search, and most methods that use fragment replacement essentially implement enhanced sampling algorithms over baseline MC. For instance, the most recognized de novo structure prediction method, Rosetta [118], implements a multiscale MC method, which carefully switches from coarse-grained to atomistic representations in the growing MC trajectory, employing specifically-designed energy functions and even switching between two effective temperatures to cross energy barriers and so allow the MC search escape shallow local minima.

It is worth noting that careful construction of energy functions and representations of various granularity can be credited as much as the fragment replacement technique with advances in de novo structure prediction [119]. However, at the moment, a saturation point has been reached [380], and current research is focusing either on specialized moves for MC-based methods or other, higher-level mechanisms by which to enhance MC sampling. In current top CASP performers, secondary structures are built and packed relatively easily, and the difficulty in correct predictions is localized to variable regions such as loops. For this reason, efforts are devoted to rethinking the moves in an MC-based setting beyond fragment replacement.

Work in [119,120], which has resulted in the highly-successful Quark method, shows the utility of designing different types of moves and employing them at various stages during the MC search. As reported inn [120], Quark performs very well in the free modeling category. Performacne on 34 free modeling targets is measured by calculating the TM-score between the best prediction and the known native structure for each target versus target length (TM-score is a metric for measuring structural similarity and is considered superior to RMSD [381]; the reader is directed to Ref. [382] for details.). Performance is unusually high (>0.5) for targets (R0006-D1, R0007-D1, and R0012-D1) that are longer than 150 amino acids. In particular, two of the targets, R0006-D1, R0007-D1, were considered difficult targets in the CASP10-ROLL experiment. On R0006-D1, which is a *β*-barrel protein 169 amino acids long, Quark generates five models with the highest TM-score of 0.32. Structural superposition extracts a model with TM-score 0.5, which improves to a TM-score of 0.622 after energetic refinement via I-TASSER [383]. On R0007-D1, which is an *α* protein 161 amino acids long, Quark generates a best model with TM-score 0.43. Structural superposition extracts a model with TM-score 0.48 from the LOMETS template pool, which then improves to a TM-score of 0.62 after energetic refinement via I-TASSER. These results suggest that the focus on designing specialized moves is well placed.

Other work is focusing on enhancing the sampling capability beyond a simple MC-based search or even an MC-SA, though there is a growing consensus that improving accuracy in scoring functions may be more important than enhancing sampling to advance the state of *de-novo* structure prediction. Progress in enhancing sampling comes from different communities of computational biologists and computer scientists. One direction focuses on gradually narrowing the search space, either by iteratively fixing segments of the chain exhibiting low diversity among sampled low-energy conformations [384] or indirectly achieving the same effect but by changing the probability distribution function over the fragment configuration library [385]. Other work builds on model-based search and uses information gathered during the search to guide exploration towards promising regions of the conformation space [386,387]. In [386] gathered information is used to identify near-optimal minima worth exploring in greater detail with all-atom energy functions. In [387], a robotics-inspired algorithm adapts the search towards under-sampled but low-energy regions of the conformation space to balance breadth versus depth.

The issue of how to balance computational resources between exploring the breadth of conformational space while going deep down in local minima is a core one in stochastic optimization. Progress has been made over the years, particularly by evolutionary algorithms that are now competitive with MC-based methods such as Rosetta [388–390]. Pursuing evolutionary algorithms for conformation sampling in de novo structure prediction has opened up novel directions on the design of effective moves [391] and multi-objective optimization [392], where the goal is not to minimize an aggregate energy score but instead improve on several orthogonal categories.

Currently, de novo structure prediction methods are focused on proteins with one well-defined native structure. Multi-basin proteins present a challenge, as they demand much more computational resources be spent on exploring the breadth of the energy landscape. In addition, conformation sampling (also known as decoy sampling) is not the only challenge with de novo structure prediction. Analysis of sampled conformations to identify the native structure and offer it as prediction presents its own challenges. This problem in itself is known as decoy selection, and a review of challenges and the state of the art is presented in [393].

### Modeling Structure and Dynamics of Intrinsically-Disordered Proteins and Intrinsically-Disordered Protein Regions

Lately, increasing attention is paid to the problem of characterizing the structure and dynamics of intrinsically-disordered proteins (IDPs) [394–396]. There are now growing databases of IDPS and intrinsically-disordered protein regions (IDPRs), such as pE-DB, DisProt and IDEAL [397–399]. CECAM now regularly includes a workshop dedicated to promoting the development of new modeling methods and better understanding IDPs [400]. Since 2002, even CASP provides an independent assessment of methods for IDPS [396]. Several reviews discuss the fundamental principles of disorder in the biological function of IDPs/IDPRSs biological functions, including the role of disorder in cancer, neurodegeneration, genetic forms of Parkinson’s disease, and cardiovascular diseases [401–405].

IDPs/IDPRs pose unique challenges in silico. They do not have stable tertiary structures but still demonstrate biological activity. This phenomenon challenges the fundamental structure-function relationship and is an extreme case of the exception to the lock-and-key model [395]. IDPs/IDPRs are not random coils. They exhibit different degrees of disorder, from molten globules to coils, but even coil-like structures exhibit residual structure [402,405]. A recent replica exchange MD simulation study revealed the structural contents of intrinsically disordered tau proteins. Tau proteins were discovered to be able to catalyze self-acetylation, which may promote pathological aggregation. The work characterized the atomic structures of two truncated tau constructs, K18 and K19, providing structural insights into tau’s paradox [406].

IDPRs sequences are very different from those of ordered proteins, poor in hydrophobic amino acids and rich in charged amino aids. Disorder-promoting amino acids have now been identified, and they include Ala, Arg, Gly, Gln, Ser, Glu, Lys, and Pro [404,405]. Based on sequence information alone, tools now exist to estimate the propensity of a sequence for disorder [407]. There are many methods for disorder analysis and prediction of the location of disordered regions [124,408–411].

Computational methods are being designed to characterize structures and dynamics of IDPs/IDPRs. With specifically designed force fields, some methods have shown promise in this regard [412,413]. Treatment of IDPRs is now included in Rosetta [414]. Two main groups of methods focus on IDPs/IDPRs. The first group consists of wet-laboratory techniques based on NMR Chemical Shifts and RDCs [415]. The second consists of MD-based methods [152,153,408,416–418].

Both unrestrained MD [416] and long-range correlated MD [417] for well-characterized disordered proteins demonstrate good agreement with wet-laboratory data. The replica exchange with guided annealing method has also been shown suitable for IDPs [418]. The method escapes nonspecific compact states more efficiently and speeds up the generation of correct ensembles compared to classic replica exchange simulations. Work in [153] additionally shows the effectiveness of MD and MSMs for IDP modeling.

Other methods combine NMR-based knowledge and MD simulations [6,302,314,413]. While NMR ensembles are better suited to characterize local conformational states of IDPs [415], MD simulations allow calculating kinetics and elucidating meta-stable states and barriers between states [314]. Given their unique characteristics, computational methods are expected to continue their treatment of IDPS to better understand the connection between disorder and biological function and misfunction.

### Protein Design

The protein design problem is that of finding an amino-acid sequence whose global free energy minimum state corresponds to a desired, target structure or contains a structural motif associated with a desired function [419]. Also known as inverse folding or inverse structure prediction, this problem is now at the crux of protein engineering, with applications in medicine, biotechnology, synthetic biology, nanotechnology, biomimetics, and more [420]. Stated as an optimization problem, protein design is amenable to algorithmic frameworks employed for structure prediction.

Computational approaches to protein design can be categorized into forward design, explicit negative design, and heuristic negative design [419]. In forward design, the sequence and target fold/structure of a protein are known, and the goal is to optimize the sequence so that the target structure reaches such a low energy that will make any other non-target structures less energetically favored. No explicit non-target structures are considered. A successful application of forward design has yielded a very stable protein, Top7 [421], whose native structure was later shown identical to the determined X-ray structure. In explicit negative design, alternative structures are explicitly considered. The sequence is optimized so that the target native structure is lower in energy than all the alternative structures. Explicit negative design has been used to design specific coiled coils and DNA-binding and -cleaving enzymes [422–425].

The limitation of explicit negative design regarding prior knowledge and enumeration of non-favored alternative states has motivated heuristic negative design. In heuristic negative design, the goal is not to disfavor specific alternative structures; instead, the sequence is optimized through features that are likely to increase the energy of most undesired structures. Features follow closely strategies employed by nature to achieve the energy gap between the native structure and other structures that seems to be required for thermodynamic stability and function [419]. It is worth noting that conclusions regarding energy gaps between native and non-native structures when employing scoring functions need to be taken with a grain of salt. Work in [365] relates gaps in Gibbs free energy to structure deviations (from NMR data).

Compared to the other two strategies summarized above, heuristic negative design seems particularly important for biomolecular interactions [426,427]. Heuristic negative design also seems to be employed by nature for IDPs and by pathogens to fend off the host immune system [419].

Successful cases of designing proteins with novel functions abound [428–430] and are made possible by considerable advances in methods for de novo protein design. The current predominant computational approach is based on the (inverse folding) paradigm proposed in [431], which assumes a fixed backbone and searches over discrete low-energy configurations/rotamers of side chains for rotameric combinations that result in a lowest-energy all-atom tertiary structure [432]. In the interest of tractability, energy models are limited to pairwise energy functions. State-of-the-art functions for protein design are knowledge-based, relying on statistical parameters derived from databases of known protein properties [433–437]. Even with such energy models, the design problem with a rigid backbone and a discrete set of rotamers has been proven to be NP-hard [438].

Two types of methods have been proposed to address the combinatorial optimization problem of finding rotameric combinations. The first are based on exact optimization and seek completeness; that is, finding the global minimum energy conformation. The second forego completeness and are based on heuristic optimization.

**Fig 4:**
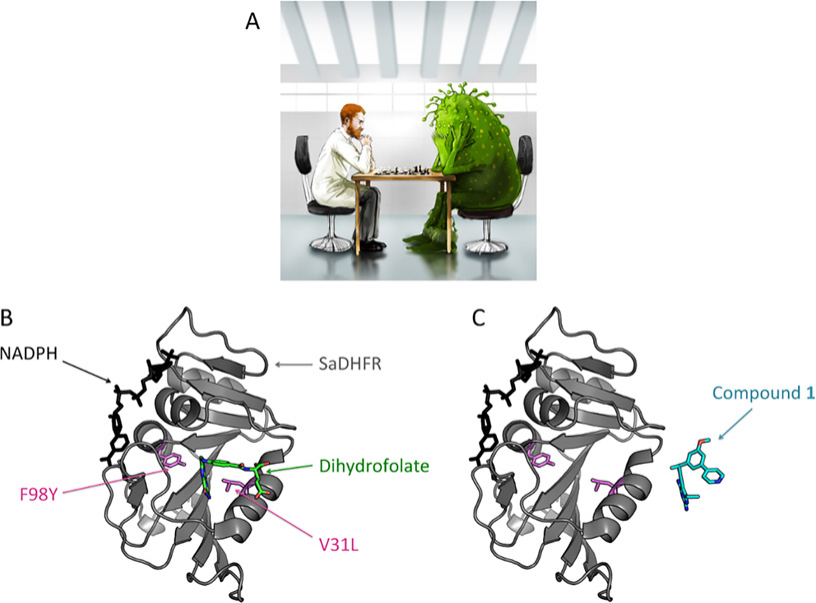
Predicting a pathogen’s resistance mutations [452]. (A) Pictured is an illustration of a game between scientists and bacteria. For every drug that scientists develop against bacteria (a “move”), bacteria respond with mutations that confer resistance to the drug. This paper shows that these “moves” by bacteria can be predicted in silico ahead of time by the Osprey protein design algorithm. Donald, Anderson, and coworkers used Osprey to prospectively predict in silico mutations in Staphylococcus aureus against a novel preclinical antibiotic, and validated their predictions in vitro and in resistance selection experiments. Image (A) was created for this paper by Lei Chen and Yan Liang (L2Molecule.com). (B–C) Computationally predicting drug resistance mutations early in the discovery phase would be an important breakthrough in drug development. The most meaningful predictions of target mutations will show reduced affinity for the drug (C) while maintaining viability in the complex context of a cell (B). The protein design algorithm, K* in Osprey, was used to predict a single nucleotide polymorphism in the target DHFR that confers resistance to an experimental antifolate (Compound 1) in the preclinical discovery phase. Excitingly, the mutation was also selected in bacteria under antifolate pressure, confirming the prediction of a viable molecular response to external stress. Images (B–C) were created by Adegoke Ojewole in the Bruce Donald Lab, Duke University.

Exact optimization methods include dead-end elimination [439], branch-and-bound algorithms [440–442], integer linear programming [443,444], dynamic programming [445], or cost function networks [446]. These exact methods are efficient and they limit inaccuracies to the inadequacy of the energy model, but their focus on one single assignment is highly subjective to possible artifacts in the energy function, known and lamented in [447]. Moreover, the solution provided by such methods may be overly stabilized (effectively residing in a narrow basin), that it lacks the structural flexibility for the protein to operate the sought biological function under physiological conditions [448]. It is worth noting that unlike discrete rotamer assignments, work by Donald and colleagues pursues continuous rotamers and is able to reach lower-energy conformations [449]. This functionality is integrated in the popular OSPREY software [450]. It is expected that the design of a smoothed backbone-dependent rotamer library in [451], which allows evaluating rotamer characteristics as smooth and continuous functions of the *ϕ*, *ψ* angles will lead to more advances in taking into account side-chain flexibility in de novo design. An illustration of the capability of protein design algorithms is provided in Fig 4.

Heuristic optimization methods for de novo design build on stochastic optimization or meta-heuristics, such as MC-SA [433,453], Genetic Algorithms [454,455], and other stochastic optimization methods [442,456,457]. Methods based on stochastic optimization, best represented by RosettaDesign [458], currently dominate, mainly due to the ability to provide an ensemble of near-optimal solutions through their sampling-based approach. The backbone is kept fixed, and rotameric states are sampled systematically or in a sampling-based manner [433,453] over pre-built rotamer libraries [435,459]. All-atom energy minimization of the entire resulting all-atom conformation is often carried out [460,461]. It is here, in the minimization stage to which all constructed conformations and sequences are subjected, that localized backbone fluctuations are allowed. The extent of these fluctuations is small, limited to backrub motions [462–465]. Larger motions are allowed, but only on loop regions, made possible by efficient inverse kinematics techniques like Cyclic Coordinate Descent [320,466].

The importance of allowing backbone flexibility in the design process cannot be overestimated. The simple model of the backrub motion consists of a small dipeptide rotation about the C-C*α* axis. Recent studies suggest that integrating backrub motions in the design process leads to improved designs of protein-protein interaction interfaces and more realistic templates with improved fit between simulated side-chain dynamics and NMR data [462,464, 467]. Additionally, work in [468] has demonstrated that taking into account backrub motions expands sequence diversity during search and allows new residue interactions that rigid-backbone approaches cannot accommodate. This leads to better designs with lower energies and has been confirmed in other studies, as well [469,470].

Finally, an important highlight in protein design is the fact that, despite the absence of evolutionary history in newly-designed proteins, evolutionary information can be accommodated in the design process. Work in [470] reveals strong correlations between residue covariance in naturally-occurring protein sequences and sequences optimized for the same structures by computational protein design. Covariance has been demonstrated for complementary changes in residue size, residue charge, and hydrogen bonding [471–475]. These findings suggest that structural restrains on co-evolving residues in contact can lead to further improvements both in de novo protein design and structure prediction.

## Categorization by Algorithmic Frameworks

In the following, we categorize methods by the algorithmic frameworks they modify and adapt for investigating macromolecular structure and dynamics.

### MD-Based Methods and Enhancements

In the classic MD setting, Newton’s equation of motion is iteratively solved on a finely discretized time scale to observe collective movements of the atoms comprising a molecular system through successive conformations terminating at a local minimum conformation in the system’s energy surface. The ensemble of conformations obtained at equilibrium conditions observes the Boltzmann distribution. A distinct advantage of employing MD to simulate the equilibrium dynamics of a macromolecule is the ability to obtain great detail on individual and correlated motions of specific atoms and specific sites on a macromolecule, as well as correlated motions between macromolecular units of a complex. A disadvantage of the classic MD simulation setting is the inability to sample rare events that occur on long time scales. In particular, in the presence of high energetic barriers separating local minima in the energy surface, a classic MD simulation may be trapped and never escape within the time scale of the simulation.

Limited sampling of the conformation space is a fundamental issue in classical MD, and algorithmic enhancements are proposed on a regular basis to enhance sampling capability. These include replica exchange, accelerated MD, umbrella sampling, biased or steered MD, importance sampling, activation relaxation, local elevation, conformational flooding, jump walking, multicanonical ensemble, MSM-driven MD, discrete timestep MD, swarm methods, and others [8,149,203–206,334,476–489].

Recent reviews of advanced MD-based methods and outstanding issues are discussed in [124,490–495]. A comprehensive list of commonly used MD packages for biomolecular simulation is presented in [493]. Examples of MD applications on proteins with large conformational changes that occur on long time scales, such as G-proteins, Ras-proteins, kinases, signaling proteins, and others can be found in [121,122]. In the following, we highlight some of the algorithmic enhancements to the classical MD setting that are responsible for surpassing traditional MD time scales and characterizing the dynamics of complex systems.

#### Accelerated MD and adaptations

The accelerated MD method [496,497] locally flattens the potential energy surface to decrease the free energy barriers between two conformational states. When the system’s potential energy falls below some predefined threshold energy E, a bias potential is added. The level of flattening is regulated by two parameters that are typically specified by the user: the threshold energy E, which controls the portion of the potential surface affected by the bias, and the acceleration factor *α*, which determines the shape of the bias potential and thus how flattened the energy surface becomes. The bias potential allows escaping deep minima separated by high energy barriers, thus accelerating the transition between two conformational states of interest and extending the time scale of events that can be observed in simulation. Recent accelerated MD simulations with nanosecond steps [498] can explore more conformational dynamic events [499,500]. However, Boltzmann statistics need to be recovered from the simulations, and the effect of the bias potential must be unwinded. A reweighting procedure is typically used, which attempts to convert an accelerated MD trajectory to the canonical ensemble at a given temperature [8,501].

Enhancements and adaptations of the baseline accelerated MD method are being proposed. We note here first the self-learning, reconnaissance metadynamics method [502], which combines principles of accelerated MD and the concept of collective variables that is the foundation of the metadynamics strategy. Similar to the baseline method, a bias potential is added to the true potential to locally flatten the energy surface. However, the bias potential is constructed over the low free energy region defined over a large number of locally-valid collective variables. The accelerated adaptive integration method [203] can be considered another adaptation of the baseline accelerated MD method for the problem of modeling ligand-binding processes. A ligand coupling parameter *λ* is introduced to keep track of the end points of the receptor-ligand coupling and decoupling process; *λ* takes values from 0 to 1. The method assumes that some transitions can be more accessible if a certain stage of coupling/decoupling (*λ*) is reached; the potential energy function is flattened at intermediate values of *λ* instead of at some threshold energy value E.

#### Replica exchange MD methods

Replica exchange is a popular enhancement of the classical MD method; it is also known as parallel tempering. Originally, replica exchange was introduced to improve properties of the MC framework [503], but has since then been adapted to enhance MD sampling [504]. The usual continuous MD trajectory is broken into several replica simulations randomly initialized and conducted at different temperatures. The number of replica simulations is typically determined by the user. So is the decision on temperatures assigned to the replica simulations. The simulations exchange information with one another by exchanging conformations at regular intervals. At a time, two simulations are selected, and their instantaneous conformations are exchanged according to the Metropolis criterion. The exchange often allows a particular simulation to escape a local minimum by making conformations accessed at higher temperatures available to those at lower temperatures, thus enhancing sampling capability. In addition, the setting of multiple simulations encourages parallel implementation and employment of distributed architectures with message passing. This gives replica exchange high exploration capability. Many adaptations and applications of replica exchange exist [149,478,505]. Work in [506] proposes a technique to deduce kinetics data from a heterogeneous ensemble of simulation trajectories. A detailed review of methods based on replica exchange can be found in [478].

#### Restrained ensemble MD methods

We note here two methods to illustrate the employment of experimental data as restraints in MD-based simulations, the replica-averaged MD method and the replica-averaged metadynamics method. The employment of experimental data to correct a molecular force field and thus steer the sampled conformation ensemble towards the Boltzmann distribution has a rich history in macromolecular modeling. The idea of using experimental measurements as averaged structural restraints in MD simulations was first implemented for distances derived from NOE [35]. A penalty term was added to the force field if the time-average of an NMR observable calculated from an MD trajectory differed from that provided by experiment. A variation of this idea is to measure not a time-average but an ensemble-average observable. The latter is referred to as the replica-averaged approach, and a variety of restraining algorithms, including those that conduct both time and ensemble averaging, have been developed and applied to sample and characterize native, transition, intermediate, and unfolded states of proteins [17,32,34,312,316,507–512].

Vendruscolo and colleagues [304] have demonstrated that MD simulations with replica-averaged structural restraints allow generating structural ensembles according to the maximum entropy principle introduced by Jaynes [513]. Jaynes addressed the problem of incorporating information from experiments into a structural model while avoiding corrupting the model with spurious and arbitrary biases. His maximum entropy method, however, proved too cumbersome. The restrained ensemble methods of Vendruscolo and others provide an alternative practical approach, but, until recently, it was not known whether these methods obey the maximum entropy principle. In addition to work in [304], Roux and collaborators demonstrate in [514] that restrained-ensemble MD simulations produce statistical distributions that are formally consistent with the maximum entropy principle.

Distance restraints from NOE data, if available, can be integrated in ALMOST, an all-atom molecular simulation open-source package for macromolecules structure determination and analysis [515]. In the replica-averaged metadynamics method [516], in addition to making use of replica-averaged restraints in the force field, the metadynamics framework is exploited to enhance sampling. Application on the *α*-conotoxin SI, a 13-residue peptide that has been characterized extensively in the wet laboratory, shows that the method enables accurate reconstruction of the free energy landscape.

#### Umbrella sampling

Umbrella sampling [517–519] is another method that employs collective variables. Umbrella sampling is related to importance sampling in statistics. Umbrella sampling addresses systems with energy landscapes where a high energy barrier separates two regions of the conformation space. The relevant system coordinates are grouped into sets of collective variables, with each set determining a separate umbrella window. A restraint bias potential forces the collective variables in a window to remain close to the center of mass. The restraint potential often takes a quadratic or harmonic form, determining the weighting function of a given window. If the configurations in a window are far from the equilibrium state, the weighting function will be large, and the simulation will be biased away from the initial configuration. The sets of collective variables must allow for slight overlap of their windows for proper reconstruction of the transitions between them. Extracting corresponding Boltzmann averages and handling overlapping weighting functions are key issues. The information from each window-biased simulation is converted into local probability histograms. The weighted histogram analysis method (WHAM) [520] is now the standard method to combine results from a set of umbrella sampling simulations. Work in [521] introduces superlinear numerical optimization algorithms to diagnose and quantify systematic errors due to limited sampling and to obtain fast and accurate solutions of coupled nonlinear WHAM equations. Work in [522] introduces a bootstrap method to accurately estimate error due to insufficient sampling and incorporates autocorrelations to reduce such errors. The method, g_wham, has been incorporated in the popular GROMACS molecules simulation suite [359]. The umbrella sampling scheme can be integrated into other enhanced MD or MC strategies. We highlight here the self-learning umbrella sampling method in [523], which learns, through a feedback mechanism, which regions of a multidimensional space are worth exploring and automatically generates a set of windows. This method needs a significant smaller number of umbrella windows to characterize the free energy landscape over the most relevant regions without any loss in accuracy. Umbrella sampling has been employed to study processes with large conformational changes or rare events, such as ligand binding and ion induced diffusion in membrane proteins [523,524].

#### Adaptive MD sampling methods

Guiding MD sampling via on-the-fly analysis of obtained conformations to determine undersampled regions of the conformation space is gaining ground in macromolecular modeling. The principal difficulty with adaptive sampling is the identification of meaningful collective variables over which to project conformations and obtain lower-dimensional embeddings of the conformation space for the identification of under-sampled regions and calculation of interesting statistics. While collective variables, such as number of native and non-native contacts, hydrogen bonds, dihedral angles, RMSD, radius of gyration remain popular, these variables have been shown to result in overly smooth landscapes [525] and mask interesting transitions. Recent work by Clementi and colleagues has reintroduced diffusion-based dimensionality reduction methods for extracting collective variables and has demonstrated the power of such methods for characterizing complex energy landscapes [526,527]. Further work by the same authors in [528,529] employs the identified collective variables to guide and expedite sampling of rare events via MD.

In contrast to methods that rely on the identification of collective variables, a different line of work in the early 2000s introduced the concept of kinetic clustering and conformation space network. Both were precursors of the MSM. The main idea was to organize conformations in discrete, graph-based models of connectivity to both visualize the free energy surface and carry out interesting calculations on such models.

The concept of kinetic clustering evolved from the disconnectivity graphs put forth separately by Karplus and Wales [530–532]. Work by Rao and Caflisch took this idea further by proposing complex network analysis both to visualize and study the conformation space and folding of peptides [533]. In lieu of geometric clustering, conformations in [533] were grouped together by secondary structure, and the different emerging groups were abstracted as nodes of a network, with links between nodes recording observed transitions between groups. Interesting observations were made regarding network topology and peptide folding kinetics in [533] and in later applications investigating the impact of single-point mutations on peptide folding [534] (a detailed review of the conformation network idea can be found in [535]), but the broader analogy (and generalization) between conformation space networks and MSMs would emerge later. In tandem with the conformation space network proposed by Caflisch, related work by Karplus further propelled the disconnectivity graphs to additionally employ max-flow/min-cut algorithms to lay bare the hidden complexity of free energy surfaces of peptides and proteins [525,536]. It is worth noting in this context that the free energy surface generated by implicit solvent is often very different and more complex than that generated by explicit solvent [537]. Early work in [538] demonstrates that explicit solvent smooths the energy surface.

Kinetic clustering continues to be useful and has been used successfully to characterize protein folding through very long MD simulations [147]. In [147], conformations are assigned to clusters so that the long time scale behavior in cluster-space mimics that in the MD simulation. Autocorrelation functions of the time series of a large number of atomic distances are calculated to match the long time scale of these functions with corresponding correlation functions calculated over dynamics in cluster space. The assignments and then the construction of transitions between distinct long-lived states identifies the slower transitions [147].

It was only around 2005 that the analogy between the conformation space network and the MSM was made by Pande and coworkers [363,539]. The notion of kinetic clustering was generalized, and the conformation space networks evolved into kinetic networks connecting meta-stable states, effectively MSMs [540]. The integration of MSMs [146,153,541] into MD simulations allows investigating macromolecular dynamics even beyond the second time scale [123]. Originally, MSMs were only employed to analyze the connectivity of conformational states sampled through multiple, long MD simulations and employ calculations over the MSM to derive kinetic measurements [363]. In [123], MSMs were employed to reconstruct folding pathways from short off-equilibrium, all-atom simulations in explicit solvent. MSM and MD methods have been applied to model folding [542–545], protein-ligand binding [136,138,546], protein switches in kinase and GPCRs [547,548], allostery [549] and IDPs [541,550], revealing extensive statistical details about intermediates states [136,542,551] and molecular interaction mechanisms. The employment of MSMs to focus computational resources to under-sampled regions of the conformation space in an adaptive manner is a rather recent development in macromolecular modeling. A semi-automatic protocol has been proposed in [552] to simulate the folding and unfolding of the villin headpiece in a very efficient manner. Work in [128] also proposes a semi-automatic protocol analyzing MD trajectories with a constructed MSM model to pinpoint where more sampling needs to be conducted. As of now, a fully automatic protocol remains elusive [553].

While MSM-guided MD sampling relies on obtaining a discrete model of the connectivity of the sampled conformation space to guide further sampling, other methods rely on modifying the energy function itself to bias the simulation away from already-sampled conformations. One of the earliest methods to do so was local elevation [481]. In local elevation, the actual potential energy surface is modified in order to drive conformational sampling away from visited conformations (a bias term that is the sum of of repulsive functions is added to the potential energy function).

Metadynamics methods follow a similar approach [554,555]. The assumption in these methods is that the system can be described in terms of a few collective variables. During the MD simulation, the location of the system is calculated in terms of the collective variables. A positive Gaussian potential is then added to the energy landscape so that the simulation is biased to return to the previous location. During the simulation, more and more Gaussians add up to the point that the system is discouraged from going back to previous locations in the energy landscape, thus exploring the full landscape. The time interval between the addition of two Gaussians and the height and width of a Gaussian are all tunable parameters to optimize the ratio between accuracy and computational cost. The crucial issue in metadynamics, as in other techniques based on collective variables, is to identify the right collective variables. Strategies to do so are reviewed in [555]. The metadynamics strategy is available as a portable plugin for MD simulation platforms in PLUMED [556]. Metadynamics MD has been applied to study the folding process of small proteins [557,558], protein switches [559–561], and ion induced diffusion of small molecules in cavities and channels [562,563]. Metadynamics methods have also allowed modeling the docking process with full protein flexibility [135,564–567].

### MC-Based Methods and Enhancements

While a significant portion of research on macromolecular structure and dynamics employs MD-based methods, a just as significant portion employs MC sampling. In MC, the evolution of one conformation into another is not guided by Newton’s equation of motion but instead a programmed move or step designed to introduce a small or large conformational change. The end result of the move is only accepted according to the Metropolis criterion in order to promote the trajectory of consecutive conformations to converge to the global minimum while allowing some non-zero probability of escaping a current minimum. MC-based methods employ the notion of effective temperature to regulate the height of energy barriers that can be crossed. While generally regarded to have higher sampling capability than MD, MC methods also are prone to convergence to local minima and forego any direct information of time scales and kinetics. Many of the enhancement strategies for MD can be applied to MC-based methods. In the following we highlight two such enhancements.

#### Collective motions molecular dynamics and Monte Carlo

Collective MD [568] belongs to the family of enhanced MD sampling methods that simplify sampling considering only the most dominant, low-frequency, low-resolution, collective motions. The latter are identified by modeling a structure through the anisotropic network model (ANM) [569]. The basic approach is to deform the structure collectively along the modes predicted by the ANM. A Metropolis-based MC scheme is employed to select the ANM modes; the stochasticity permits the system to occasionally circumvent energy barriers. The ANMPathway is a related sampling method that uses modes extracted from two ENMs representative of the experimental structures that constitute the end points of the transition under investigation [570]. Both methods have been tested on modeling open-close transitions in AdK [568,570] and several transporting membrane proteins [570]; the transition pathways were captured in great detail and at significantly lower computational cost than other methods [571].

#### Weighted ensemble method

The weighted ensemble method (WEM) [572] is an enhanced sampling method with simplified sampling. WEM uses a multiple-trajectory strategy in which individual trajectories can spawn multiple daughter trajectories upon reaching new regions of configuration space called bins. The daughters are suitably weighted to ensure statistical rigor. WEM can yield rigorous estimates for time scales that are much longer than the simulations themselves. The idea to split and propagate re-weighted trajectories had been initially introduced in MC simulations, but WEM can be used as a sampling method for MD simulations, as well [572]. WEM has been employed to model folding [573], non-equilibrium [574] and equilibrium and processes [572], and conformational transitions between end-points separated by high energy barriers [575].

### Other Algorithmic Frameworks

#### Morphing methods

Geometric morphing uses the linear interpolation of each atom to construct a path between conformations. MolMovDB [337,576] was the first online tool to allow obtaining and visualizing such paths. After each linear interpolation, the morphing algorithm in MolMovDB conducts an energy minimization to fix possible distortions and restore the stereochemistry of the intermediate points in the interpolated trajectory. The created morphs are stored in the database of motions and can be found by protein name, PDB ID, or motion type [577].

Conformational trajectories based on linear interpolation do not necessarily represent actual conformational pathways. Several morphing-based methods have been developed that provide non-linear interpolations between the start and goal structures to be connected through intermediate conformations [130,338,341,578–580]. Non-linear morphing methods rely on normal mode analysis (NMA) of harmonic-type models, such as the ENM and its variants, to obtain principle motions of a macromolecule about a local minimum. Such models are based on early concepts by Go, Scheraga, and Flory [581–583], and they rely on the assumption that macromolecules can be treated as deformable elastic bodies, where the interatomic potential function can be represented by a harmonic model [584,585], and interactions depend only on the density of neighbors [586,587]. The earliest application of NMA to elucidate equilibrium dynamics was conducted in the Karplus laboratory [228], though the usage of normal modes predates this by seven years; Levitt and Warshel used normal modes to jump out of local minima in pioneering folding simulations [68,72]. Further work demonstrated the effectiveness of such models for capturing thermal vibrations and predicting experimental B-factors [584,585,588–590]. Other work employed normal modes extracted via NMA from a single structure to model equilibrium fluctuations and in some cases even capture simple conformational switching [591–598]. The NOMAD-Ref server [339] provides tools for online NMA of large molecules (of up to 100,000 atoms, maintaining atomistic detail of their structures) and access to a number of programs that use the normal modes to model deformations and conduct refinements of experimental structures.

The earliest employment of NMA in the non-linear morphing setting, to extract information on intermediate conformations mediating the transition between a goal and start structure, appeared in [341,599]. In [599], a geometric morphing technique is proposed to bridge two ENMs corresponding to given start and goal structures. Related ideas appeared in [600,601], moving along a few normal modes from the start structure pointing to the target structure and then parameterizing the elastic network along the pathway. In [578], the start and goal structures are interpolated upon optimal superposition of the CA atoms, but, in contrast to linear morphing methods, the resulting displacement vector is expanded as a linear combination of the normal modes calculated on the start structure.

Since, typically ENMs involve only a single energy minimum and are not immediately applicable to model transitions between multiple stable and semi-stable structural states of a macromolecule, mixed ENMs [579,602] and other, related, ENM-based models have been developed [130,603–606]. The fundamental issue addressed in different ways in these works is how to interpolate the ENMs at the start and goal structures so that the resulting potential retains these structures as local minima [602]. The plastic network model (PNM) introduced in [603] can include additional known intermediate structures and is parameterized to account for known fluctuations available as experimental B-factors.

A group of non-linear morphing methods based on ENMs, mixed ENMs, and variants such as PNM, compute transitions that are minimum-energy paths (MEP) in the energy landscape. In [603], the conjugate peak refinement (CPR) algorithm [607] is used to compute a series of steepest descent paths from saddle points to nearest minima to connect two structures of interest with a continuous curve in the conformation space. Similarly, in the Climber method [340,608], a restraining energy depends linearly on the distance deviation between the current conformation and the target conformation in a way that allows full flexibility and enables the protein to move around high-energy barriers, rather than over them, resulting in the MEP. KOSMOS [609] is another online morph server that, in addition to offering NMA for nucleic acids, proteins, and their complexes, also generates plausible transition pathways by optimizing a topology-oriented cost function that guarantees a smooth transition without steric clashes.

#### Transition path sampling and chain-of-states methods

The main challenge with computing transitions of a macromolecule between meta-stable states or basins is due to the fact that a macromolecule may spend a very long time in one basin before transitioning to another. The disparity between the effective thermal energy and the typical energy barrier is manifested in long waiting periods where the macromolecule diffuses in a basin followed by a sudden jump to another basin. Such sudden jumps are rare events, and a significant body of work in macromolecular modeling is dedicated to enhancing conventional MC or MD simulation frameworks to capture such events in a reasonable time frame. These methods operationalize seminal ideas put forth by Pratt on transition path sampling (TPS) [610]. Even though the energy landscape of a complex system is typically dense in saddle points, only a few saddle points are relevant for transitions between basins. TPS methods do not rely on identifying saddle points in the potential energy surface. Instead, they implement importance sampling over a reduced set of collective variables that span the important regions of the high-dimensional search space [611–616]. TPS methods are numerical techniques that effectively conduct MC sampling of the ensemble of transition paths [617]. Detailed reviews of these methods can be found in [617,618].

Transition paths obtained via TPS methods can be quite complicated for systems with high-dimensional conformation spaces and rugged energy landscapes; a statistical mechanics framework, known as the transition path theory (TPT) [619], is needed to organize and analyze the transition path ensemble. Moreover, the success of TPS methods depends on the particular progress coordinate defined to distinguish the transition path in the search space, but finding an effective coordinate is non-trivial. Indeed, multiple progress coordinates may need to be defined to describe the transition.

Therefore, a second group of methods founded on TPT implement the chain-of-states approach, which assumes that the transition path can be meaningfully encoded as a series or chain of structures (also referred to as images) [342,607,620–623]. These methods can track an arbitrary number of progress coordinates while restraining sampling to effectively one dimension. In chain-of-states methods, a string of images is created between the given meta-stable states, and the images are relaxed to the transition pathway. Similar ideas had already appeared in [607,620]. Two types of chain-of-states methods were proposed afterwards, the nudged elastic band (NEB) methods and the string methods.

The NEB method [624] addresses a key issue that arises when an artificial spring force is introduced to maintain even spacing between images. The problem is that when minimizing the elastic band, the component of the spring force that is perpendicular to the elastic band tends to pull the images off the MEP. To address this problem, in NEB, a minimization of the elastic band is carried out where the perpendicular component of the spring force and the parallel component of the true force are projected out. In this way, the spring force does not interfere with the relaxation of the images perpendicular to the path. The result is that the series of relaxed configurations is an approximation to the MEP, converging to the MEP when there is sufficient resolution in the discrete representation of the path (when enough images are included in the chain). It is worth noting that the MEP is just one, *special* path selected from curves connecting two given conformations. Work in [625] explains that this special path minimizes the absolute value of the mechanical work and so is the most probable path for an overdamped Brownian particle at 0 K [625] (in other words, the most probable Brownian trajectory in the absence of kinetic energy). Improvements to the NEB method introduced in [624] have been proposed, particularly regarding improving the tangent estimate [621] and lowering the computational cost of minimizations [342].

Generally, NEB methods require that the energy landscape be relatively smooth and are not effective on rugged energy landscapes [619]. Remedies have been proposed by having NEB methods operate on the free energy landscape [623], which is expected to be smoother, or by introducing temperature corrections to the MEP [626]. Caution must be exercised not to double count entropy when operating on free energy landscapes. One implication is that implicit solvent potentials cannot be employed to model dynamics on free energy landscapes.

In string methods, splines are used instead to calculate tangents. In addition, image spacing is maintained via reparameterization. The first string method proposed in [622] belongs to the sub-category of zero-temperature string methods [344]. Extensions to operate on the space of collective variables and compute the minimum free energy path (MFEP) rather than MEP have also been proposed [343,345]. Finite-temperature string methods were later proposed [347,627] to better deal with overly rugged energy landscapes.

String methods do not assume the energy landscape is smooth. They can also handle a large number of collective variables. Effective choices of collective variables have been discussed and tested in [628]. Work in [619] draws a difference between string methods and chain-of-states methods, as string methods start with an intrinsic formulation of the dynamics of curves/strings in configuration space and only resemble chain-of-states methods after discretization of the curves. String methods sample the configuration space with strings, which are smooth curves with intrinsic parameterization. The mean force and other conditional expectations are computed locally over the discretization points along the string. The string satisfies a differential equation that by construction guarantees that the string evolves to the most probable transition path connecting two meta-stable states.

In particular, the finite-temperature string method has been applied recently to model the complex *α*-helix to *β*-sheet transition in a *β*-hairpin mini protein in implicit solvent [629]. Transition pathways constructed by string methods have been reported in [630–634]. To fully appreciate the scope of the string method proposed in [343], we additionally note here its application to model in detail the transition of the converter of myosin VI between the PPS and R conformations by computing the associated MFEP for the R ↔ PPS isomerization, the free-energy profile along the transition pathway, and estimating the interconversion rate [635].

String methods make use of the approximation that, with high probability, the flux associated with transition paths is concentrated inside one or a few thin (reaction) tubes. This may not be a reasonable assumption, particularly for complex systems. The WEM is combined with a string method in [636] to address this issue. Another method, proposed in [637] and tested in [638,639], combines a string method with swarms of trajectories [637].

Another drawback of string methods is their computational cost due to the multiple gradient calculations performed on images located far away from the transition state. Many methods are proposed to reduce this computational burden. We note here the growing and the freezing string methods [640–645]. The growing string method attempts to reduce the number of calculations in the iterative steps of string methods. Essentially, two string segments are grown independently from the start and goal structures until they join each other. The freezing string method additionally reduces costs related to the parameterization in string methods. The images are optimized in a direction perpendicular to the progress coordinate with a few conjugate gradient steps and are then frozen in place, effectively constructing an approximate Hessian. Work in [646] demonstrates that this approximation performs as well as growing string methods that use the exact Hessian. As evidenced by the rich number works cited, work on methods for computing transition paths, rates, and transition states is very active.

### Evolutionary Algorithms

An important group of methods to address optimization-related problems in macromolecular modeling consists of evolutionary algorithms (EAs). EAs approach stochastic optimization under the umbrella of evolutionary computation, where the main idea is for computation to mimic the process of evolution and natural selection to find local optima of a complex objective/fitness function. The realization that the potential energy landscape of a macromolecule can be non-linear and multimodal, and that many structure-centric macromolecular modeling problems can be cast as optimization problems makes EAs highly appealing for macromolecular modeling.

Though EAs are highly customizable algorithms, they all follow a simple template. A population of samples of a configuration space (generally referred to as individuals) is evolved over a number of generations. An initialization mechanism specifies the initial population, which can consist of random samples or include configurations known to be local optima (for instance, experimentally-available structures may play this role). The population evolves either over a fixed, user-defined number of generations or until a different termination criterion is reached. In each generation, individuals with high fitness are repeatedly selected and varied upon. The selection mechanism specifies which individuals to select as parents for reproduction. The improvement mechanism consists of reproductive or variation operators, which can be asexual, introducing a mutation on a parent, or sexual, combining the material of two parents at one or more crossover points to generate offspring. A survival mechanism determines which individuals survive to the next generation. In non-overlapping or generational survival mechanisms, the offspring replace the parents. In overlapping ones, a subset of individuals from the combined parent and offspring pool are selected for survival onto the next generation. A comprehensive review of EAs can be found in [647].

EAs are very rich algorithmic frameworks, as different design decisions in the initialization, variation, selection, and survival mechanisms can lead to very different behaviors. The decision on how to represent individuals is key both to the effectiveness and ease with which variation operators can be designed to produce good-quality individuals. EAs that employ crossover in addition to the asexual (mutation) operator are referred to as genetic algorithms (GAs). EAs that additionally incorporate a meme, which is a local improvement operator to improve an offspring and effectively map it to a nearby optimum, are referred to as hybrid or memetic EAs (MAs). The employment of multiple, independent objective functions as opposed to a single fitness function results in multi-objective EAs (MO-EAs). Specific variants that build over GA are respectively referred to as MGAs and MO-GAs.

One of the first EAs for macromolecular structure modeling was a GA, proposed in [648] for the de novo protein structure prediction problem. Work in [648] also demonstrated that EAs are better able to escape local minima of a protein energy function than MC [648]. This result is not surprising, considering that the algorithm able to compute Lennard-Jones optima of atomic clusters in [649] was in fact an EA. Referred to as Basin Hopping, the algorithm was a 1+1 MA, which refers to an MA that has only one parent and one offspring. In a 1+1 MA, the population evolving over generations has size 1, and the offspring competes with the parent. We recall that MA refers to an EA where the offspring is subjected to a local improvement operator (energetic minimization). In Basin Hopping, the offspring replaces the parent with a probability resembling the Metropolis criterion. An MC search can also be viewed as an EA, specifically, a 1+1 EA, and all MC-based methods can be conceptualized as EAs employing highly specific insight about the optimization problem at hand.

Given the early work in [648], EAs have a long history in de novo protein structure prediction. Customized EAs for this problem contain many evolutionary strategies and meta-heuristics, including the employment of a hall of fame to preserve “good” individuals (decoys), tabu search to improve the performance of a meme, co-evolving memes, niching, crowding, twin removal for population diversification, structuring of the solution space to facilitate distributed implementations capable of exploiting parallel computing architectures, and more. The main focus of algorithmic research on EAs is what mechanisms avoid premature convergence and allow finding the global optimum in overly rugged fitness landscapes. This is of particular interest on applications of EAs for different structure-centric problems in macromolecular modeling [650]. A comprehensive review of EAs for de novo protein structure prediction can be found in [651].

Though they have a long history in de novo structure prediction, EAs are not considered among the top performers in this problem for proteins no longer than 200 amino acids. On long protein chains, where off-lattice models result in impractical computational demands, on-lattice EAs are by now the only viable algorithms [652,653]. However, on shorter chains, where off-lattice models can be afforded, the injection of specialized operators (moves), such as molecular fragment replacement, and sophisticated hybrid potential energy functions have allowed rather simple MC-based algorithms to outperform non-customized EAs. Of note here are the Rosetta and Quark methods that often dominate the leader board in the CASP competition [118–120].

Even though EAs have yet to become state of the art in the de novo structure prediction setting, much progress has been made in recent years [390,391,654]. Recently, EAs have incorporated state-of-the-art, off-lattice representations and energy functions to become competitive with MC-based methods such as Rosetta [390,391]. The additional recasting of the structure prediction problem as a multi-objective optimization one has resulted in higher exploration capability and conformation quality over single-objective optimization approaches such as Rosetta [392,655]. EAs are also employed to address protein folding [656].

While there is still much work to be done to demonstrate EAs as the state-of-the-art approaches for de novo structure prediction, there are three domains in macromolecular structure modeling where EAs are by now the best performers: protein-ligand binding, multimeric protein-protein docking, and cryo-EM reconstruction;

In protein-ligand binding, some of the top algorithms are EAs. For instance, Autodock now employs a Lamarckian GA, which has been demonstrated to result in better-quality receptor-ligand bound configurations over the MC-SA algorithm employed in earlier releases [180]. In particular, work in [180] demonstrates that both the Lamarckian GA and a traditional GA can handle ligands of more degrees of freedom than MC-SA, and that the Lamarckian GA outperforms the traditional GA. The latter is due to the fact that in a Lamarckian GA, contrary to the Darwinian model of evolution, where only genetic traits are inheritable, an offspring is replaced with the result of the local improvement operator to which it is subjected. This results in essentially introducing phenotypic traits in the genotypic pool (improvements are passed onto the next generation), per Jean Baptiste Lamarck’s now discredited claim that phenotypic characteristics acquired during an individual’s lifetime can be become inheritable traits; (epigenetics is bringing more credibility, however, to Lamarck’s claims). It is worth pointing out that many MAs (for instance, even Basin Hopping) are Lamarckian EAs. MAs that are not Lamarckian choose not to replace the offspring with the result of the local improvement operator to which it is subjected but use the improved fitness in the survival mechanism; this is known as the Baldwin effect [657].

A domain where EAs are showing promise is in structure prediction for asymmetric, heteromeric assemblies. Currently, the only algorithm that has been shown capable of producing native asymmetric structures of heteromeric assemblies in the absence of wet-laboratory data is Multi-LZerD [292]. Multi-LZerD is a GA that represents multimeric conformations through spanning trees. The nodes in the tree represent the units, and the edges encode the presence of a direct interaction. As presented, Multi-LZerD proceeds over 3,000 generations. While promising, the algorithm incurs a high computational cost to be practical in its current form for multimeric assemblies of more than 6 units.

Another domain where EAs are shown to be highly successful is the simultaneous registration problem in cryo-EM microscopy reconstruction. One issue with cryo-EM is that low-resolution maps are often obtained for large asymmetric and/or dynamic macromolecular assemblies. In such cases, an important problem is how to simultaneously fit known structures of the units in the given map. A GA with specialized variation operators and tabu search has been proposed in [658] to successfully address this problem. This GA has also been used in later work in [659] to trace *α* helices in low- to mid-resolution cryo-EM maps.

While most of the work on EAs in the evolutionary computation community is driven by algorithmic design and analysis of the exploration capability rather than data quality, key ideas and strategies on evolutionary search are proving powerful in enhancing exploration capability in macromolecular structure modeling problems. For instance, several algorithmic decisions on how to select which parents for reproduction, generate offspring, and setup the competition for survival are key for balancing the breadth (exploration) and depth (exploitation) issue in exploration [647]. Lately, interesting ideas from multi-objective optimization are being incorporated in EAs for conformation sampling in de novo protein structure prediction. Namely, instead of pursuing the global minimum of an aggregate energy score, EA-based methods are proposed to obtain conformations that optimize specific sub-groupings of interatomic interactions [392]. EA-based methods are also showing promise in mapping energy landscapes of proteins with large conformational changes [324,660]. Due to the ongoing work in the evolutionary computation community on powerful and effective algorithmic strategies for obtaining solutions of complex objective functions and the realization of outstanding sampling bottlenecks in de novo structure prediction [661], adoption of EAs holds great promise for macromolecular structure modeling.

### Robotics-Inspired Methods

Since simulation of dynamics is the limiting factor in dynamics-based methods, efficiency concerns can be addressed by foregoing or at least delaying dynamics until credible conformational paths have been obtained. A different class of methods focuses not on producing transition trajectories but rather computing a sequence of conformations (a conformational path) with a credible energy profile. The working assumption is that, once obtained, credible conformational paths can then be locally deformed with techniques that consider dynamics to obtain actual transition trajectories. Such methods adapt sampling-based algorithms developed to address the robot motion-planning problem and are thus known as robotics-inspired methods.

The objective in robot motion planning is to obtain paths that take a robot from a start to a goal configuration. The robot motion planning problem bears mechanistic analogies to the problem of computing conformations along a transition trajectory; in both problems the goal is to uncover what of the underlying conformation or configuration space is employed in motions of a mechanical or biological system from a start to a goal conformation or configuration. Analogies between molecular bonds and robot links and atoms and robot joints are made to perform fast molecular kinematics.

Robotics-inspired methods are tree-based or roadmap-based [662]. Tree-based methods grow a tree in conformation space from a given, start to a given, goal conformation representing the structures bridged by the sought transition. The growth of the tree is biased so the goal conformation can be reached in reasonable computational time. As a result, tree-based methods are efficient but limited in their sampling. They are known as single-query methods, as they can only answer one start-to-goal query at a time; that is, only one path of consecutive conformations that connect the start to the goal can be extracted from the tree. Running them multiple times to sample an ensemble of conformational paths for the same query results in an ensemble with high inter-path correlations due to the biasing of the conformation tree. Roadmap-based methods adapt the Probabilistic Road Map (PRM) framework [663]. These methods support multiple queries. Rather than grow a tree in conformation space, these methods detach the sampling of conformations from the structure that encodes neighborhood relationships among conformations in the conformation space. Typically, a sampling stage first provides a discrete representation of the conformation space of interest, and then a roadmap building stage embeds sampled conformations in a graph/roadmap by connecting each one to its nearest neighbors.

Roadmap-based methods bring their own unique set of challenges. Randomly-sampled conformations have very low probability of being in the region of interest for the transition. In particular, for long chains with many degrees of freedom (hundreds of backbone angles in small-to-medium protein chains), a protein conformation sampled at random is very unlikely to be physically realistic. Biased sampling techniques can be used to remedy this issue [664,665], but it is hard to know which ones will focus sampling to regions of interest for the transition. In addition, both roadmap- and tree-based methods rely on local planners or local deformation techniques to connect two neighboring conformations. It is hard to find reasonable local planners for protein conformations. A linear interpolation is often carried over the employed parameters, typically backbone angles, but this can produce unrealistic conformations, and a lot of time can be spent energetically refining these conformations. Recent work is considering complex local planners that are not based on interpolation but are instead re-formulations of the motion computation problem. Recent work in [666] introduces a prioritized path sampling scheme to address the computational demands of complex local planners in roadmap-based methods for protein motion computation.

Roadmap-based methods have been employed to model unfolding of small proteins [665,667]. Tree-based methods have been employed to model conformational changes and flexibility, predict the native structure, and compute conformational paths connecting given structural states [351,352,387,668–670]. In particular, the T-RRT method described in [351] and the PDST method described in [352] have focused on the problem of computing conformational paths connecting two given structures. While T-RRT has been shown to connect known low-energy states of the dialanine peptide (two amino acids long) [351], the PDST method has been shown to produce credible information on the order of conformational changes connecting stable states of large proteins (200–500 amino acids long) [352]. Both methods control the dimensionality of the conformation space by either focusing on systems with few amino acids [351] or by employing coarse-grained representations to reduce the number of modeled parameters in large proteins [352]. The tree-based method in [353] employs the fragment replacement technique to reduce the dimensionality of the conformation space and sample conformational paths connecting two given structural states of proteins ranging from from a few dozen to a few hundred amino acids. At each iteration, a conformation in the tree is selected for expansion. The expansion employs molecular fragment replacement and the Metropolis criterion to bias the tree towards low-energy conformations over time. The selection penalizes the tree from growing towards regions of the conformation space that have been oversampled, thus resulting in enhanced sampling of the conformation space.

## Conclusions

This review has highlighted the breadth and depth of research in macromolecular modeling and simulation. A plethora of computational methods have been developed to study a wide spectrum of molecular events. QM methods are used to study molecular electronic structures and obtain detailed and accurate electronic structure calculations. Work in [671] employs such calculations to correlate quantum descriptors and the biological activity of 13 quinoxaline drug compounds and then suggest effective compounds against drug-resistant Mycobacterium tuberculosis. Recent efforts in quantum chemistry are devoted to circumventing computational bottlenecks of large-scale electronic structure calculations and extending applicability to molecular systems composed of hundreds of atoms [672]. At present, QM methods have too high a computational cost to be a competitive alternative to MD or MC methods and their variants. For this reason, the focus of this review has been on MM methods, such as MD and variations, which are the methods of choice to study macromolecular structure and dynamics. It should be noted that hybrid, QM/MM methods exist and are the methods of choice for modeling reactions in biomolecular systems [673].

One of the major themes in MM-based macromolecular modeling is the choice of resolution or detail. As this review has summarized, atomistic, explicit solvent MD simulations are becoming more affordable, both due to improvements in hardware and techniques that allow aggressive parallelization. Despite the challenges posed by the disparate spatial and time scales employed by macromolecules flexing their structures and interacting with their environment, significant algorithmic and hardware advances have allowed breaking the millisecond barrier [147]. Dynamical processes that involve millions of atoms can now be characterized. For example, work in [674] tracks via MD simulations the microsecond-long atomic motions of 1.2 million particles to study the dissolution of the capsid of the satellite tobacco necrosis virus.

MD and non-MD methods that employ reduced, coarse-grained macromolecular models are often regarded as “cheaper” albeit less accurate alternatives to atomistic MD methods. Such cheaper methods currently complement or facilitate atomistic MD-based studies. For example, protein docking methods are routinely employed to assist cryo-EM in resolving structures of molecular assemblies. Once such methods narrow down the possible conformation space, subsequent atomistic MD simulations are employed to make final predictions by examining stability and dynamics [111].

In some settings, these cheaper methods provide the only practical approach. Even with various accelerated MD simulations, mapping of protein energy landscapes remains challenging. For example, work in [10] shows that the sampling capability of accelerated MD greatly depends on the structure used to initiate a trajectory [10]. In our own laboratories, we have been able to compare the cheaper methods to published atomistic MD simulations of H-Ras [660]. In particular, on H-Ras, the evolutionary algorithm in [660] is able to map the energy landscape of H-Ras wildtype and selected variants in atomistic detail better than what can currently be achieved via known MD methods.

In MD-based research, two different directions seem to be pursued by researchers at the moment. The first involves the employment of very long MD simulations, made possible by complex MD-customized architectures, like Anton. Thermodynamic and kinetic quantities can be readily extracted from such simulations. The second involves the employment of several short, off-equilibrium MD simulations, which allows the employment of parallel architectures but necessitates the employment of statistical models, such as Markov state models, to collect and organize the simulations to describe the long-time behavior of a system. Both directions are exciting and complementary. In particular, the second direction is leading to advances in the combination of continuous and discrete models for expediting modeling of long-time scale phenomena and is likely to lead to further algorithmic advancements. Within each of these directions, several open questions remain for researchers to pursue. A combination of both directions, dedicated architectures and continuous and discrete models promises to push the spatial and time scales that can be observed in silico even further.

As summarized in this review, many non-MD algorithmic frameworks are being pursued to model different aspects of macromolecular structure and dynamics. Often, these frameworks are inspired or initiated from diverse communities of researchers. Of note here are evolutionary algorithms and robotics-inspired algorithms. While components of these algorithms are often investigated in detail in each of the corresponding communities, the focus in these communities has traditionally been on often on computational performance rather than quality of findings. Broad employment of these algorithms as tools complementary to MD is currently challenged by an inability to demonstrate utility on a broad class of macromolecular systems and validate findings with existing wet-laboratory or MD-based studies. Nonetheless, a growing body of researchers within each of these communities is introducing treatments focused on both computational performance and data quality.

This review has summarized the current state of the art in diverse application areas. An emerging theme is the need to characterize in detail the structural flexibility of a macromolecular system under specific conditions. While great progress is being made, computing a conformation ensemble consistent with explicit or implicit constraints is likely to motivate the development of novel algorithms for years to come.

Many other directions of research in macromolecular modeling and simulation could not be described in detail here. These include the development of accurate and sensitive molecular force fields [140,141] for macromolecular simulation, the development of increasingly accurate coarse-grained representations of macromolecules, solvent models, and multiscaling techniques [76,142–144], decoy/model selection algorithms [675] in de novo structure prediction, as well as the development of algorithmic tools to assist structure resolution in the wet laboratory [676,677]. Additionally, while this review highlights some of the unique challenges posed by intrinsically disordered proteins and regions, it does not provide an overview of similar challenges posed by membrane proteins. The reader is referred to work in [678] for a review of such challenges and algorithmic advancements.

Expected advances in each of the reviewed application areas promise to provide us with a more comprehensive and detailed understanding of our biology. In particular, unraveling the behavior of macromolecules in isolation and assembly will help us understand the molecular basis of mechanisms in the healthy and diseased cell. A truly synergistic employment of in-silico and wet-lab research to unravel molecular mechanisms also promises to lead to better therapeutics for combating cancer, neurodegenerative disorders, infections, and other important human disorders of our time. The journey into the future of computational structural biology promises to be exciting, and we hope that this review has inspired a few more researchers to join us on this journey.

## Supporting Information

S1 Text Abbreviations in alphabetical order. Abbreviations are provided for names of methods and proteins.
(PDF)

## References

[1] Soto C. Protein misfolding and neurodegeneration. JAMA Neurology. 2008;65(2):184–189. 10.1001/archneurol.2007.56 18268186

[2] Uversky VN. Intrinsic disorder in proteins associated with neurodegenerative diseases. Front Biosci. 2009;14:5188–5238. 10.2741/3594 19482612

[3] Fernández-Medarde A, Santos E. Ras in cancer and developmental diseases. Genes Cancer. 2011;2(3):344–358. 10.1177/1947601911411084 21779504 PMC3128640

[4] Neudecker P, Robustelli P, Cavalli A, Walsh P, Lundstr om P, Zarrine-Afsar A, et al Structure of an intermediate state in protein folding and aggregation. Science. 2012;336(6079):362–366. 10.1126/science.1214203 22517863

[5] Onuchic JN, Luthey-Schulten Z, Wolynes PG. Theory of protein folding: the energy landscape perspective. Annu Rev Phys Chem. 1997;48:545–600. 9348663 10.1146/annurev.physchem.48.1.545

[6] Ozenne V, Schneider R, Yao M, Huang JR, Salmon L, Zweckstetter M, et al Mapping the potential energy landscape of intrinsically disordered proteins at amino acid resolution. J Am Chem Soc. 2012;134(36):15138–15148. 10.1021/ja306905s 22901047

[7] Levy Y, Jortner J, Becker OM. Solvent effects on the energy landscapes and folding kinetics of polyalanine. Proc Natl Acad Sci USA. 2001;98(5):2188–2193. 11226214 10.1073/pnas.041611998 PMC30113

[8] Miao Y, Nichols SE, McCammon JA. Free energy landscapes of G-protein-coupled receptors, explored by accelerated molecular dynamics. Phys Chem Chem Phys. 2014;16(14):6398–6406. 10.1039/c3cp53962h 24445284 PMC3960983

[9] Gorfe AA, Grant BJ, McCammon JA. Mapping the nucleotide and isoform-dependent structural and dynamical features of Ras proteins. Structure. 2008;16(6):885–896. 10.1016/j.str.2008.03.009 18547521 PMC2519881

[10] Grant BJ, Gorfe AA, McCammon JA. Ras Conformational Switching: Simulating Nucleotide-Dependent Conformational Transitions with Accelerated Molecular Dynamics. PLoS Comput Biol. 2009;5(3):e1000325 10.1371/journal.pcbi.1000325 19300489 PMC2651530

[11] Anfinsen CB. Principles that govern the folding of protein chains. Science. 1973;181(4096):223–230. 4124164 10.1126/science.181.4096.223

[12] Fersht AR. Structure and Mechanism in Protein Science A Guide to Enzyme Catalysis and Protein Folding. 3rd ed. New York, NY: W. H. Freeman and Co.; 1999.

[13] Frauenfelder H, Sligar SG, Wolynes PG. The energy landscapes and motion on proteins. Science. 1991;254(5038):1598–1603. 1749933 10.1126/science.1749933

[14] Sawaya MR, Kraut J. Loop and Domain Movements in the Mechanism of E. Coli Dihydrofolate Reductase: Crystallographic Evidence. Biochemistry. 1997;36(3):586–603. 9012674 10.1021/bi962337c

[15] Radkiewicz JL, Brooks CL. Protein dynamics in enzymatic catalysis: Exploration of dihydrofolate reductase. J Am Chem Soc. 2000;122(2):225–231.

[16] Vendruscolo M, Dobson CM. Dynamic visions of enzymatic reactions. Science. 2006;313(5793):1586–1587. 16973868 10.1126/science.1132851

[17] Clore GM, Schwieters CD. How much backbone motion in ubiquitin is required to account for dipolar coupling data measured in multiple alignment media as assessed by independent cross-validation? J Am Chem Soc. 2004;126(9):2923–2938. 14995210 10.1021/ja0386804

[18] Henzler-Wildman K, Kern D. Dynamic personalities of proteins. Nature. 2007;450:964–972. 18075575 10.1038/nature06522

[19] Okazaki K, Koga N, Takada S, Onuchic JN, Wolynes PG. Multiple-basin energy landscapes for large-amplitude conformational motions of proteins: Structure-based molecular dynamics simulations. Proc Natl Acad Sci USA. 2006;103(32):11844–11849. 16877541 10.1073/pnas.0604375103 PMC1567665

[20] Hub JS, de Groot BL. Detection of Functional Modes in Protein Dynamics. PLoS Comput Biol. 2009;5(8):e1000480 10.1371/journal.pcbi.1000480 19714202 PMC2721685

[21] Bahar I, Lezon TR, Yang LW, Eyal E. Global dynamics of proteins: bridging between structure and function. Annu Rev Biophys. 2010;39:23–42. 10.1146/annurev.biophys.093008.131258 20192781 PMC2938190

[22] Boehr DD, Wright PE. How do proteins interact? Science. 2008;320(5882):1429–1430. 10.1126/science.1158818 18556537

[23] Boehr DD, Nussinov R, Wright PE. The role of dynamic conformational ensembles in biomolecular recognition. Nature Chem Biol. 2009;5(11):789–96. 19841628 10.1038/nchembio.232 PMC2916928

[24] Feynman RP, Leighton RB, Sands M. The Feynman Lectures on Physics. Reading, MA: Addison-Wesley; 1963.

[25] McCammon JA, Gelin BR, Karplus M. Dynamics of folded proteins. Nature. 1977;267:585–590. 301613 10.1038/267585a0

[26] Cooper A. Protein fluctuations and the thermodynamic uncertainty principle. Prog Biophys Mol Biol. 1984;44(3):181–214. 6390520 10.1016/0079-6107(84)90008-7

[27] Frauenfelder H, Wolynes PG. Biomolecules: Where the Physics of Complexity and Simplicity Meet. Physics Today. 1994;47(2):58–64.

[28] Dill KA, Chan HS. From Levinthal to pathways to funnels. Nat Struct Biol. 1997;4(1):10–19. 8989315 10.1038/nsb0197-10

[29] Heymann JB, Conway JF, Steven AC. Molecular dynamics of protein complexes from four-dimensional cryo-electron microscopy. J Struct Biol. 2004;147(3):291–301. 15450298 10.1016/j.jsb.2004.02.006

[30] Kleckner IR, Foster MP. An introduction to NMR-based approaches for measuring protein dynamics. Biochim Biophys Acta. 2011;14(8):942–968. 10.1016/j.bbapap.2010.10.012 PMC3061256 21059410

[31] Fenwick RB, van den Bedem H, Fraser JS, Wright PE. Integrated description of protein dynamics from room-temperature X-ray crystallography and NMR. Proc Natl Acad Sci USA. 2014;111(4):E445–E454. 10.1073/pnas.1323440111 24474795 PMC3910589

[32] Best RB, Vendruscolo M. Determination of ensembles of structures consistent with NMR order parameters. J Am Chem Soc. 2004;126(26):8090–8091. 15225030 10.1021/ja0396955

[33] Berlin K, Castañeda CA, Schneidman-Duhovny D, Sali A, Nava-Tudela A, Fushman D. Recovering a representative conformational ensemble from underdetermined macromolecular structural data. J Am Chem Soc. 2013;135(44):16595–16609. 24093873 10.1021/ja4083717 PMC3902174

[34] De Simone A, Montalvao RW, Dobson CM, Vendruscolo M. Characterization of the Interdomain Motions in Hen Lysozyme Using Residual Dipolar Couplings as Replica-Averaged Structural Restraints in Molecular Dynamics Simulations. Biochemistry. 2013;52(37):6480–6486. 10.1021/bi4007513 23941501

[35] Lindorff-Larsen K, Best RB, DePristo MA, Dobson CM, Vendruscolo M. Simultaneous determination of protein structure and dynamics. Nature. 2005;433(7022):128–132. 15650731 10.1038/nature03199

[36] Vendruscolo M, Pacci E, Dobson C, Karplus M. Rare Fluctuations of Native Proteins Sampled by Equilibrium Hydrogen Exchange. J Am Chem Soc. 2003;125(51):15686–15687. 14677926 10.1021/ja036523z

[37] Kay LE. Protein Dynamics from NMR. Nat Struct Biol. 1998;5(2–3):513–517. 9665181 10.1038/755

[38] Kay LE. NMR studies of protein structure and dynamics. J Magn Reson. 2005;173(2):193–207. 15780912 10.1016/j.jmr.2004.11.021

[39] Torella JP, Holden SJ, Santoso Y, Hohlbein J, Kapanidis AN. Identifying Molecular Dynamics in Single-Molecule FRET Experiments with Burst Variance Analysis. Biophys J. 2011;100(6):1568–1577. 10.1016/j.bpj.2011.01.066 21402040 PMC3059737

[40] Zhu G, editor. NMR of proteins and small biomolecules. vol. 326 of Topics in Current Chemistry Springer-Verlag; 2012.

[41] Karam P, Powdrill MH, Liu HW, Vasquez C, Mah W, Bernatchez J, et al Dynamics of hepatitis C Virus (HCV) RNA-dependent RNA Polymerase NS5B in Complex with RNA. J Biol Chem. 2014;289(20):14399–14411. 10.1074/jbc.M113.529743 24692556 PMC4022906

[42] Moerner WE, Fromm DP. Methods of single-molecule fluorescence spectroscopy. Rev Scientific Instruments. 2003;74(8):3597–3619.

[43] Greenleaf WJ, Woodside MT, Block SM. High-Resolution, Single-Molecule Measurements of Biomolecular Motion. Annu Rev Biophys Biomol Struct. 2007;36:171–190. 17328679 10.1146/annurev.biophys.36.101106.101451 PMC1945240

[44] Michalet X, Weiss S, Jäger M. Single-Molecule Fluorescence Studies of Protein Folding and Conformational Dynamics. Chem Rev. 2006;106(5):1785–1813. 16683755 10.1021/cr0404343 PMC2569857

[45] Diekmann S, Hoischen C. Biomolecular dynamics and binding studies in the living cell. Physics of Life Reviews. 2014;11(1):1–30. 10.1016/j.plrev.2013.11.011 24486003

[46] Hohlbein J, Craggs TD, Cordes T. Alternating-laser excitation: single-molecule FRET and beyond. Chem Soc Rev. 2014;43:1156–1171. 10.1039/c3cs60233h 24037326

[47] Schlau-Cohen GS, Wang Q, Southall J, Cogdell RJ, Moerner WE. Single-molecule spectroscopy reveals photosynthetic LH2 complexes switch between emissive states. Proc Natl Acad Sci USA. 2013;110(27):10899–10903. 10.1073/pnas.1310222110 23776245 PMC3704035

[48] Moffat K. The frontiers of time-resolved macromolecular crystallography: movies and chirped X-ray pulses. Faraday Discuss. 2003;122(79–88):65–77. 12555850 10.1039/b201620f

[49] Schotte F, Lim M, Jackson TA, Smirnov AV, Soman J, Olson JS, et al Watching a protein as it functions with 150-ps time-resolved X-ray crystallography. Science. 2003;300(5627):1944–1947. 12817148 10.1126/science.1078797

[50] Roy R, Hohng S, Ha T. A practical guide to single-molecule FRET. Nature Methods. 2008;5(6):507–516. 10.1038/nmeth.1208 18511918 PMC3769523

[51] Lee HM, M KS, Kim HM, Suh YD. Single-molecule surface-enhanced Raman spectroscopy: a perspective on the current status. Phys Chem Chem Phys. 2013;15:5276–5287. 10.1039/c3cp44463e 23525118

[52] Socher E, Imperiali B. FRET-CAPTURE: A sensitive method for the detection of dynamic protein interactions. Chem Biochem. 2013;14(1):53–57. 10.1002/cbic.201200700 PMC3776414 23239458

[53] Gall A, Ilioaia C, Krüger TP, Novoderezhkin VI, Robert B, van Grondelle R. Conformational Switching in a Light-Harvesting Protein as Followed by Single-Molecule Spectroscopy. Biophys J. 2015;108(11):2713–2720. 10.1016/j.bpj.2015.04.017 26039172 PMC4457476

[54] Ådén J, Wolf-Watz M. NMR Identification of Transient Complexes Critical to Adenylate Kinase Catalysis. J Am Chem Soc. 2007;129(45):14003–14012. 17935333 10.1021/ja075055g

[55] Russel D, Lasker K, Phillips J, Schneidman-Duhovny D, Veláquez-Muriel JA, Sali A. The structural dynamics of macromolecular processes. Curr Opin Cell Biol. 2009;21(1):97–108. 10.1016/j.ceb.2009.01.022 19223165 PMC2774249

[56] Taketomi H, Ueda Y, Go N. Studies on protein folding, unfolding and fluctuations by computer simulation: The effect of specific amino acid sequence represented by specific inter-unit interactions. Int J Peptide Prot Res. 1975;7(6):445–459. 1201909

[57] Bashford D, Karplus M. pKa’s of ionizable groups in proteins: atomic detail from a continuum electrostatic model. Biochemistry. 1990;29(44):10219–10225. 2271649 10.1021/bi00496a010

[58] Lau KF, Dill AK. A lattice statistical mechanics model of the conformational and sequence spaces of of proteins. Macromolecules. 1989;22(10):3986–3997.

[59] Unger R, Moult J. Finding lowest free energy conformation of a protein is an NP-hard problem: Proof and implications. Bull Math Biol. 1993;55(6):1183–1198. 8281131 10.1007/BF02460703

[60] Hart WE, Istrail S. Robust Proofs of NP-Hardness for Protein Folding: General Lattices and Energy Potentials. J Comp Biol. 1997;4(1):1–22. 10.1089/cmb.1997.4.1 9109034

[61] Kendrew JC, Bodo G, Dintzis HM, Parrish RG, Wyckoff H, Phillips DC. A three-dimensional model of the myoglobin molecule obtained by X-ray analysis. Nature. 1958;181(4610):662–666. 13517261 10.1038/181662a0

[62] Kendrew JC, Dickerson RE, Strandberg BE, Hart RG, Davies DR, Phillips DC, et al Structure of myoglobin: A three-dimensional fourier synthesis at 2 Å resolution. Nature. 1960;185(4711):422–427. 18990802 10.1038/185422a0

[63] Phillips DC. The Hen Egg-White Lysozyme Molecule. Proc Natl Acad Sci USA. 1967;57(3):483–495. 10.1098/rspb.1967.0034 4382800

[64] Berman HM, Henrick K, Nakamura H. Announcing the worldwide Protein Data Bank. Nat Struct Biol. 2003;10(12):980–980. 14634627 10.1038/nsb1203-980

[65] Verlet L. Computer "experiments" on Classical Fluids. I. Thermodynamical Properties of Lennard-Jones Molecules. Phys Rev Lett. 1967;159:98–103.

[66] Brooks BR, Bruccoleri RE, Olafson BD, States DJ, Swaminathan S, Karplus M. CHARMM: a program for macromolecular energy, minimization, and dynamics calculations. J Comput Chem. 1983;4(2):187–217.

[67] Karplus M, McCammon JA. Protein structural fluctuations during a period of 100 ps. Nature. 1979;277(5697):578 763343 10.1038/277578a0

[68] Levitt M, Warshel A. Computer simulation of protein folding. Nature. 1975;253(5494):94–96. 10.1038/253694a0 1167625

[69] Lifson S, Warshel A. A Consistent Force Field for Calculation on Conformations, Vibrational Spectra and Enthalpies of Cycloalkanes and n-Alkane Moleculesâ. J Phys Chem. 1968;49:5116–5129.

[70] Levitt M, Lifson S. Refinement of Protein Conformations Using a Macromolecular Energy Minimization Procedure. J Mol Biol. 1969;46:269–279. 5360040 10.1016/0022-2836(69)90421-5

[71] Gibson KD, Scheraga A. Minimization of Polypeptide Energy. I. Preliminary Structures of Bovine Pancreatic Ribonuclease S-peptide. Proc Natl Acad Sci USA. 1967;58:420–427. 5233450 10.1073/pnas.58.2.420 PMC335651

[72] Levitt M. A Simplified Representation of Protein Conformations for Rapid Simulation of Protein Folding. J Mol Biol. 1976;104:59–107. 957439 10.1016/0022-2836(76)90004-8

[73] A W, Levitt M. Theoretical Studies of Enzymatic Reactions: Dielectric, Electrostatic and Steric Stabilization of the Carbonium Ion in the Reaction of Lysozyme. J Mol Biol. 1976;103:227–249. 985660 10.1016/0022-2836(76)90311-9

[74] Warshel A. Computer simulations of enzyme catalysis: Methods, progress, and insights. Annu Rev Biophys Biomol Struct. 2003;32:425–443. 12574064 10.1146/annurev.biophys.32.110601.141807

[75] Donchev AG, Ozrin VD, Subbotin MV, Tarasov OV, Tarasov VI. A Quantum Mechanical Polarizable Force Field for Biomolecular Interactions. Proc Natl Acad Sci USA. 2005;102(22):7829–7834. 15911753 10.1073/pnas.0502962102 PMC1142387

[76] Zhou H. Theoretical frameworks for multiscale modeling and simulation. Curr Opinion Struct Biol. 2014;25:67–76. 10.1016/j.sbi.2014.01.004 PMC4040314 24492203

[77] Kamerlin SC, Haranczyk M, Warshel A. Progresses in Ab Initio QM/MM Free Energy Simulations of Electrostatic Energies in Proteins: Accelerated QM/MM Studies of pKa, Redox Reactions and Solvation Free Energies. J Phys Chem B. 2009;113(5):1253–1272. 10.1021/jp8071712 19055405 PMC2679392

[78] Kamerlin SCL, Vicatos S, Dryga A, Warshel A. Coarse-Grained (Multiscale) Simulations in Studies of Biophysical and Chemical Systems. Ann Rev Phys Chem. 2011;62(1):41–64. 21034218 10.1146/annurev-physchem-032210-103335

[79] Plotnikov NV, Warshel A. Exploring, Refining, and Validating the Paradynamics QM/MM Sampling. J Phys Chem B. 2012;116(34):10342–10356. 10.1021/jp304678d 22853800 PMC12401620

[80] Vicatos S, Rychkova A, Mukherjee S, Warshel A. An effective Coarse-grained model for biological simulations: Recent refinements and validations. Proteins: Structure, Function, and Bioinformatics. 2014;82(7):1168–1185. 10.1002/prot.24482 PMC4109661 25050439

[81] Warshel A. Energetics of enzyme catalysis. Proc Natl Acad Sci USA. 1978;75(11):5250–5254. 281676 10.1073/pnas.75.11.5250 PMC392938

[82] Mukherjee S, Warshel A. Electrostatic origin of the mechanochemical rotary mechanism and the catalytic dwell of F1-ATPase. Proc Natl Acad Sci USA. 2011;108(51):20550–20555. 10.1073/pnas.1117024108 22143769 PMC3251122

[83] Mukherjee S, Warshel A. Realistic simulations of the coupling between the protomotive force and the mechanical rotation of the F0-ATPase. Proc Natl Acad Sci USA. 2012;109(3):14876–14881. 22927379 10.1073/pnas.1212841109 PMC3443130

[84] Dryga A, Chakrabarty S, Vicatos S, Warshel A. Realistic simulation of the activation of voltage-gated ion channels. Proc Natl Acad Sci USA. 2011;109(9):3335–3340. 10.1073/pnas.1121094109 PMC3295319 22331900

[85] Rychkova A, Mukherjee S, Bora RP, Warshel A. Simulating the pulling of stalled elongated peptide from the ribosome by the translocon. Proc Natl Acad Sci USA. 2013;110(25):10195–10200. 10.1073/pnas.1307869110 23729811 PMC3690858

[86] Mukherjee S, Warshel A. Electrostatic origin of the unidirectionality of walking myosin V motors. Proc Natl Acad Sci USA. 2013;110(43):17326–17331. 10.1073/pnas.1317641110 24106304 PMC3808596

[87] Ma J, Sigler PB, Xu Z, Karplus M. A Dynamic Model for the Allosteric Mechanism of GroEL. J Mol Biol. 2000;302:303–313. 10970735 10.1006/jmbi.2000.4014

[88] Henzler-Wildman KA, Thai V, Lei M, Ott M, Wolf-Watz M, Fenn T, et al Intrinsic motions along an enzymatic reaction trajectory. Nature. 2007;450(7171):838–844. 18026086 10.1038/nature06410

[89] Gao YQ, Yang W, Karplus M. A structure-based model for the synthesis and hydrolysis of ATP by F1-ATPase. Cell. 2005;123(2):195–205. 16239139 10.1016/j.cell.2005.10.001

[90] Pu J, Karplus M. How subunit coupling produces the γ-subunit rotary motion in F1-ATPase. Proc Natl Acad Sci USA. 2008;105(4):1192–1197. 10.1073/pnas.0708746105 18216260 PMC2234114

[91] Scarabelli G, Grant BJ. Mapping the Structural and Dynamical Features of Kinesin Motor Domains. PLoS Comput Biol. 2013;9(11):e1003329 10.1371/journal.pcbi.1003329 24244137 PMC3820509

[92] Metropolis N, Rosenbluth AW, Rosenbluth MN, Teller AH, Teller E. Equation of state calculations by fast computing machines. J Chem Phys. 1953;21(6):1087–1092.

[93] Torrie GM, Valleau JP. Nonphysical sampling distributions in Monte Carlo free-energy estimation: umbrella sampling. J Comput Phys. 1977;23(2):187–199.

[94] Li Z, Scheraga HA. Monte Carlo-minimization approach to the multiple-minima problem in protein folding. Proc Natl Acad Sci USA. 1987;84(19):6611–6615. 3477791 10.1073/pnas.84.19.6611 PMC299132

[95] Dinner AR, Sali A, Karplus M. The folding mechanism of larger model proteins: role of native structure. Proc Natl Acad Sci USA. 1996;93(16):8356–8361. 8710875 10.1073/pnas.93.16.8356 PMC38675

[96] Lee J, Scheraga HA, Rackovsky S. New optimization method for conformational energy calculations on polypeptides: Conformational space annealing. J Comput Chem. 1997;18(9):1222–1232.

[97] Lee J, Scheraga HA, Rackovsky S. Conformational analysis of the 20-residue membrane-bound portion of melittin by conformational space annealing. Biopolymers. 1998;46(2):103–115. 9664844 10.1002/(SICI)1097-0282(199808)46:2<103::AID-BIP5>3.0.CO;2-Q

[98] Lee J, Scheraga HA. Conformational space annealing by parallel computations: Extensive conformational search of Met-enkephalin and of the 20-residue membrane-bound portion of melittin. Int J Quantum Chem. 1999;75(3):255–265.

[99] Voter AF. Introduction to the Kinetic Monte Carlo Method In: Sickafus KE, Kotomin EA, Uberuaga BP, editors. Radiation Effects in Solids. vol. 235 of NATO Science Series Springer Verlag; 2007 p. 1–23.

[100] Levitt M. The birth of computational structural biology. Nat Struct Biol. 2001;8:392–393. 11323711 10.1038/87545

[101] Karplus M. Development of multiscale models for complex chemical systems from H+H2 to Biomolecules. Nobel Lecture. 2013;p. 1–33. Available from: http://www.nobelprize.org/nobel_prizes/chemistry/laureates/2013/karplus-lecture.pdf. 10.1002/anie.201403924 25066036

[102] Warshel A. Multiscale modeling of biological functions: from enzymes to molecular machines. Nobel Lecture. 2013;p. 1–25. Available from: http://www.nobelprize.org/nobel_prizes/chemistry/laureates/2013/warshel-lecture.pdf. 10.1002/anie.201403689 PMC4948593 25060243

[103] Levitt M. Birth and future of multiscale modeling for macromolecular systems. Nobel Lecture. 2013;p. 1–31. Available from: http://www.nobelprize.org/nobel_prizes/chemistry/laureates/2013/levitt-lecture.pdf. 10.1002/anie.201403691 25100216

[104] Piana S, Lindorff-Larsen K, Shaw DE. Protein folding kinetics and thermodynamics from atomistic simulation. Proc Natl Acad Sci USA. 2012;109(44):17845–17850. 10.1073/pnas.1201811109 22822217 PMC3497772

[105] Lindorff-Larsen K, Piana S, Dror RO, Shaw DE. How fast-folding proteins fold. Science. 2011;334(6055):517–520. 10.1126/science.1208351 22034434

[106] Stone JE, Phillips JC, Freddolino PL, Hardy DJ, Trabuco LG, Schulten K. Accelerating molecular modeling applications with graphics processors. J Comput Chem. 2007;28(16):2618–2640. 17894371 10.1002/jcc.20829

[107] Harvey MJ, Giupponi G, de Fabritiis G. ACEMD: Accelerating Biomolecular Dynamics in the microsecond timescale. J Comput Theor Chem. 2009;5(6):1632–1639. 10.1021/ct9000685 26609855

[108] Tanner DE, Phillips JC, Schulten K. GPU/CPU Algorithm for Generalized Born/Solvent-Accessible Surface Area Implicit Solvent Calculations. J Chem Theory Comput. 2012;8(7):2521–2530. 23049488 10.1021/ct3003089 PMC3464051

[109] G otz AW, Williamson MJ, Xu D, Poole D, Le Grand S, Walker RC. Routine Microsecond Molecular Dynamics Simulations with AMBER on GPUs. 1. Generalized Born. J Chem Theory Comput. 2012;8(5):1542–1555. 22582031 10.1021/ct200909j PMC3348677

[110] Dubrow A. What Got Done in One Year at NSF’s Stampede Supercomputer. Comput Sci Eng. 2015;17(2):83–88.

[111] Zhao G, Perilla JR, Yufenyuy EL, Meng X, Chen B, Ning J, et al Mature HIV-1 capsid structure by cryo-electron microscopy and all-atom molecular dynamics. Nature. 2013;497(7451):643–646. 10.1038/nature12162 23719463 PMC3729984

[112] Perilla JR, Goh BC, Cassidy CK, Liu B, Bernardi RC, Rudack T, et al Molecular dynamics simulations of large macromolecular complexes. Curr Opin Struct Biol. 2015;31:64–74. 10.1016/j.sbi.2015.03.007 25845770 PMC4476923

[113] Fattebert JL, Richards DF, Glosli JN. Dynamic load balancing algorithm for molecular dynamics based on Voronoi cells domain decompositions. Comput Phys Communic. 2012;183(12):2608–2615.

[114] Proctor AJ, Lipscomb TJ, Zou A, Anderson JA, Cho SS. Performance Analyses of a Parallel Verlet Neighbor List Algorithm for GPU-Optimized MD Simulations; 2012.

[115] Batcho P, Case DA, Schlick T. Optimized particle-mesh Ewald/multiple-time step integration for molecular dynamics simulations. J Chem Phys. 2001;115(9):4003–4018.

[116] Phillips JC, Braun R, Wang W, Gumbart J, Tajkhorshid E, Villa E, et al Scalable molecular dynamics with NAMD. J Comput Chem. 2005;26(16):1781–1802. 16222654 10.1002/jcc.20289 PMC2486339

[117] Bradley P, Misura KMS, Baker D. Toward High-Resolution de Novo Structure Prediction for Small Proteins. Science. 2005;309(5742):1868–1871. 16166519 10.1126/science.1113801

[118] Leaver-Fay A, Tyka M, Lewis SM, Lange OF, Thompson J, Jacak R, et al ROSETTA3: an object-oriented software suite for the simulation and design of macromolecules. Methods Enzymol. 2011;487:545–574. 10.1016/B978-0-12-381270-4.00019-6 21187238 PMC4083816

[119] Xu D, Zhang Y. Ab initio protein structure assembly using continuous structure fragments and optimized knowledge-based force field. Proteins: Struct Funct Bioinf. 2012;80(7):1715–1735. 10.1002/prot.24065 PMC3370074 22411565

[120] Zhang Y. Interplay of I-TASSER and QUARK for template-based and ab initio protein structure prediction in CASP10. Proteins. 2014;82(Suppl 2):175–187. 10.1002/prot.24341 23760925 PMC4067246

[121] Grant BJ, Gorfe AA, McCammon JA. Large conformational changes in proteins: signaling and other functions. Curr Opinion Struct Biol. 2010;20(2):142–147. 10.1016/j.sbi.2009.12.004 PMC2866511 20060708

[122] Prakash P, Gorfe AA. Lessons from computer simulations of Ras proteins in solution and in membrane. Biochim Biophys Acta. 2013;1830(11):5211–5218. 10.1016/j.bbagen.2013.07.024 23906604 PMC3825463

[123] Noé F, Schutte C, Vanden-Eijnden E, Reich L, Weikl TR. Constructing the equilibrium ensemble of folding pathways from short off-equilibrium simulations. Proc Natl Acad Sci USA. 2009;106(45):19011–19016. 10.1073/pnas.0905466106 19887634 PMC2772816

[124] Whitford PC, Sanbonmatsu KY, Onuchic JN. Biomolecular dynamics: order-disorder transitions and energy landscapess. Reports on Progress in Physics. 2012;75(7):076601 10.1088/0034-4885/75/7/076601 22790780 PMC3695400

[125] Shehu A, Kavraki LE, Clementi C. Unfolding the Fold of Cyclic Cysteine-rich Peptides. Protein Sci. 2008;17(3):482–493. 10.1110/ps.073142708 18287281 PMC2248317

[126] Shehu A, Kavraki LE, Clementi C. Multiscale Characterization of Protein Conformational Ensembles. Proteins: Struct Funct Bioinf. 2009;76(4):837–851. 10.1002/prot.22390 PMC3164158 19280604

[127] Diaz JF, Wroblowski B, Schlitter J, Engelborghs Y. Calculation of pathways for the conformational transition between the GTP- and GDP-bound states of the Ha-ras-p21 protein: calculations with explicit solvent simulations and comparison with calculations in vacuum. Proteins. 1997;28(3):434–451. 9223188

[128] Malmstrom RD, Lee CT, Van Wart AT, Amaro RE. Application of Molecular-Dynamics Based Markov State Models to Functional Proteins. J Chem Theory Comput. 2014;10(7):2648–2657. 25473382 10.1021/ct5002363 PMC4248791

[129] Maragliano L, Vanden-Eijnden E, Roux B. Free Energy and Kinetics of Conformational Transitions from Voronoi Tessellated Milestoning with Restraining Potentials. J Chem Theory Comput. 2009;5(10):2589–2594. 20354583 10.1021/ct900279z PMC2846710

[130] Franklin J, Koehl P, Doniach S, Delarue M. MinActionPath: maximum likelihood trajectory for large-scale structural transitions in a coarse-grained locally harmonic energy landscape. Nucleic Acids Res. 2007;35(Web Server issue):W477–W482. 17545201 10.1093/nar/gkm342 PMC1933200

[131] Yang Z, Mâjek P, Bahar I. Allosteric Transitions of Supramolecular Systems Explored by Network Models: Application to Chaperonin GroEL. PLoS Comput Biol. 2009;5(4):e1000360 10.1371/journal.pcbi.1000360 19381265 PMC2664929

[132] Prinz JH, Keller B, Noé F. Probing molecular kinetics with Markov models: metastable states, transition pathways and spectroscopic observables. Phys Chem Chem Phys. 2011;13(38):16912–16927. 10.1039/c1cp21258c 21858310

[133] Beauchamp KA, Bowman GR, Lane TJ, Maibaum L, Haque IS, Pande VS. MSMBuilder2: Modeling Conformational Dynamics at the Picosecond to Millisecond Scale. J Chem Theory Comput. 2011;7(10):3412–3419. 22125474 10.1021/ct200463m PMC3224091

[134] Ravindranathan KP, Gallicchio E, Levy RM. Conformational equilibria and free energy profiles for the allosteric transition of the ribose-binding protein. J Mol Biol. 2005;353(1):196–210. 16157349 10.1016/j.jmb.2005.08.009

[135] Pietrucci F, Marinelli F, Carloni P, Laio A. Substrate binding mechanism of HIV-1 protease from explicit-solvent atomistic simulations. J Amer Chem Soc. 2009;131(33):11811–11818. 19645490 10.1021/ja903045y

[136] Buch I, Giorgino T, De Fabritiis G. Complete reconstruction of an enzyme inhibitor binding process by molecular dynamics simulations. Proc Natl Acad Sci USA. 2011;108(25):10184–10189. 10.1073/pnas.1103547108 21646537 PMC3121846

[137] Feher VA, Durrant JD, Van Wart AT, Amaro RE. Computational approaches to mapping allosteric pathways. Curr Opinion Struct Biol. 2014;25:98–103. 10.1016/j.sbi.2014.02.004 PMC4040315 24667124

[138] Held M, Metzner P, Prinz JH, Noé F. Mechanisms of protein-ligand association and its modulation by protein mutations. Biophys J. 2011;100(3):701–710. 10.1016/j.bpj.2010.12.3699 21281585 PMC3030248

[139] Held M, Noé F. Calculating kinetics and pathways of protein-ligand association. Eur J Cell Biol. 2012;91(4):357–364. 10.1016/j.ejcb.2011.08.004 22018914

[140] Freddolino PL, Park S, Roux B, Schulten K. Force field bias in protein folding simulations. Biophys J. 2009;96(9):3772–3780. 10.1016/j.bpj.2009.02.033 19413983 PMC2711430

[141] Vitalini F, Mey AS, Noé F, Keller BG. Dynamic properties of force fields. J Chem Phys. 2015;142:084101 10.1063/1.4909549 25725706

[142] Sakae Y, Okamoto Y. Optimizations of protein force fields In: Liwo A, editor. Computational Methods to Study the Structure and Dynamics of Biomolecules and Biomolecular Processes. Berlin, Heidelberg: Springer-Verlag; 2014 p. 195–247.

[143] Clementi C. Coarse-grained models of protein folding: Toy-models or predictive tools? Curr Opinion Struct Biol. 2008;18:10–15. 10.1016/j.sbi.2007.10.005 18160277

[144] Kleinjung J, Fraternali F. Design and application of implicit solvent models in biomolecular simulations. Curr Opinion Struct Biol. 2014;25(100):126–134. 10.1016/j.sbi.2014.04.003 PMC4045398 24841242

[145] Dryga A, Warshel A. Renormalizing SMD: The Renormalization Approach and Its Use in Long Time Simulations and Accelerated PAU Calculations of Macromolecules,. J Phys Chem B. 2010;114(39):12720–12728. 10.1021/jp1056122 20836533 PMC2948080

[146] Chodera JD, Noé F. Markov state models of biomolecular conformational dynamics. Curr Opinion Struct Biol. 2014;25:135–144. 10.1016/j.sbi.2014.04.002 PMC4124001 24836551

[147] Shaw DE, Maragakis P, Lindorff-Larsen K, Piana S, Dror RO, Eastwood MP, et al Atomic-Level Characterization of the Structural Dynamics of Proteins. Science. 2010;330(6002):341–346. 10.1126/science.1187409 20947758

[148] Zagrovic B, Snow CD, Shirts MR, Pande VS. Simulation of folding of a small alpha-helical protein in atomistic detail using worldwide-distributed computing. J Mol Biol. 2002;323(5):927–937. 12417204 10.1016/s0022-2836(02)00997-x

[149] Wang K, Chodera JD, Yang Y, Shirts MR. Identifying ligand binding sites and poses using GPU-accelerated Hamiltonian replica exchange molecular dynamics. J Computer-Aided Mol Des. 2013;27(12):989–1007. 10.1007/s10822-013-9689-8 PMC4154199 24297454

[150] Ando T, Skolnick J. Sliding of Proteins Non-specifically Bound to DNA: Brownian Dynamics Studies with Coarse-Grained Protein and DNA Models. PLoS Comput Biol. 2014;10(12):e1003990 10.1371/journal.pcbi.1003990 25504215 PMC4263358

[151] Marklund EG, Mahmutovic A, Berg OG, Hammar P, van der Spoel D, Fange D, et al Transcription-factor binding and sliding on DNA studied using micro- and macroscopic models. Proc Natl Acad Sci USA. 2013;110(49):19796–19801. 10.1073/pnas.1307905110 24222688 PMC3856812

[152] Szöllösi D, Horváth T, Han K, Dokholyan NV, Tompa P, Kalmár L, et al Discrete molecular dynamics can predict helical prestructured motifs in disordered proteins. PLoS ONE. 2014;9(4):e95795 10.1371/journal.pone.0095795 24763499 PMC3998973

[153] Shukla D, Hernández CX, Weber JK, Pande VS. Markov State Models Provide Insights into Dynamic Modulation of Protein Function. Acc Chem Res. 2015;48(2):414–422. 10.1021/ar5002999 25625937 PMC4333613

[154] Koshland D. Application of a theory of enzyme specificity to protein synthesis. Proc Natl Acad Sci USA. 1958;44(2):98–104. 16590179 10.1073/pnas.44.2.98 PMC335371

[155] Bosshard HR. Molecular recognition by induced fit: how fit is the concept? Physiology. 2001;16:171–173. 10.1152/physiologyonline.2001.16.4.171 11479367

[156] Ma B, Kumar S, Tsai C, Nussinov R. Folding funnels and binding mechanisms. Protein Eng. 1999;12(9):713–720. 10506280 10.1093/protein/12.9.713

[157] Tsai C, Ma B, Nussinov R. Folding and binding cascades: shifts in energy landscapes. Proc Natl Acad Sci USA. 1999;96(18):9970–9972. 10468538 10.1073/pnas.96.18.9970 PMC33715

[158] Tsai C, Kumar S, Ma B, Nussinov R. Folding funnels, binding funnels, and protein function. Protein Sci. 1999;8(6):1181–1190. 10386868 10.1110/ps.8.6.1181 PMC2144348

[159] Monod J, Wyman J, Changeaux JP. On the nature of allosteric transitions: a plausible model. J Mol Biol. 1965;12:88–118. 14343300 10.1016/s0022-2836(65)80285-6

[160] Lange OF, Lakomek NA, Farés C, Schröder GF, Walter KF, Becker S, et al Recognition Dynamics Up to Microseconds Revealed from an RDC-Derived Ubiquitin Ensemble in Solution. Science. 2008;320(5882):1471–1475. 10.1126/science.1157092 18556554

[161] Csermely P, Palotai R, Nussinov R. Induced fit, conformational selection and independent dynamic segments: an extended view of binding events. Trends Biochem Sci. 2010;35(10):539–546. 10.1016/j.tibs.2010.04.009 20541943 PMC3018770

[162] Cui Q, Karplus M. Allostery and cooperativity revisited. Protein Sci. 2008;17(8):1295–1307. 10.1110/ps.03259908 18560010 PMC2492820

[163] Feixas F, Lindert S, Sinko W, McCammon JA. Exploring the role of receptor flexibility in structure-based drug discovery. Biophys Chem. 2014;186:31–45. 10.1016/j.bpc.2013.10.007 24332165 PMC4459653

[164] Ewing TJ, Makino S, Skillman AG, Kuntz ID. DOCK 4.0: search strategies for automated molecular docking of flexible molecule databases. J Comput Aided Mol Des. 2001;15(5):411–428. 11394736 10.1023/a:1011115820450

[165] Kramer B, Rarey M, Lengauer T. Evaluation of the FLEXX incremental construction algorithm for protein-ligand docking. Proteins: Struct Funct Bioinf. 1999;37(2):228–241. 10.1002/(sici)1097-0134(19991101)37:2<228::aid-prot8>3.0.co;2-8 10584068

[166] Wagener M, Vlieg J, Nabuurs SB. Flexible protein-ligand docking using the Fleksy protocol. J Comput Chem. 2012;33(12):1215–1217. 10.1002/jcc.22948 22371008

[167] Verdonk ML, Cole JC, Hartshorn MJ, Murray CW, Taylor RD. Improved protein-ligand docking using GOLD. Proteins: Struct Funct Bioinf. 2003;52(4):609–623. 10.1002/prot.10465 12910460

[168] Verdonk ML, Chessari G, Cole JC, Hartshorn MJ, Murray CW, Nissink JW, et al Modeling water molecules in protein-ligand docking using GOLD. J Med Chem. 2005;48(20):6504–6515. 16190776 10.1021/jm050543p

[169] Goodsell DS, Morris GM, Olson AJ. Automated docking of flexible ligands: applications of AutoDock. J Mol Recogn. 1996;9(1):1–5. 10.1002/(sici)1099-1352(199601)9:1<1::aid-jmr241>3.0.co;2-6 8723313

[170] Morris GM, Huey R, Lindstrom W, Sanner MF, Belew RK, Goodsell DS, et al AutoDock4 and AutoDockTools4: Automated docking with selective receptor flexibility. J Comput Chem. 2009;30(16):2785–2791. 10.1002/jcc.21256 19399780 PMC2760638

[171] Trott O, Olson AJ. AutoDock Vina: improving the speed and accuracy of docking with a new scoring function, efficient optimization, and multithreading. J Comput Chem. 2010;31(2):455–461. 10.1002/jcc.21334 19499576 PMC3041641

[172] Vass M, Tarcsay A, Keserü GM. Multiple ligand docking by Glide: implications for virtual second-site screening. J Comput Aided Mol Des. 2012;26(7):821–834. 10.1007/s10822-012-9578-6 22639078

[173] Davis IW, Baker D. RosettaLigand docking with full ligand and receptor flexibility. J Mol Biol. 2009;385(2):381–392. 10.1016/j.jmb.2008.11.010 19041878

[174] Meiler J, Baker D. ROSETTALIGAND: Protein-small molecule docking with full side-chain flexibility. Proteins: Struct Funct Bioinf. 2006;65(3):538–548. 10.1002/prot.21086 16972285

[175] Grosdidier A, Zoete V, Michielin O. SwissDock, a protein-small molecule docking web service based on EADock DSS. Nucleic Acids Res. 2011;39(Suppl 2):W270–W277. 21624888 10.1093/nar/gkr366 PMC3125772

[176] Spitzer R, Jain AN. Surflex-Dock: Docking benchmarks and real-world application. J Comput Aided Mol Des. 2012;26(6):687–699. 10.1007/s10822-011-9533-y 22569590 PMC3398190

[177] Chakraborty S. DOCLASP-Docking ligands to target proteins using spatial and electrostatic congruence extracted from a known holoenzyme and applying simple geometrical transformations. F1000Research. 2014;3. 10.12688/f1000research.5145.1 PMC4934513 27429737

[178] Ruiz-Carmona S, Alvarez-Garcia D, Foloppe N, Garmendia-Doval AB, Juhos S, et al rDock: A Fast, Versatile and Open Source Program for Docking Ligands to Proteins and Nucleic Acids. PLoS Comput Biol. 2014;10(4):e1003571 10.1371/journal.pcbi.1003571 24722481 PMC3983074

[179] Li H, Leung KS, Ballester PJ, Wong MH. istar: A web platform for large-scale protein-ligand docking. PLoS ONE. 2014;9(1):e85678 10.1371/journal.pone.0085678 24475049 PMC3901662

[180] Morris GM, Goodsell DS, Halliday RS, Huey R, Hart WE, Belew RK, et al Automated Docking Using a Lamarckian Genetic Algorithm and an Empirical Binding Free Energy Function. J Comput Chem. 1998;19(14):1639–1662.

[181] Huang D, Caflisch A. Library screening by fragment-based docking. J Mol Recogn. 2010;23(2):183–193. 10.1002/jmr.981 19718684

[182] Miranker A, Karplus M. Functionality maps of binding sites: a multiple copy simultaneous search method. Proteins. 1991;11(1):29–34. 1961699 10.1002/prot.340110104

[183] Dong J, Zhao H, Zhou T, Spiliotopoulos D, Rajendran C, Li XD, et al Structural Analysis of the Binding of Type I, I1/2, and II Inhibitors to Eph Tyrosine Kinases. ACS Med Chem Lett. 2015;6(1):79–83. 10.1021/ml500355x 25589935 PMC4291711

[184] Jones S, Thornton JM. Principles of protein-protein interactions. Proc Natl Acad Sci USA. 1996;93(1):13–20. 8552589 10.1073/pnas.93.1.13 PMC40170

[185] Conte LL, Chothia C, Janin J. The atomic structure of protein-protein recognition sites. J Mol Biol. 1999;285(5):2177–2198. 9925793 10.1006/jmbi.1998.2439

[186] Norel R, Retrey D, Wolfson HJ, Nussinov R. Examination of shape complementarity in docking of unbound proteins. Proteins. 1999;36(3):307–317. 10409824

[187] Betts MJ, Sternberg MJ. An analysis of conformational changes on protein-protein association: implications for predictive docking. Protein Eng. 1999;12(4):271–283. 10325397 10.1093/protein/12.4.271

[188] Decanniere K, Transue TR, Desmyter A, Maes D, Muyldermans S, Wyns L. Degenerate interfaces in antigen-antibody complexes. J Mol Biol. 2001;313(3):473–478. 11676532 10.1006/jmbi.2001.5075

[189] Ferrari AM, Wei BQ, Costantino L, Shoichet BK. Soft Docking and Multiple Receptor Conformations in Virtual Screening. J Med Chem. 2004;47(21):5076–5084. 15456251 10.1021/jm049756p PMC1413506

[190] Sherman W, Beard HS, R F. Use of an induced fit receptor structure in virtual screening. Chem Biol Drug Des. 2006;67(1):83–84. 16492153 10.1111/j.1747-0285.2005.00327.x

[191] Nabuurs SB, Wagener M, De Vlieg J. A flexible approach to induced fit docking. J Med Chem. 2007;50(26):6507–6518. 18031000 10.1021/jm070593p

[192] Ieong PU, Sorensen J, Vemu PL, Wong CW, Demir O, Williams NP, et al Progress towards automated Kepler scientific workflows for computer-aided drug discovery and molecular simulations. Procedia Computer Science. 2014;29:1745–1755. 29399238 10.1016/j.procs.2014.05.159 PMC5796787

[193] Amaro RE, Baron R, McCammon JA. An improved relaxed complex scheme for receptor flexibility in computer-aided drug design. J Comput Aided Mol Des. 2008;22(9):693–705. 10.1007/s10822-007-9159-2 18196463 PMC2516539

[194] B-Rao C, Subramanian J, Sharma SD. Managing protein flexibility in docking and its applications. Drug Discov today. 2009;14(7–8):394–400. 10.1016/j.drudis.2009.01.003 19185058

[195] Lexa KW, Carlson HA. Protein flexibility in docking and surface mapping. Q Rev Biophys. 2012;45(3):301–343. 10.1017/S0033583512000066 22569329 PMC4272345

[196] Kokh DB, Wade RC, Wenzel W. Receptor flexibility in small-molecule docking calculations. WIREs Comput Mol Sci. 2011;1(2):298–314.

[197] Leach AR. Ligand docking to proteins with discrete side-chain flexibility. J Mol Biol. 1994;235(1):345–356. 8289255 10.1016/s0022-2836(05)80038-5

[198] Tian S, Sun H, Pan P, Li D, Zhen X, Li Y, et al Assessing an ensemble docking-based virtual screening strategy for kinase targets by considering protein flexibility. J Chem Inf Model. 2014;54(10):2664–2679. 10.1021/ci500414b 25233367

[199] Sorensen J, Demir O, Swift RV, Feher VA, Amaro RE. Molecular docking to flexible targets. Method Mol Biol. 2015;1215:445–469. 10.1007/978-1-4939-1465-4_20 25330975

[200] Korb O, Olsson TS, Bowden SJ, Hall RJ, Verdonk ML, Liebeschuetz JW, et al Potential and limitations of ensemble docking. J Chem Inf Model. 2012;52(5):1262–1274. 10.1021/ci2005934 22482774

[201] Bohnuud T, Kozakov D, Vajda S. Evidence of conformational selection driving the formation of ligand binding sites in protein-protein interfaces. PLoS Comput Biol. 2014;10(10):e1003872 10.1371/journal.pcbi.1003872 25275445 PMC4183424

[202] Shan Y, Kim ET, Eastwood MP, Dror RO, Seeliger MA, Shaw DE. How does a drug moelcule find its target binding site? J Am Chem Soc. 2011;133(24):9181–9183. 10.1021/ja202726y 21545110 PMC3221467

[203] Kaus JW, Arrar M, McCammon JA. Accelerated Adaptive Integration Method. J Phys Chem B. 2014;118(19):5109–5118. 10.1021/jp502358y 24780083 PMC4025579

[204] Wu X, Brooks BR. Toward canonical ensemble distribution from self-guided Langevin dynamics simulation. J Chem Phys. 2011;134(13):134108 10.1063/1.3574397 21476744 PMC3087419

[205] Wu X, Hodoscek M, Brooks BR. Replica exchanging self-guided Langevin dynamics for efficient and accurate conformational sampling. J Chem Phys. 2012;137(4):044106 10.1063/1.4737094 22852596 PMC3416874

[206] Kaus JW, Pierce LT, Walker RC, McCammon JA. Improving the Efficiency of Free Energy Calculations in the Amber Molecular Dynamics Package. J Chem Theory Comput. 2013;9(9):4131–4139. 10.1021/ct400340s PMC3811123 24185531

[207] Grant BJ, McCammon JA, Gorfe AA. Conformational Selection in G-Proteins: Lessons from Ras and Rho. Biophys J. 2010;99(11):L87–L89. 10.1016/j.bpj.2010.10.020 21112273 PMC2998626

[208] Abankwa D, Hanzal-Bayer M, Ariotti N, Plowman SJ, Gorfe AA, Parton RG, et al A novel switch region regulates H-Ras membrane orientation and signal output. EMBO J. 2008;27(5):727–735. 10.1038/emboj.2008.10 18273062 PMC2265749

[209] Gu RX, Liu LA, Wang YH, Xu Q, Wei DQ. Structural comparison of the wild-type and drug-resistant mutants of the influenza A M2 proton channel by molecular dynamics simulations. J Phys Chem B. 2013;117(20):6042–6051. 10.1021/jp312396q 23594107

[210] Bozdaganyan ME, Orekhov PS, Bragazzi NL, Panatto D, Amicizia D, Pechkova E, et al Docking and Molecular Dynamics (MD) Simulations in Potential Drugs Discovery: An Application to Influenza Virus M2 Protein. American J Biochem Biotech. 2014;10(3):180–188.

[211] Waldmann M, Jirmann R, Hoelscher K, Wienke M, Niemeyer FC, Rehders D, et al A Nanomolar Multivalent Ligand as Entry Inhibitor of the Hemagglutinin of Avian Influenza. J Am Chem Soc. 2014;136(2):783–788. 10.1021/ja410918a 24377426

[212] Greenway KT, LeGresley EB, Pinto BM. The influence of 150-cavity binders on the dynamics of influenza A neuraminidases as revealed by molecular dynamics simulations and combined clustering. PLoS ONE. 2013;8(3):e59873 10.1371/journal.pone.0059873 23544106 PMC3609799

[213] Goh BC, Rynkiewicz MJ, Cafarella TR, White MR, Hartshorn KL, Allen K, et al Molecular mechanisms of inhibition of influenza by surfactant protein d revealed by large-scale molecular dynamics simulation. Biochemistry. 2013;52(47):8527–8538. 10.1021/bi4010683 24224757 PMC3927399

[214] Woods CJ, Shaw KE, Mulholland AJ. Combined Quantum Mechanics/Molecular Mechanics (QM/MM) Simulations for Protein-Ligand Complexes: Free Energies of Binding of Water Molecules in Influenza Neuraminidase. J Phys Chem B. 2014;119(3). 10.1021/jp506413j 25340313

[215] Ermak DL, McCammon J. Brownian dynamics with hydrodynamic interactions. J Chem Phys. 1978;69(4):1352–1360.

[216] ElSawy KM, Twarock R, Lane DP, Verma CS, Caves LS. Characterization of the ligand receptor encounter complex and its potential for in silico kinetics-based drug development. J Chem Theory Comput. 2011;8(1):314–321. 10.1021/ct200560w 26592892

[217] Mereghetti P, Wade RC. Atomic detail Brownian dynamics simulations of concentrated protein solutions with a mean field treatment of hydrodynamic interactions. J Phys Chem B. 2012;116(29):8523–8533. 10.1021/jp212532h 22594708

[218] ElSawy K, Verma CS, Joseph TL, Lane DP, Twarock R, Caves L. On the interaction mechanisms of a p53 peptide and nutlin with the MDM2 and MDMX proteins: a Brownian dynamics study. Cell Cycle. 2013;12(3):394–404. 10.4161/cc.23511 23324352 PMC3587439

[219] Frazier Z, Alber F. A Computational Approach to Increase Time Scales in Brownian Dynamics–Based Reaction-Diffusion Modeling. J Comput Biol. 2012;19(6):606–618. 10.1089/cmb.2012.0027 22697237 PMC3375646

[220] Beck M, Topf M, Frazier Z, Tjong H, Xu M, Zhang S, et al Exploring the spatial and temporal organization of a cell's proteome. J Struct Biol. 2011;173(3):483–496. 10.1016/j.jsb.2010.11.011 21094684 PMC3784337

[221] Tsai C, Nussinov R. A Unified View of "How Allostery Works". PLoS Comput Biol. 2014;10(2):e1003394 10.1371/journal.pcbi.1003394 24516370 PMC3916236

[222] Lockless SW, Ranganathan R. Evolutionarily conserved pathways of energetic connectivity in protein families. Science. 1999;286(5438):295–299. 10514373 10.1126/science.286.5438.295

[223] Daily MD, Upadhyaya TJ, Gray JJ. Contact rearrangements form coupled networks from local motions in allosteric proteins. Proteins. 2008;71(1):455–466. 17957766 10.1002/prot.21800 PMC5009369

[224] Kannan N, Vishveshwara S. Identification of side-chain clusters in protein structures by a graph spectral method. J Mol Biol. 1999;292(2):441–464. 10493887 10.1006/jmbi.1999.3058

[225] van den Bedem H, Bhabha G, Yang K, Wright PE, Fraser JS. Automated identification of functional dynamic contact networks from X-ray crystallography. Nat Methods. 2013;10(9):896–902. 10.1038/nmeth.2592 23913260 PMC3760795

[226] Boehr DD, Schnell JR, McElheny D, Bae SH, Duggan BM, Benkovic SJ, et al A distal mutation perturbs dynamic amino acid networks in dihydrofolate reductase. Biochemistry. 2013;52(27):4605–4619. 10.1021/bi400563c 23758161 PMC3838469

[227] Ferreiro DU, Hegler JA, Komives EA, Wolynes PG. Localizing frustration in native proteins and protein assemblies. Proc Natl Acad Sci USA. 2007;104(50):19819–19824. 18077414 10.1073/pnas.0709915104 PMC2148382

[228] Brooks B, Karplus M. Harmonic dynamics of proteins: normal modes and fluctuations in bovine pancreatic trypsin inhibitor. Proc Natl Acad Sci USA. 1983;80:6571–6575. 6579545 10.1073/pnas.80.21.6571 PMC391211

[229] Go N, Noguti T, Nishikawa T. Dynamics of a small globular protein in terms of low-frequency vibrational modes. Proc Natl Acad Sci USA. 1983;80(12):3696–3700. 6574507 10.1073/pnas.80.12.3696 PMC394117

[230] Levitt M, Sander C, Stern PS. The normal-modes of a protein-native bovine pancreatic trypsin-inhibitor. Intl J Quant Chem. 1983;Suppl 10:181–199.

[231] Garcia AE. Large-amplitude nonlinear motions in proteins. Phys Rev Lett. 1992;68(17):2696–2699. 10045464 10.1103/PhysRevLett.68.2696

[232] Amadei A, Linssen AB, Berendsen HJ. Essential dynamics of proteins. Proteins. 1993;17(4):412–425. 8108382 10.1002/prot.340170408

[233] Lange OF, Grubmüller H. Full correlation analysis of conformational protein dynamics. Proteins. 2008;70(4):1294–1312. 17876828 10.1002/prot.21618

[234] Girvan M, Newman MEJ. Community structure in social and biological networks. Proc Natl Acad Sci USA. 2002;99(12):7821–7826. 12060727 10.1073/pnas.122653799 PMC122977

[235] McClendon CL, Friedland G, Mobley DL, Amirkhani H, Jacobson MP. Quantifying correlations between allosteric sites in thermodynamic ensembles. J Chem Theory Comput. 2009;5(9):2486–2502. 20161451 10.1021/ct9001812 PMC2790287

[236] Sethi A, Eargle J, Black AA, Luthey-Schulten Z. Dynamical networks in tRNA:protein complexes. Proc Natl Acad Sci USA. 2009;106(16):6620–6625. 10.1073/pnas.0810961106 19351898 PMC2672494

[237] Eargle J, Luthey-Schulten Z. NetworkView: 3D display and analysis of protein RNA interaction networks. Bioinformatics. 2012;28(22):3000–3001. 10.1093/bioinformatics/bts546 22982572 PMC3496333

[238] Vanwart AT, Eargle J, Luthey-Schulten Z, Amaro RE. Exploring residue component contributions to dynamical network models of allostery. J Chem Theory Comput. 2012;8(8):2949–2961. 23139645 10.1021/ct300377a PMC3489502

[239] Kaya C, Armutlulu A, Ekesan S, Haliloglu T. MCPath: Monte Carlo path generation approach to predict likely allosteric pathways and functional residues. Nucleic Acids Res. 2013;41(Web Server Issue):W249–W255. 10.1093/nar/gkt284 23742907 PMC3692092

[240] Johnston JM, Wang H, Provasi D, Filizola M. Assessing the relative stability of dimer interfaces in G-protein coupled receptors. PLoS Comput Biol. 2012;8(8):e100264. 10.1371/journal.pcbi.1002649 PMC3420924 22916005

[241] Filizola M, Wang SX, Weinstein H. Dynamic models of G-protein coupled receptor dimers: indications of asymmetry in the rhodopsin dimer from molecular dynamics simulations in a POPC bilayer. J Comput Aided Mol Des. 2006;20(7–8):405–416. 17089205 10.1007/s10822-006-9053-3 PMC4076291

[242] Chen R, Li L, Weng Z. ZDock: an initial-stage protein-docking algorithm. Proteins: Struct Funct Bioinf. 2003;52(1):80–87. 10.1002/prot.10389 12784371

[243] Dominguez C, Boelens R, Bonvin AMJJ. HADDOCK: A protein-protein docking approach based on biochemical or biophysical information. J Am Chem Soc. 2003;125:1731–1737. 12580598 10.1021/ja026939x

[244] Comeau SR, Gatchell DW, Vajda S, Camacho CJ. ClusPro: a fully automated algorithm for protein-protein docking. Nucl Acids Res. 2004;32(S1):W96–W99. 15215358 10.1093/nar/gkh354 PMC441492

[245] Duhovny-Schneidman D, Inbar Y, Nussinov R, Wolfson HJ. PatchDock and SymmDock: servers for rigid and symmetric docking. Nucl Acids Res. 2005;33(S2):W363–W367. 15980490 10.1093/nar/gki481 PMC1160241

[246] Duhovny-Schneidman D, Inbar Y, Nussinov R, Wolfson HJ. Geometry based flexible and symmetric protein docking. Proteins: Struct Funct Bioinf. 2005;60(2):224–231. 10.1002/prot.20562 15981269

[247] Zacharias M. ATTRACT: protein-protein docking in CAPRI using a reduced protein model. Proteins: Struct Funct Bioinf. 2005;60(2):252–256. 10.1002/prot.20566 15981270

[248] Tovchigrechko A, Vakser IA. GRAMM-X public web server for protein-protein docking. Nucl Acids Res. 2006;34(Web Server issue):W310–4. 16845016 10.1093/nar/gkl206 PMC1538913

[249] Cheng TM, Blundell TL, Fernandez-Recio J. pyDock: electrostatics and desolvation for effective scoring of rigid-body protein-protein docking. Proteins. 2007;68(2):503–515. 17444519 10.1002/prot.21419

[250] Terashi G, Takeda-Shitaka M, Kanou K, Iwadate M, Takaya D, Umeyama H. The SKE-DOCK server and human teams based on a combined method of shape complementarity and free energy estimation. Proteins: Struct Funct Bioinf. 2007;69(4):866–887. 10.1002/prot.21772 17853449

[251] Lyskov S, Gray JJ. The RosettaDock server for local protein-protein docking. Nucl Acids Res. 2008;36(S2):W233–W238. 18442991 10.1093/nar/gkn216 PMC2447798

[252] Huang SY, Zou X. MDockPP: A hierarchical approach for protein-protein docking and its application to CAPRI rounds 15–19. Proteins: Struct Funct Bioinf. 2010;78(15):3096–3103. 10.1002/prot.22797 PMC2952710 20635420

[253] Mukherjee S, Zhang Y. Protein-Protein Complex Structure Predictions by Multimeric Threading and Template Recombination. Structure. 2011;19(7):955–966. 10.1016/j.str.2011.04.006 21742262 PMC3134792

[254] Guerler A, Govindarajoo B, Zhang Y. Mapping Monomeric Threading to Protein-Protein Structure Prediction. J Chem Inf and Model. 2013;53(3):717–725. 23413988 10.1021/ci300579r PMC4076494

[255] Cavalli A, Salvatella X, Dobson CM, Vendruscolo M. Protein structure determination from NMR chemical shifts. Proc Natl Acad Sci USA. 2007;104(23):9615–9620. 17535901 10.1073/pnas.0610313104 PMC1887584

[256] Lensink MF, Wodak SJ. Docking and scoring protein interactions: CAPRI 2009. Proteins: Struct Funct Bioinf. 2009;78(15):3073–3084. 10.1002/prot.22818 20806235

[257] Lensink MF, Wodak SJ. Blind predictions of protein interfaces by docking calculations in CAPRI. Proteins: Struct Funct Bioinf. 2010;78(15):3085–3095. 10.1002/prot.22850 20839234

[258] Mashiach E, Nussinov R, Wolfson HJ. FiberDock: Flexible induced-fit backbone refinement in molecular docking. Proteins: Struct Funct Bioinf. 2010;78(6):1503–1519. 10.1002/prot.22668 PMC4290165 20077569

[259] Pedotti M, Simonelli L, Livoti E, Varani L. Computational Docking of Antibody-Antigen Complexes, Opportunities and Pitfalls Illustrated by Influenza Hemagglutinin. Int J Mol Sci. 2011;12:226–251. 10.3390/ijms12010226 21339984 PMC3039950

[260] Gray JJ, Moughon S, Wang C, Schueler-Furman O, Kuhlman B, Rohl CA, et al Protein-protein docking with simultaneous optimization of rigid-body displacement and side-chain conformations. J Mol Biol. 2003;331(1):281–299. 12875852 10.1016/s0022-2836(03)00670-3

[261] Chaudhury S, Berrondo M, Weitzner BD, Muthu P, Bergman H, Gray JJ. Benchmarking and Analysis of Protein Docking Performance in Rosetta v3.2. PLoS ONE. 2011;6(8):e22477 10.1371/journal.pone.0022477 21829626 PMC3149062

[262] Ellingson SR, Miao Y, Baudry J, Smith JC. Multi-Conformer Ensemble Docking to Difficult Protein Targets. Phys Chem B. 2015;119(3):1026–1034. 10.1021/jp506511p 25198248

[263] Kozakov D, Beglov D, Bohnuud T, Mottarella SE, Xia B, Hall DR, et al How good is automated protein docking? Proteins: Struct Funct Bioinf. 2013;81(12):2159–2166. 10.1002/prot.24403 PMC3934018 23996272

[264] Moitessier N, Englebienne P, Lee D, Lawandi J, Corbeil CR. Towards the development of universal, fast and highly accurate docking/scoring methods: a long way to go. British J Pharmacology. 2009;153(S1):S7–S27. 10.1038/sj.bjp.0707515 PMC2268060 18037925

[265] Zhu H, Domingues FS, Sommer I, Lengauer T. NOXclass: prediction of protein-protein interaction types. BMC Bioinf. 2006;7:27. 10.1186/1471-2105-7-27 PMC1386716 16423290

[266] Moreira IS, Fernandes PA, Ramos MJ. Hot spots-A review of the protein-protein interface determinant amino-acid residues. Proteins. 2007;68(4):803–812. 17546660 10.1002/prot.21396

[267] Li N, Sun Z, Jiang F. Prediction of protein-protein binding site by using core interface residue and support vector machine. BMC Bioinf. 2008;9:553. 10.1186/1471-2105-9-553 PMC2627892 19102736

[268] Liu Q, J L. Propensity vectors of low-ASA residue pairs in the distinction of protein interactions. Proteins. 2009;78(3):589–602. 10.1002/prot.22583 19768686

[269] Hashmi I, Shehu A. idDock+: Integrating Machine Learning in Probabilistic Search for Protein-protein Docking. J Comp Biol. 2015;22(9):1–18. 10.1089/cmb.2015.0108 26222714

[270] Russel D, Lasker K, Webb B, J V, Tjioe E, Schneidman-Duhovny D, et al Putting the pieces together: integrative modeling platform software for structure determination of macromolecular assemblies. PLoS Biol. 2012;10(1):e1001244 10.1371/journal.pbio.1001244 22272186 PMC3260315

[271] Montalvao RW, Cavalli A, Salvatella X, Blundell TL, Vendruscolo M. Structure determination of protein-protein complexes using NMR chemical shifts: case of an endonuclease colicin-immunity protein complex. J Am Chem Soc. 2008;130(4):15990–1596. 18980319 10.1021/ja805258z

[272] Das R, André I, Shen Y, Wu Y, Lemak A, Bansal S, et al Simultaneous prediction of protein folding and docking at high resolution. Proc Natl Acad Sci USA. 2009;106(45):18978–18983. 10.1073/pnas.0904407106 19864631 PMC2770007

[273] Cavalli A, Montalvao RW, Vendruscolo M. Using Chemical Shifts to Determine Structural Changes in Proteins upon Complex Formation. Phys Chem B. 2011;115(30):9491–9494. 10.1021/jp202647q 21639128

[274] Alber F, Dokudovskaya S, Veenhoff LM, Zhang W, Kipper J, Devos D, et al Determining the architectures of macromolecular assemblies. Nature. 2007;450(7170):683–694. 18046405 10.1038/nature06404

[275] Fernandez-Martinez J, Phillips J, Sekedat MD, Diaz-Avalos R, Velazquez-Muriel J, Franke JD, et al Structure-function mapping of a heptameric module in the nuclear pore complex. J Cell Biol. 2012;196(4):419–434. 10.1083/jcb.201109008 22331846 PMC3283990

[276] Wang L, Yang MQ, Yang JY. Prediction of DNA-binding residues from protein sequence information using random forests. BMC Genomics. 2009;10(Suppl1):S1. 10.1186/1471-2164-10-S1-S1 PMC2709252 19594868

[277] Ofran Y, Mysore V, Rost B. Prediction of DNA-binding residues from sequence. Bioinformatics. 2007;23(13):347–353. 10.1093/bioinformatics/btm174 17646316

[278] Qin S, Zhou H. Structural Models of Protein-DNA Complexes Based on Interface Prediction and Docking. Curr Protein Pept Sci. 2011;12(6):531–539. 21787304 10.2174/138920311796957694 PMC3528948

[279] Roberts VA, Pique ME, Ten Eyck LF, Li S. Predicting protein–DNA interactions by full search computational docking. Proteins. 2013;8(12):2106–2118. 10.1002/prot.24395 PMC4045845 23966176

[280] van Dijk M, van Dijk AD, Hsu V, Boelens R, Bonvin AM. Information-driven protein-DNA docking using HADDOCK: it is a matter of flexibility. Nucleic Acids Res. 2013;34(11):3317–3325. 10.1093/nar/gkl412 PMC1500871 16820531

[281] Persikov AV, Wetzel JL, Rowland EF, Oakes BL, Xu DJ, Singh M, et al A systematic survey of the Cys2His2 zinc finger DNA-binding landscape. Nucleic Acids Res. 2015;43(3):1965–1984. 10.1093/nar/gku1395 25593323 PMC4330361

[282] Ghersi D, M S. Interaction-based discovery of functionally important genes in cancers. Nucleic Acids Res. 2014;42(3):e18 10.1093/nar/gkt1305 24362839 PMC3919581

[283] Ferré S, Navarro G, Casadó V, Cortés A, Mallol J, Canela EI, et al G protein-coupled receptor heteromers as new targets for drug development. Prog Mol Biol Transl Sci. 2011;91:41–54. 10.1016/S1877-1173(10)91002-8 PMC9361225 20691958

[284] Pietsch EC, Perchiniak E, Canutescu AA, Wang G, Dunbrack RL, Murphy ME. Oligomerization of BAK by p53 utilizes conserved residues of the p53 DNA binding domain. J Biol Chem. 2008;283(30):21294–21304. 10.1074/jbc.M710539200 18524770 PMC2475706

[285] Inbar Y, Benyamini H, Nussinov R, Wolfson HJ. Combinatorial docking approach for structure prediction of large proteins and multi-molecular assemblies. J Phys Biol. 2005;2:S156–S165. 10.1088/1478-3975/2/4/S10 16280621

[286] Inbar Y, Benyamini H, Nussinov R, Wolfson HJ. Prediction of multimolecular assemblies by multiple docking. J Mol Biol. 2005;349(2):435–447. 15890207 10.1016/j.jmb.2005.03.039

[287] Potluri S, Yan AK, Chou JJ, Donald BR, Bailey-Kellogg C. Structure determination of symmetric homo-oligomers by a complete search of symmetry configuration space, using NMR restraints and van der Waals packing. Proteins: Struct Funct Bioinf. 2006;65(1):203–219. 10.1002/prot.21091 16897780

[288] Sgourakis NG, Lange OF, DiMaio F, Andre I, Fitzkee NC, Rossi P, et al Determination of the Structures of Symmetric Protein Oligomers from NMR Chemical Shifts and Residual Dipolar Couplings. J Am Chem Soc. 2011;133(16):6288–6298. 10.1021/ja111318m 21466200 PMC3080108

[289] Martin JW, Yan AK, Bailey-Kellogg C, Zhou P, Donald BR. A geometric arrangement algorithm for structure determination of symmetric protein homo-oligomers from NOEs and RDCs. J Comp Biol. 2011;18(11):1507–1523. 10.1089/cmb.2011.0173 PMC3216109 22035328

[290] DiMaio F, Leaver-Fay A, Bradley P, Baker D, Andre I. Modeling Symmetric Macromolecular Structures in Rosetta3. PLoS ONE. 2011;6(6):e20450 10.1371/journal.pone.0020450 21731614 PMC3120754

[291] Pierce B, Tong W, Weng Z. M-ZDOCK: a grid-based approach for Cn symmetric multimer docking. Bioinformatics. 2004;21(8):1472–1478. 15613396 10.1093/bioinformatics/bti229

[292] Esquivel-Rodriguez J, Yang YD, Kihara D. Multi-LZerD: Multiple protein docking for asymmetric complexes. Proteins: Struct Funct Bioinf. 2012;80(7):1818–1833. 10.1002/prot.24079 PMC3370124 22488467

[293] Robustello P, Kai K, Cavalli A, Vendruscolo M. Using NMR Chemical Shifts as Structural Restraints in Molecular Dynamics Simulations of Proteins. Structure. 2010;18(8):923–933. 10.1016/j.str.2010.04.016 20696393

[294] Camilloni C, Cavalli A, Vendruscolo M. Assessment of the Use of NMR Chemical Shifts as Replica-Averaged Structural Restraints in Molecular Dynamics Simulations to Characterize the Dynamics of Proteins. Phys Chem B. 2012;117(6):1838–1843. 10.1021/jp3106666 23327201

[295] Kannan A, Camilloni C, Sahakyan AB, Cavalli A, Vendruscolo M. A Conformational Ensemble Derived Using NMR Methyl Chemical Shifts Reveals a Mechanical Clamping Transition That Gates the Binding of the HU Protein to DNA. J Am Chem Soc. 2014;136(6):2204–2207. 10.1021/ja4105396 24517490

[296] Pietrucci F, Mollica L, Blackledge M. Mapping the Native Conformational Ensemble of Proteins from a Combination of Simulations and Experiments: New Insight into the src-SH3 Domain. J Phys Chem Lett. 2013;4(11):1943–1948. 10.1021/jz4007806 26283131

[297] Wall ME, Van Benschoten AH, Sauter NK, Adams PD, Fraser JS, Terwilliger TC. Conformational dynamics of a crystalline protein from microsecond-scale molecular dynamics simulations and diffuse X-ray scattering. Proc Natl Acad Sci USA. 2014;111(50):17887–17892. 10.1073/pnas.1416744111 25453071 PMC4273327

[298] König G, Brooks BR. Correcting for the free energy costs of bond or angle constraints in molecular dynamics simulations. Biochim Biophys Acta. 2014;1850(5):932–942. 10.1016/j.bbagen.2014.09.001 25218695 PMC4339525

[299] Mustoe AM, Brooks CL, Al-Hashimi HM. Topological constraints are major determinants of tRNA tertiary structure and dynamics and provide basis for tertiary folding cooperativity. Nucleic Acids Res. 2014;42(18):11792–11804. 10.1093/nar/gku807 25217593 PMC4191394

[300] Wu X, Subramaniam S, Case DA, Wu KW, Brooks BR. Targeted conformational search with map-restrained self-guided Langevin dynamics: Application to flexible fitting into electron microscopic density maps. J Struct Biol. 2013;183(3):429–440. 10.1016/j.jsb.2013.07.006 23876978 PMC3785014

[301] Boomsma W, Ferkinghoff-Borg J, Lindorff-Larsen K. Combining Experiments and Simulations Using the Maximum Entropy Principle. PLoS Comput Biol. 2014;10(2):e1003406 10.1371/journal.pcbi.1003406 24586124 PMC3930489

[302] Granata D, Camilloni C, Vendruscolo M, Laio A. Characterization of the free-energy landscapes of proteins by NMR-guided metadynamics. Proc Natl Acad Sci USA. 2013;110(17):6817–6822. 10.1073/pnas.1218350110 23572592 PMC3637744

[303] Humphrey W, Dalke A, Schulten K. VMD—Visual Molecular Dynamics. J Mol Graph Model. 1996;14(1):33–38. http://www.ks.uiuc.edu/Research/vmd/. 10.1016/0263-7855(96)00018-5 8744570

[304] Cavalli A, Camilloni C, Vendruscolo M. Molecular dynamics simulations with replica-averaged structural restraints generate structural ensembles according to the maximum entropy principle. J Chem Phys. 2013;138(9):094112 10.1063/1.4793625 23485282

[305] Bonvin AM, Boelens R, Kaptein R. Time- and ensemble-averaged direct NOE restraints. J Biomol NMR. 1994;4(1):143–149. 10.1007/BF00178343 22911161

[306] Kessler H, Griesinger C, Lautz J, Mueller A, van Gunsteren WF, Berendsen HJC. Conformational dynamics detected by nuclear magnetic resonance NOE values and J coupling constants. J Am Chem Soc. 1998;110(11):3393–3396.

[307] Loquet A, Sgourakis NG, Gupta R, Giller K, Riedel D, Goosmann C, et al Atomic model of the type III secretion system needle. Nature. 2012;486(7402):276–279. 10.1038/nature11079 22699623 PMC3598588

[308] Pieper U, Schlessinger A, Kloppmann E, Chang GA, Chou JJ, Dumont ME, et al Coordinating the impact of structural genomics on the human *α*-helical transmembrane proteome. Nature Struct & Mol Biol. 2013;20(2):135–138. 23381628 10.1038/nsmb.2508 PMC3645303

[309] Torda AE, Scheek RM, van Gunsteren WF. Time-dependent distance restraints in molecular dynamics simulations. Chem Phys Lett. 1989;157(4):289–294.

[310] Vendruscolo M, Paci E, Dobson CM, Karplus M. Three key residues form a critical contact network in a protein folding transition state. Nature. 2001;409(6820):641–645. 11214326 10.1038/35054591

[311] Gong H, Y S, Rose GD. Building native protein conformation from NMR backbone chemical shifts using Monte Carlo fragment assembly. Protein Sci. 2007;16(8):1515–1521. 17656574 10.1110/ps.072988407 PMC2203357

[312] Richter B, Gsponer J, Várnai P, Salvatella X, Vendruscolo M. The MUMO (minimal under-restraining minimal over-restraining) method for the determination of native state ensembles of proteins. J Biomol NMR. 2007;37(2):117–135. 17225069 10.1007/s10858-006-9117-7

[313] Montalvao RW, De Simone A, Vendruscolo M. Determination of structural fluctuations of proteins from structure-based calculations of residual dipolar couplings. J Biomol NMR. 2012;53(4):281–292. 10.1007/s10858-012-9644-3 22729708

[314] Fu B, Kukic P, Camilloni C, Vendruscolo M. MD Simulations of Intrinsically Disordered Proteins with Replica-Averaged Chemical Shift Restraints. Biophys J. 2014;106(2):481a.

[315] Shen Y, Bax A. Homology modeling of larger proteins guided by chemical shifts. Nature Methods. 2015;12(8):747–750. 10.1038/nmeth.3437 26053889 PMC4521993

[316] Nasedkin A, Marcellini M, Religa TL, Freund SM, Menzel A, Fersht AR, et al Deconvoluting Protein (Un)folding Structural Ensembles Using X-Ray Scattering, Nuclear Magnetic Resonance Spectroscopy and Molecular Dynamics Simulation. PLoS ONE. 2015;10(5):e0125662 10.1371/journal.pone.0125662 25946337 PMC4422743

[317] de Groot BL, van Aalten DM, Scheek RM, Amadei A, Vriend G, Berendsen HJ. Prediction of protein conformational freedom from distance constraints. Proteins. 1997;29(2):240–251. 9329088 10.1002/(sici)1097-0134(199710)29:2<240::aid-prot11>3.0.co;2-o

[318] Wells SA. Geometric simulation of flexible motion in proteins. Methods Mol Biol. 2014;1084:173–192. 10.1007/978-1-62703-658-0_10 24061922

[319] Wells S, Menor S, Hespenheide B, Thorpe MF. Constrained geometric simulation of diffusive motion in proteins. J Phys Biol. 2005;2(4):127–136. 10.1088/1478-3975/2/4/S07 16280618

[320] Shehu A, Clementi C, Kavraki LE. Modeling Protein Conformational Ensembles: From Missing Loops to Equilibrium Fluctuations. Proteins: Struct Funct Bioinf. 2006;65(1):164–179. 10.1002/prot.21060 16917941

[321] Shehu A, Clementi C, Kavraki LE. Sampling Conformation Space to Model Equilibrium Fluctuations in Proteins. Algorithmica. 2007;48(4):303–327.

[322] Shehu A, Kavraki LE, Clementi C. On the Characterization of Protein Native State Ensembles. Biophys J. 2007;92(5):1503–1511. 17158570 10.1529/biophysj.106.094409 PMC1796840

[323] Chubunsky M, Hespenheide B, Jacobs DJ, Kuhn LA, Lei M, Menor S, et al Constraint Theory Applied to Proteins. Nanotech Res J. 2008;2(1):61–72.

[324] Clausen R, Shehu A. A Data-driven Evolutionary Algorithm for Mapping Multi-basin Protein Energy Landscapes. J Comp Biol. 2015;22(9):844–860. 10.1089/cmb.2015.0107 26203626

[325] Huang YPJ, Montellione GT. Structural biology: Proteins flex to function. Nature. 2005;438(7064):36–37. 16267540 10.1038/438036a

[326] Takala H, Björling A, Berntsson O, Lehtivuori H, Niebling S, Hoernke M, et al Signal amplification and transduction in phytochrome photosensors. Nature. 2014;509(7499):245–248. 10.1038/nature13310 24776794 PMC4015848

[327] Majek P, Weinstein H, Elber R. 13 In: Voth GA, editor. Pathways of conformational transitions in proteins. Taylor and Francis group; 2008 p. 185–203.

[328] Nury H, Poitevin F, Van Renterghem C, Changeux JP, Corringer PJ, Delarue M, et al One-microsecond molecular dynamics simulation of channel gating in a nicotinic receptor homologue. Proc Natl Acad Sci USA. 2010;107(14):6275–6280. 10.1073/pnas.1001832107 20308576 PMC2852019

[329] Calimet N, Simoes M, Changeux JP, Karplus M, Taly A, Cecchini M. A gating mechanism of pentameric ligand-gated ion channels. Proc Natl Acad Sci USA. 2013;110(42):E3987–E3996. 10.1073/pnas.1313785110 24043807 PMC3801054

[330] Ma J, Karplus M. Molecular switch in signal transduction: reaction paths of the conformational changes in ras p21. Proc Natl Acad Sci USA. 1997;94(22):11905–11910. 9342335 10.1073/pnas.94.22.11905 PMC23651

[331] Ovchinnikov V, Karplus M. Analysis and Elimination of a Bias in Targeted Molecular Dynamics Simulations of Conformational Transitions: Application to Calmodulin. J Phys Chem B. 2012;116(29):8584–8603. 10.1021/jp212634z 22409258 PMC3406239

[332] Hamelberg D, Mongan J, McCammon JA. Accelerated molecular dynamics: a promising and efficient simulation method for biomolecules. J Chem Phys. 2004;120(24):11919–11929. 15268227 10.1063/1.1755656

[333] Yao XQ, Grant BJ. Domain opening and dynamic coupling in the alpha subunit of heterotrimeric G proteins. Biophys J. 2013;105(2):L09–L10. 10.1016/j.bpj.2013.06.006 PMC3714883 23870276

[334] Beckstein O, Denning EJ, Perilla JR, Woolf TB. Zipping and unzipping of adenylate kinase: atomistic insights into the ensemble of open-closed transitions. J Mol Biol. 2009;394(1):160–176. 10.1016/j.jmb.2009.09.009 19751742 PMC2803350

[335] Zuckerman DM, Woolf TB. Efficient dynamic importance sampling of rare events in one dimension. Phys Rev E. 2000;63(1):016702. 10.1103/PhysRevE.63.016702 11304388

[336] Perilla JR, Beckstein O, Denning EJ, Woolf TB. Computing ensembles of transitions from stable states: dynamic importance sampling. J Comput Chem. 2011;32(2):196–209. 10.1002/jcc.21564 21132840 PMC6728917

[337] Krebs WG, Gerstein M. The morph server: a standardized system for analyzing and visualizing macromolecular motions in a database framework. Nucleic Acids Res. 2000;28(8):1665–1675. 10734184 10.1093/nar/28.8.1665 PMC102811

[338] Ye YZ, Godzik A. FATCAT: a web server for flexible structure comparison and structure similarity searching. Nucleic Acids Res. 2004;32(Web Server Issue):W582–W585. 15215455 10.1093/nar/gkh430 PMC441568

[339] Lindahl E, Azuara C, Koehl P, Delarue M. NOMAD-Ref: visualization, deformation and refinement of macromolecular structures based on all-atom normal mode analysis. Nucleic Acids Res. 2006;34(Web Server Issue):W52–W56. 16845062 10.1093/nar/gkl082 PMC1538881

[340] Weiss DR, Levitt M. Can morphing methods predict intermediate structures? J Mol Biol. 2009;385(2):665–674. 10.1016/j.jmb.2008.10.064 18996395 PMC2691871

[341] Kim KM, Jernigan RL, Chirikjian GS. Efficient generation of feasible pathways for protein conformationa transitions. Biophys J. 2002;83(3):1620–1630. 12202386 10.1016/S0006-3495(02)73931-3 PMC1302259

[342] Chu JW, Trout BL, Brooks CLI. A super-linear minimization scheme for the nudged elastic band method. J Chem Phys. 2003;119(24):12708–12717.

[343] Maragliano L, Fiser A, Vanden-Eijnden EJ, Ciccotti G. String method in collective variables: minimum free energy paths and isocommittor surfaces. J Chem Phys. 2006;125:024106. 10.1063/1.2212942 16848576

[344] Weinan E, Ren W, Vanden-Eijnden E. Simplified and improved string method for computing the minimum energy paths in barrier-crossing events. J Chem Phys. 2007;126:164103 17477585 10.1063/1.2720838

[345] Maragliano L, Vanden-Eijnden E. On-the-fly string method for minimum free energy paths calculation. Chem Phys Lett. 2007;446:182–190.

[346] Weinan E, Ren W, Vanden-Eijnden E. Finite temperature string methods for the study of rare events. J Phys Chem. 2005;109:6688–6693. 10.1021/jp0455430 16851751

[347] Ren W, Vanden-Eijnden E, Maragakis P, Weinan E. Transition pathways in complex systems: application of the finite-temperature string method to the alanine dipeptide. J Chem Phys. 2005;123:134109 16223277 10.1063/1.2013256

[348] Zhang BW, Jasnow D, Zuckermann DM. Efficient and verified simulation of a path ensemble for conformational change in a united-residue model of calmodulin. Proc Natl Acad Sci USA. 2007;104(46):18043–18048. 17984047 10.1073/pnas.0706349104 PMC2084293

[349] Adelman JL, Dale AL, Zwier MC, Bhatt D, Chong LT, Zuckerman DM, et al Simulations of the alternating access mechanism of the sodium symporter mhp1. Biophys J. 2011;101(10):2399–2407. 10.1016/j.bpj.2011.09.061 22098738 PMC3218348

[350] Huber GA, Kim S. Weighted-ensemble Brownian dynamics simulations for protein association reactions. Biophys J. 1996;70(1):97–110. 8770190 10.1016/S0006-3495(96)79552-8 PMC1224912

[351] Jaillet L, Corcho FJ, Perez JJ, Cortes J. Randomized tree construction algorithm to explore energy landscapes. J Comput Chem. 2011;32(16):3464–3474. 10.1002/jcc.21931 21919017

[352] Haspel N, Moll M, Baker ML, Chiu W, E KL. Tracing conformational changes in proteins. BMC Struct Biol. 2010;10(Suppl1):S1. 20487508 10.1186/1472-6807-10-S1-S1 PMC2873824

[353] Molloy K, Shehu A. Elucidating the Ensemble of Functionally-relevant Transitions in Protein Systems with a Robotics-inspired Method. BMC Struct Biol. 2013;13(Suppl 1):S8 10.1186/1472-6807-13-S1-S8 24565158 PMC3952944

[354] Molloy K, Clausen R, Shehu A. A Stochastic Roadmap Method to Model Protein Structural Transitions. Robotica. 2014;In press.

[355] Dill KA, MacCallum JL. The Protein-Folding Problem, 50 Years On. Science. 2012;338(6110):1042–1046. 10.1126/science.1219021 23180855

[356] Onuchic JN, Wolynes PG. Theory of protein folding. Curr Opinion Struct Biol. 2004;14:70–75. 10.1016/j.sbi.2004.01.009 15102452

[357] Best RB. Atomistic molecular simulations of protein folding. Curr Opinion Struct Biol. 2012;22(1):52–61. 10.1016/j.sbi.2011.12.001 22257762

[358] Shaw DE, et al. Millisecond-scale molecular dynamics simulations on anton. In: Conf on High Performance Computing, Networking, Storage and Analysis (SC09). New York, NY: ACM; 2009. p. 39.

[359] Hess B, Kutzner C, Van der Spoel D, Lindahl E. GROMACS4: algorithms for highly efficient, load-balanced, and scalable molecular simulation. J Chem Theory Comput. 2008;4(3):435–447. 10.1021/ct700301q 26620784

[360] Case DA, Darden TA, Cheatham TEI, Simmerling CL, Wang J, Duke RE, et al AMBER 14. University of California, San Francisco; 2014.

[361] Shirts M, Pande VJ. COMPUTING: Screen Savers of the World Unite! Science. 2000;290(5498):1903–1904. 17742054 10.1126/science.290.5498.1903

[362] Snow CD, Zagrovic B, Pande VS. The Trp-cage: folding kinetics and unfolded state topology via molecular dynamics simulations. J Am Chem Soc. 2002;124(49):14548–14549. 12465960 10.1021/ja028604l

[363] Singhal N, Snow CD, Pande VS. Using path sampling to build better Markovian state models: Predicting the folding rate and mechanism of a tryptophan zipper beta hairpin. J Chem Phys. 2004;121(1):415–425. 15260562 10.1063/1.1738647

[364] Jayachandran G, Vishal V, Pande VS. Using massively parallel simulation and Markovian models to study protein folding: Examining the dynamics of the villin headpiece. J Chem Phys. 2006;124(16):164902–164914. 16674165 10.1063/1.2186317

[365] Seibert MM, Patriksson AP, Hess B, van der Spoel D. Reproducible Polypeptide Folding and Structure Prediction using Molecular Dynamics Simulations. J Mol Biol. 2005;354(1):173–183. 16236315 10.1016/j.jmb.2005.09.030

[366] Sosnick TR, Hinshaw JR. How proteins fold. Science. 2011;334(6055):464–465. 10.1126/science.1214018 22034424

[367] Stigler J, Ziegler F, Gieseke A, Gebhardt JC, Rief M. The complex folding network of single calmodulin molecules. Science. 2011;28(6055):512–516. 10.1126/science.1207598 22034433

[368] Best RB, Hummer G, Eaton WA. Native contacts determine protein folding mechanisms in atomistic simulations. Proc Natl Acad Sci USA. 2013;110(44):17874–17879. 10.1073/pnas.1311599110 24128758 PMC3816414

[369] Maity H, Maity M, Krishna MG, Mayne L, Englander SW. Protein folding: the stepwise assembly of foldon units. Proc Natl Acad Sci USA. 2005;102(13):4741–4746. 15774579 10.1073/pnas.0501043102 PMC555724

[370] Bai Y, Sosnick TR, Mayne L, Englander SW. Protein folding intermediates: native state hydrogen exchange. Science. 1995;269(5221):192–197. 7618079 10.1126/science.7618079 PMC3432310

[371] Walters BT, Mayne L, Hinshaw JR, Sosnick TR, Englander SW. Folding of a large protein at high structural resolution. Proc Natl Acad Sci USA. 2013;110(47):18898–18903. 10.1073/pnas.1319482110 24191053 PMC3839771

[372] Beauchamp KA, Ensign DL, Das R, Pande VS. Quantitative comparison of villin headpiece subdomain simulations and triplet-triplet energy transfer experiments. Proc Natl Acad Sci USA. 2011;108(31):12734–12739. 10.1073/pnas.1010880108 21768345 PMC3150881

[373] Pande VS, Beachamp K, Bowman GR. Everything you wanted to know about Markov state models but were afraid to ask. Nature Methods. 2010;52(1):99–105. 10.1016/j.ymeth.2010.06.002 PMC2933958 20570730

[374] Prinz JH, Wu H, Sarich M, Keller B, Senne M, Held M, et al Markov models of molecular kinetics: generation and validation. J Chem Phys. 2011;134(17):174105 10.1063/1.3565032 21548671

[375] Da LT, Sheong FK, Silva DA, Huang X. Application of Markov State Models to simulate long timescale dynamics of biological macromolecules. Adv Exp Med Biol. 2014;805:29–66. 10.1007/978-3-319-02970-2_2 24446356

[376] Moult J, Fidelis K, Kryshtafovych A, Schwede T, Tramontano A. Critical assessment of methods of protein structure prediction (CASP)–round x. Proteins: Struct Funct Bioinf. 2014;82(S2):1–6. 10.1002/prot.24452 PMC4394854 24344053

[377] S oding J, Biegert A, Lupas AN. The HHpred interactive server for protein homology detection and structure prediction. Nucleic Acids Res. 2005;33(Web Server Issue):W244 15980461 10.1093/nar/gki408 PMC1160169

[378] Ko J, Park H, Seok C. GalaxyTBM: template-based modeling by building a reliable core and refining unreliable local regions. BMC Bioinf. 2012;13(1):198–206. 10.1186/1471-2105-13-198 PMC3462707 22883815

[379] Han KF, Baker D. Global properties of the mapping between local amino acid sequence and local structure in proteins. Proc Natl Acad Sci USA. 1996;93(12):5814–5818. 8650175 10.1073/pnas.93.12.5814 PMC39144

[380] Zhang Y. Progress and Challenges in protein structure prediction. Curr Opinion Struct Biol. 2008;18(3):342–348. 10.1016/j.sbi.2008.02.004 PMC2680823 18436442

[381] Xu J, Zhang Y. How significant is a protein structure similarity with TM-score = 0.5? Bioinformatics. 2010;26(7):889–895. 10.1093/bioinformatics/btq066 20164152 PMC2913670

[382] Zhang Y, Skolnick J. Scoring function for automated assessment of protein structure template quality. Proteins: Structure, Function, and Bioinformatics. 2004;57(4):702–710. 10.1002/prot.20264 15476259

[383] Roy A, Kucukural A, Zhang Y. I-TASSER: a unified platform for automated protein structure and function prediction. Nat Protoc. 2010;5(4):725–738. 10.1038/nprot.2010.5 20360767 PMC2849174

[384] DeBartolo J, Colubri A, Jha AK, Fitzgerald JE, Freed KF, Sosnick TR. Mimicking the folding pathway to improve homology-free protein structure prediction. Proc Natl Acad Sci USA. 2009;106(10):3734–3739. 10.1073/pnas.0811363106 19237560 PMC2656149

[385] Simoncini D, Berenger F, Shrestha R, Zhang KYJ. A Probabilistic Fragment-Based Protein Structure Prediction Algorithm. PLoS ONE. 2012;7(7):e38799 10.1371/journal.pone.0038799 22829868 PMC3400640

[386] Brunette TJ, Brock O. Guiding conformation space search with an all-atom energy potential. Proteins: Struct Funct Bioinf. 2009;73(4):958–972. 10.1002/prot.22123 PMC2752431 18536015

[387] Shehu A, Olson B. Guiding the Search for Native-like Protein Conformations with an Ab-initio Tree-based Exploration. Int J Robot Res. 2010;29(8):1106–1127.

[388] Olson B, Shehu A. Evolutionary-inspired probabilistic search for enhancing sampling of local minima in the protein energy surface. Proteome Sci. 2012;10(10):S5. 22759582 10.1186/1477-5956-10-S1-S5 PMC3380728

[389] Olson B, Hashmi I, Molloy K, Shehu A. Basin Hopping as a General and Versatile Optimization Framework for the Characterization of Biological Macromolecules. Advances in AI J. 2012;2012(674832).

[390] Olson B, Shehu A. Rapid Sampling of Local Minima in Protein Energy Surface and Effective Reduction through a Multi-objective Filter. Proteome Sci. 2013;11(Suppl1):S12. 24564970 10.1186/1477-5956-11-S1-S12 PMC3908317

[391] Olson B, Jong KAD, Shehu A. Off-Lattice Protein Structure Prediction with Homologous Crossover. In: Conf on Genetic and Evolutionary Computation (GECCO). New York, NY: ACM; 2013. p. 287–294.

[392] Olson B, Shehu A. Multi-Objective Stochastic Search for Sampling Local Minima in the Protein Energy Surface. In: ACM Conf on Bioinf and Comp Biol (BCB). Washington, D. C.; 2013. p. 430–439.

[393] Zhou J, W Y, Hu G, Shen B. Amino acid network for the discrimination of native protein structures from decoys. Curr Protein Pept Sci. 2014;15(6):522–528. 25059328 10.2174/1389203715666140724084709

[394] Uversky VN. Natively unfolded proteins: a point where biology waits for physics. Protein Sci. 2002;11:739–756. 11910019 10.1110/ps.4210102 PMC2373528

[395] Uversky VN. A decade and a half of protein intrinsic disorder: biology still waits for physics. Protein Sci. 2013;22:693–724. 10.1002/pro.2261 23553817 PMC3690711

[396] Monastyrskyy B, Kryshtafovych A, Moult J, Tramontano A, Fidelis K. Assessment of protein disorder region predictions in CASP10. Proteins: Struct Funct Bioinf. 2014;82(S2):127–137. 10.1002/prot.24391 PMC4406047 23946100

[397] Varadi M, Kosol S, Lebrun P, Valentini E, Blackledge M, Dunker AK, et al pE-DB: a database of structural ensembles of intrinsically disordered and of unfolded proteins. Nucleic Acids Res. 2014;42(Database issue):D326–335. 10.1093/nar/gkt960 24174539 PMC3964940

[398] Sickmeier M, Hamilton JA, LeGall T, Vacic V, Cortese MS, Tantos A, et al DisProt: the database of disordered proteins. Nucleic acids research. 2007;35(suppl 1):D786–D793. 17145717 10.1093/nar/gkl893 PMC1751543

[399] Fukuchi S, Sakamoto S, Nobe Y, Murakami SD, Amemiya T, Hosoda K, et al IDEAL: intrinsically disordered proteins with extensive annotations and literature. Nucleic acids research. 2012;40(D1):D507–D511. 22067451 10.1093/nar/gkr884 PMC3245138

[400] Rösner H, Papaleo E, Haxholm GW, Best RB, Kragelund BB, Lindorff-Larsen K. CECAM workshop on intrinsically disordered proteins: Connecting computation, physics, and biology ETH Zurich- September 2nd to 5th, 2013. Intrinsically Disordered Proteins. 2014;p. 1–5.

[401] Dunker AK, Babu MM, Barbar E, Blackledge M, Bondos SE, Dosztányi Z, et al What’s in a name? Why these proteins are intrinsically disordered. Intrinsically Disordered Proteins. 2013;1(1):e24157. 28516007 10.4161/idp.24157 PMC5424803

[402] van der Lee R, Buljan M, Lang B, Weatheritt RJ, Daughdrill GW, Dunker AK, et al Classification of intrinsically disordered regions and proteins. Chem Rev. 2014;114(13):6589–6631. 10.1021/cr400525m 24773235 PMC4095912

[403] Nussinov R, Wolynes PG. A second molecular biology revolution? The energy landscapes of biomolecular function. Phys Chem Chem Phys. 2014;16(14):6321–6322. 10.1039/c4cp90027h 24608340

[404] Csermely P, Sandhu KS, Hazai E, Hoksza Z, Kiss HJM, Miozzo F, et al Disordered proteins and network disorder in network descriptions of protein structure, dynamics and function. Hypotheses and a comprehensive review. Current Protein Peptide Sci. 2012;13(1):19–33. 10.2174/138920312799277992 22044146

[405] Uversky VN. Unusual biophysics of intrinsically disordered proteins. Biochim Biophys Acta. 2013;1834(5):932–951. 10.1016/j.bbapap.2012.12.008 23269364

[406] Luo Y, Ma B, Nussinov R, Wei G. Structural Insight into Tau Proteinâ€™s Paradox of Intrinsically Disordered Behavior, Self-Acetylation Activity, and Aggregation. J Phys Chem Lett. 2014;5(17):3026–3031. 25206938 10.1021/jz501457f PMC4154703

[407] Campen A, Williams RM, Brown CJ, Meng J, Uversky VN, Dunker AK. TOP-IDP-scale: a new amino acid scale measuring propensity for intrinsic disorder. Protein Pept Lett. 2008;15:956–963. 18991772 10.2174/092986608785849164 PMC2676888

[408] Jensen MR, Zweckstetter M, Huang J, Blackledge M. Exploring Free-Energy Landscapes of Intrinsically Disordered Proteins at Atomic Resolution Using NMR Spectroscopy. Chem Rev. 2014;114(13):6632–6660. 10.1021/cr400688u 24725176

[409] Deng X, Eickholt J, Cheng J. A comprehensive overview of computational protein disorder prediction methods. Mol Biosyst. 2012;8:114–121. 10.1039/c1mb05207a 21874190 PMC3633217

[410] Dosztányi Z, Mészáros B, Simon I. Bioinformatical approaches to characterize intrinsically disordered/unstructured proteins. Briefings in Bioinformatics. 2009;p. bbp061. 10.1093/bib/bbp061 20007729

[411] Zhou H, Pang X, Lu C. Rate constants and mechanisms of intrinsically disordered proteins binding to structured targets. Phys Chem Chem Phys. 2012;14(30):10466–10476. 10.1039/c2cp41196b 22744607 PMC3402904

[412] Zhu X, Lopes REM, Shim J, MacKerell AD. Intrinsic energy landscapes of amino acid side-chains. J Chem Inf Model. 2012;52(6):1559–1572. 10.1021/ci300079j 22582825 PMC3398815

[413] Palazzesi F, Prakash MK, Bonomi M, Barducci A. Accuracy of Current All-Atom Force-Fields in Modeling Protein Disordered States. J Chem Theory Comput. 2015;11(1):2–7. 10.1021/ct500718s 26574197

[414] Wang RY, Han Y, Krassovsky K, Sheffler W, Tyka M, Baker D. Modeling disordered regions in proteins using Rosetta. PLoS ONE. 2011;6(7):e22060 10.1371/journal.pone.0022060 21829444 PMC3146542

[415] Jensen MR, Blackledge M. Testing the validity of ensemble descriptions of intrinsically disordered proteins. Proc Natl Acad Sci USA. 2014;111(16):E1557–1558. 10.1073/pnas.1323876111 24639541 PMC4000822

[416] Lindorff-Larsen K, Trbovic N, Maragakis P, Piana S, Shaw DE. Structure and Dynamics of an Unfolded Protein Examined by Molecular Dynamics Simulation. J Am Chem Soc. 2012;134(8):3787–3791. 10.1021/ja209931w 22339051

[417] Parigi G, Rezaei-Ghaleh N, Giachetti A, Becker S, Fernandez C, Blackledge M, et al Long-Range Correlated Dynamics in Intrinsically Disordered Proteins. J Am Chem Soc. 2014;136(46):16201–16209. 10.1021/ja506820r 25331250

[418] Zhang W, Chen J. Replica exchange with guided annealing for accelerated sampling of disordered protein conformations. J Comput Chem. 2014;35(23):1682–1689. 10.1002/jcc.23675 24995857

[419] Fleishman SJ, Baker D. Role of the biomolecular energy gap in protein design, structure, and evolution. Cell. 2012;149(2):262–273. 10.1016/j.cell.2012.03.016 22500796

[420] Donald BR. Algorithms in structural molecular biology Cambridge, MA: MIT Press; 2011.

[421] Kuhlman B, Dantas G, Ireton GC, Varani G, Stoddard BL, Baker D. Design of a novel globular proteing fold with atomic-level accuracy. Science. 2003;302(5649):1364–1368. 14631033 10.1126/science.1089427

[422] Ashworth J, Havranek JJ, Duarte CM, Sussman D, Monnat RJ, Stoddard BL, et al omputational redesign of endonuclease DNA binding and cleavage specificity. Nature. 2006;441(7093):656–659. 16738662 10.1038/nature04818 PMC2999987

[423] Grigoryan G, Reinke AW, Keating AE. Design of protein-interaction specificity gives selective bZIP-binding peptides. Nature. 2009;458(7240):859–864. 10.1038/nature07885 19370028 PMC2748673

[424] Havranek JJ, Duarte CM, Baker D. A simple physical model for the prediction and design of protein-DNA interactions. J Mol Biol. 2004;344(1):59–70. 15504402 10.1016/j.jmb.2004.09.029

[425] Havranek JJ, Harbury PB. Automated design of specificity in molecular recognition. Nat Struct Biol. 2003;10(1):45–52. 12459719 10.1038/nsb877

[426] Fleishman SJ, Khare SD, Koga N, Baker D. Restricted sidechain plasticity in the structures of native proteins and complexes. Protein Sci. 2011;20(4):753–757. 10.1002/pro.604 21432939 PMC3081553

[427] Fleishman SJ, et al Community-wide assessment of protein-interface modeling suggests improvements to design methodology. J Mol Biol. 2011;414(2):289–302. 10.1016/j.jmb.2011.09.031 22001016 PMC3839241

[428] Jha RK, Leaver-Fay A, Yin S, Wu Y, Butterfoss GL, Szyperski T, et al Computational design of a PAK1 binding protein. J Mol Biol. 2010;400(2):257–270. 10.1016/j.jmb.2010.05.006 20460129 PMC2903434

[429] Karanicolas J, Corn JE, Chen I, Joachimiak LA, Dym O, Peck SH, et al A de novo protein binding pair by computational design and directed evolution. Molecular Cell. 2011;42(2):250–260. 10.1016/j.molcel.2011.03.010 21458342 PMC3102007

[430] Richter F, Leaver-Fay A, Khare SD, Bjelic S, Baker D. De novo enzyme design using Rosetta3. PLoS ONE. 2011;6(5):e19230 10.1371/journal.pone.0019230 21603656 PMC3095599

[431] Pabo C. Molecular technology. Designing proteins and peptides. Nature. 1983;301(5897):200–200. 6823300 10.1038/301200a0

[432] Janin J. Conformation of amino acid sidechains in proteins. J Mol Biol. 1978;125(3):357–386. 731698 10.1016/0022-2836(78)90408-4

[433] Kuhlman B, Baker D. Native protein sequences are close to optimal for their structures. Proc Natl Acad Sci USA. 2000;97(19):10383–10388. 10984534 10.1073/pnas.97.19.10383 PMC27033

[434] Dunbrack R. Rotamer libraries in the 21st century. Curr Opinion Struct Biol. 2002;12(4):431–440. 10.1016/s0959-440x(02)00344-5 12163064

[435] Dunbrack R, Cohen FE. Bayesian statistical analysis of protein side-chain rotamer preferences. Protein Sci. 1997;6(8):1661–1681. 9260279 10.1002/pro.5560060807 PMC2143774

[436] Dunbrack R, Karplus M. Backbone-dependent rotamer library for proteins. Application to side-chain prediction. J Mol Biol. 1993;230(2):543–574. 8464064 10.1006/jmbi.1993.1170

[437] Poole AM, Ranganathan R. Knowledge-based potentials in protein design. Curr Opinion Struct Biol. 2006;16(4):508–513. 10.1016/j.sbi.2006.06.013 16843652

[438] Pierce NA, Winfree E. Protein Design is NP-hard. Protein Eng Des Sel. 2002;15(10):779–782. 10.1093/protein/15.10.779 12468711

[439] Desmet J, de Maeyer M, Hazes B, Lasters I. The dead-end elimination theorem and its use in protein side-chain positioning. Nature. 1992;356:539–542. 21488406 10.1038/356539a0

[440] Gordon DB, Mayo SL. Branch-and-terminate: a combinatorial optimization algorithm for protein design. Structure. 1999;7(9):1089–1098. 10508778 10.1016/s0969-2126(99)80176-2

[441] Hong EJ, Lippow SM, Tidor B, T L. Rotamer optimization for protein design through MAP estimation and problem-size reduction. J Comput Chem. 2009;30(12):1923–1945. 10.1002/jcc.21188 19123203 PMC3495010

[442] Wernisch L, Hery S, Wodak SJ. Automatic protein design with all atom force-fields by exact and heuristic optimization. J Mol Biol. 2000;301(3):713–736. 10966779 10.1006/jmbi.2000.3984

[443] Althaus E, Kohlbacher O, Lenhof HP, Müller P. A combinatorial approach to protein docking with flexible side chains. J Comp Biol. 2002;9(4):597–612. 10.1089/106652702760277336 12323095

[444] Kingsford CL, Chazelle B, Singh M. Solving and analyzing side-chain positioning problems using linear and integer programming. Bioinformatics. 2005;21(7):1028–1039. 15546935 10.1093/bioinformatics/bti144

[445] Leaver-Fay A, Kuhlman B, Snoeyink J. An adaptive dynamic programming algorithm for the side chain placement problem. In: Pac Symp Biocomput; 2005. p. 16–27. 15759610

[446] Traoré S, Allouche D, André I, de Givry S, Katsirelos G, Schiex T, et al A new framework for computational protein design through cost function network optimization. Bioinformatics. 2013;29(17):2129–2136. 10.1093/bioinformatics/btt374 23842814

[447] Li Z, Yang Y, Zhan J, Dai L, Zhou Y. Energy Functions in De Novo Protein Design: Current Challenges and Future Prospects. Annu Rev Biophys. 2013;42:315–335. 10.1146/annurev-biophys-083012-130315 23451890 PMC3851009

[448] Arnold FH. Combinatorial and computational challenges for biocatalyst design. Nature. 2001;409(6817):253–257. 11196654 10.1038/35051731

[449] Gainza P, Roberts KE, Donald BR. Protein Design Using Continuous Rotamers. PLOS Comput Biol. 2012;8:1. 22279426 10.1371/journal.pcbi.1002335 PMC3257257

[450] Gainza P, Roberts KE, Georgiev I, Lilien RH, Keedy DA, Chen CY, et al OSPREY: protein design with ensembles, flexibility, and provable algorithms. Methods Enzymol. 2013;523:87 10.1016/B978-0-12-394292-0.00005-9 23422427 PMC3692370

[451] Shapovalov MV, Dunbrack RL. A smoothed backbone-dependent rotamer library for proteins derived from adaptive kernel density estimates and regressions. Structure. 2011;19(6):844–858. 10.1016/j.str.2011.03.019 21645855 PMC3118414

[452] Reeve SM, Gainza P, Frey KM, Georgiev I, Donald BR, Anderson AC. Protein design algorithms predict viable resistance to an experimental antifolate. Proc Natl Acad Sci USA. 2015;112(3):749–754. 10.1073/pnas.1411548112 25552560 PMC4311853

[453] Voigt CA, Gordon DB, Mayo SL. Trading accuracy for speed: A quantitative comparison of search algorithms in protein sequence design. J Mol Biol. 2000;299(3):789–803. 10835284 10.1006/jmbi.2000.3758

[454] Desjarlais JR, Handel TM. De novo design of the hydrophobic cores of proteins. Protein Sci. 1995;4(10):2006–2018. 8535237 10.1002/pro.5560041006 PMC2142989

[455] Raha K, Wollacott AM, Italia MJ, Desjarlais JR. Prediction of amino acid sequence from structure. Protein Sci. 2000;9(6):1106–1119. 10892804 10.1110/ps.9.6.1106 PMC2144664

[456] Allen BD, Mayo SL. Dramatic performance enhancements for the FASTER optimization algorithm. J Comput Chem. 2006;27(10):1071–1075. 16685715 10.1002/jcc.20420

[457] Desmet J, Spriet J, Lasters I. Fast and accurate side-chain topology and energy refinement (FASTER) as a new method for protein structure optimization. Proteins: Struct Funct Bioinf. 2002;48(1):31–43. 10.1002/prot.10131 12012335

[458] Liu Y, Kuhlman B. RosettaDesign Server for protein design. Nucleic Acids Res. 2006;34(Web Server Issue):W235–W238. 16845000 10.1093/nar/gkl163 PMC1538902

[459] Canutescu AA, Shelenkov AA, Dunbrack RL Jr. A graph-theory algorithm for rapid protein side chain prediction. Protein Sci. 2003;12(9):2001–2014. 12930999 10.1110/ps.03154503 PMC2323997

[460] Schueler-Furman O, Wang C, Bradley P, Misura K, Baker D. Progress in modeling of protein structures and interactions. Science. 2005;310(5748):638–642. 16254179 10.1126/science.1112160

[461] Skolnick J. In quest of an empirical potential for protein structure prediction. Curr Opinion Struct Biol. 2006;16(2):166–171. 10.1016/j.sbi.2006.02.004 16524716

[462] Humphris EL, Kortemme T. Prediction of protein-protein interface sequence diversity using flexible backbone computational protein design. Structure. 2008;16(12):1777–1788. 10.1016/j.str.2008.09.012 19081054

[463] Smith CA, Kortemme T. Backrub-like backbone simulation recapitulates natural protein conformational variability and improves mutant side-chain prediction. J Mol Biol. 2008;380(4):742–756. 10.1016/j.jmb.2008.05.023 18547585 PMC2603262

[464] Friedland GD, Linares AJ, Smith CA, Kortemme T. A simple model of backbone flexibility improves modeling of side-chain conformational variability. J Mol Biol. 2008;380(4):757–774. 10.1016/j.jmb.2008.05.006 18547586 PMC3574579

[465] Smith CA, Kortemme T. Predicting the tolerated sequences for proteins and protein interfaces using RosettaBackrub flexible backbone design. PLoS ONE. 2011;6(7):e20451 10.1371/journal.pone.0020451 21789164 PMC3138746

[466] Canutescu AA, Dunbrack RL. Cyclic Coordinate Descent: A Robotics Algorithm for Protein Loop Closure. Protein Sci. 2003;12(5):963–972. 12717019 10.1110/ps.0242703 PMC2323867

[467] Georgiev I, Keedy D, Richardson JS, Richardson DC, Donald BR. Algorithm for backrub motions in protein design. Bioinformatics. 2008;24(13):i196–204. 10.1093/bioinformatics/btn169 18586714 PMC2718647

[468] Keedy DA, Georgiev I, Triplett EB, Donald RR, Richardson DC, Richardson JS. The role of local backrub motions in evolved and designed mutations. PLoS Comput Biol. 2012;8(8):e1002629 10.1371/journal.pcbi.1002629 22876172 PMC3410847

[469] Murphy GS, Mills JL, Miley MJ, Machius M, Szyperski T, Kuhlman B. Increasing sequence diversity with flexible backbone protein design: the complete redesign of a protein hydrophobic core. Structure. 2012;20(6):1086–1096. 10.1016/j.str.2012.03.026 22632833 PMC3372604

[470] Ollikainen N, Kortemme T. Computational protein design quantifies structural constraints on amino acid covariation. PLoS Comput Biol. 2013;9(11):e1003313 10.1371/journal.pcbi.1003313 24244128 PMC3828131

[471] Jana B, Morcos F, Onuchic JN. From structure to function: the convergence of structure based models and co-evolutionary information. Phys Chem Chem Phys. 2014;16(14):6496–6507. 10.1039/c3cp55275f 24603809

[472] Sandler I, Zigdon N, Levy E, Aharoni A. The functional importance of co-evolving residues in proteins. Cell Mol Life Sci. 2014;71(4):673–682. 10.1007/s00018-013-1458-2 23995987 PMC11113390

[473] Kajan L, Hopf TA, Kalaus M, Marks DS, Rost B. FreeContact: fast and free software for protein contact prediction from residue co-evolution. BMC Bioinf. 2014;15:85. 10.1186/1471-2105-15-85 PMC3987048 24669753

[474] Ovchinnikov S, Kamisetty H, Baker D. Robust and accurate prediction of residue-residue interactions across protein interfaces using evolutionary information. eLife. 2014;3:e02030 10.7554/eLife.02030 24842992 PMC4034769

[475] Kosciolek T, Jones DT. De Novo Structure Prediction of Globular Proteins Aided by Sequence Variation-Derived Contacts. PLoS ONE. 2014;9(3):e92197 10.1371/journal.pone.0092197 24637808 PMC3956894

[476] Huang H, Ozkirimli E, Post CB. A comparison of three perturbation molecular dynamics methods for mModeling conformational transitions. J Chem Theory Comput. 2009;5(5):1301–1314. 20161143 10.1021/ct9000153 PMC2731424

[477] Malek R, Mousseau N. Dynamics of Lennard-Jones clusters: A characterization of the activation-relaxation technique. Phys Rev E. 2000;62(6):7723–7728. 10.1103/physreve.62.7723 11138044

[478] Earl DJ, Deem MW. Parallel tempering: theory, applications, and new perspectives. Phys Chem Chem Phys. 2005;7:3910–3916. 19810318 10.1039/b509983h

[479] Arora K, Brooks CLI. Large-scale allosteric conformational transitions of adenylate kinase appear to involve a population-shift mechanism. Proc Natl Acad Sci USA. 2007;104(47):18496–18501. 18000050 10.1073/pnas.0706443104 PMC2141805

[480] Zhang Y, Kihara D, Skolnick J. Local energy landscape flattening: parallel hyperbolic Monte Carlo sampling of protein folding. Proteins: Struct Funct Bioinf. 2002;48(2):192–201. 10.1002/prot.10141 12112688

[481] Huber T, Torda AE, van Gunsteren WF. Local elevation: a method for improving the searching properties of molecular dynamics simulation. J Comput Aided Mol Design. 1994;8(6):695–708. 10.1007/BF00124016 7738605

[482] Schulze BG, Grubmueller H, Evanseck JD. Functional significance of hierarchical tiers in carbonmonoxy myoglobin: conformational substates and transitions studied by conformational flooding simulations. J Am Chem Soc. 2000;122(36):8700–8711.

[483] Krueger P, Verheyden S, Declerck PJ, Engelborghs Y. Extending the capabilities of targeted molecular dynamics: simulation of a large conformational transition in plasminogen activator inhibitor 1. Protein Sci. 2001;10(4):798–808. 11274471 10.1110/ps.40401 PMC2373958

[484] Schlitter J, Engels M, Krueger P. Targeted molecular dynamics—a new approach for searching pathways of conformational transitions. Proteins: Struct Funct Bioinf. 1994;12(2):84–89. 10.1016/0263-7855(94)80072-3 7918256

[485] Mashi RJ, Jakobsson E. End-point targeted molecular dynamics: large-scale conformational changes in potassium channels. Biophys J. 2008;94(11):4307–4319. 10.1529/biophysj.107.118778 18310251 PMC2480670

[486] van der Vaart A, Karplus M. Minimum free energy pathways and free energy profiles for conformational transitions based on atomistic molecular dynamics simulations. J Chem Phys. 2007;126:164106 17477588 10.1063/1.2719697

[487] Ding F, Tsao D, Nie H, Dokholyan NV. Ab initio folding of proteins with all-atom discrete molecular dynamics. Structure. 2008;16(7):1010–1018. 10.1016/j.str.2008.03.013 18611374 PMC2533517

[488] Pan AC, Sezer D, Roux B. Finding transition pathways using the string method with swarms of trajectories. J Phys Chem B. 2008;112(11):3432–3440. 10.1021/jp0777059 18290641 PMC2757167

[489] Noé F, Doose S, Daidone I, Löllmann M, Sauer M, Chodera JD, et al Dynamical fingerprints for probing individual relaxation processes in biomolecular dynamics with simulations and kinetic experiments. Proc Natl Acad Sci USA. 2011;108(12):4822–4827. 10.1073/pnas.1004646108 21368203 PMC3064371

[490] Sim AYL, Minary P, Levitt M. Modeling nucleic acids. Curr Opinion Struct Biol. 2012;22(3):273–278. 10.1016/j.sbi.2012.03.012 PMC4028509 22538125

[491] Schneidman-Duhovny D, Pellarin R, Sali A. Uncertainty in integrative structural modeling. Curr Opinion Struct Biol. 2014;28(null):96–104. 10.1016/j.sbi.2014.08.001 PMC4252396 25173450

[492] Rohrdanz MA, Zheng W, Clementi C. Discovering mountain passes via torchlight: methods for the definition of reaction coordinates and pathways in complex macromolecular reactions. Annu Rev Phys Chem. 2013;64(null):295–316. 10.1146/annurev-physchem-040412-110006 23298245

[493] Kalyaanamoorthy S, Chen YPP. Modelling and enhanced molecular dynamics to steer structure-based drug discovery. Prog Biophys Mol Biol. 2014;114(3):123–136. 10.1016/j.pbiomolbio.2013.06.004 23827463

[494] Sponer J, Banas P, Jurecka P, Zgarbova M, Kuhrova P, Havrila M, et al Molecular Dynamics Simulations of Nucleic Acids. From Tetranucleotides to the Ribosome. Phys Chem Lett. 2014;5(10):1771–1782. 10.1021/jz500557y 26270382

[495] Biedermann J, Ullrich A, Schöneberg J, Noé F. ReaDDyMM: Fast Interacting Particle Reaction-Diffusion Simulations Using Graphical Processing Units. Biophys J. 2015;108(3):457–461. 10.1016/j.bpj.2014.12.025 25650912 PMC4317564

[496] Hamelberg D, Mongan J, McCammon JA. Accelerated molecular dynamics: a promising and efficient simulation method for biomolecules. J Chem Phys. 2004;120(24):11919–11929. 15268227 10.1063/1.1755656

[497] Wang Y, Harrison CB, Schulten K, McCammon JA. Implementation of accelerated molecular dynamics in NAMD. Computational science & discovery. 2011;4(1):015002. 21686063 10.1088/1749-4699/4/1/015002 PMC3115733

[498] Pierce LC, Salomon-Ferrer R, Augusto F de Oliveira C, McCammon JA, Walker RC. Routine access to millisecond time scale events with accelerated molecular dynamics. J Chem Theory Comput. 2012;8(9):2997–3002. 22984356 10.1021/ct300284c PMC3438784

[499] Miao Y, Nichols SE, Gasper PM, Metzger VT, McCammon JA. Activation and dynamic network of the M2 muscarinic receptor. Proc Natl Acad Sci USA. 2013;110(27):10982–10987. 10.1073/pnas.1309755110 23781107 PMC3703993

[500] Miao Y, Nichols SE, McCammon JA. Mapping of Allosteric Druggable Sites in Activation-Associated Conformers of the M2 Muscarinic Receptor. Chem Biol & Drug Design. 2014;83(2):237–246. 10.1111/cbdd.12233 PMC4012891 24112716

[501] Sinko W, Miao Y, de Oliveira CAF, McCammon JA. Population Based Reweighting of Scaled Molecular Dynamics. J Phys Chem B. 2013;117(42):12759–12768. 10.1021/jp401587e 23721224 PMC3808002

[502] Tribello GA, Ceriotti M, Parrinello M. A self-learning algorithm for biased molecular dynamics. Proc Natl Acad Sci USA. 2010;107(41):17509–17514. 10.1073/pnas.1011511107 20876135 PMC2955137

[503] Swendsen RH, Wang JS. Replica Monte Carlo simulation of spin glasses. Phys Rev Lett. 1986;57:2607–2609. 10033814 10.1103/PhysRevLett.57.2607

[504] Sugita Y, Okamoto Y. Replica-exchange molecular dynamics method for protein folding. Chem Phys Lett. 1999;314(1):141–151.

[505] Wang L, Friesner RA, Berne B. Replica exchange with solute scaling: A more efficient version of replica exchange with solute tempering (REST2). J Phys Chem B. 2011;115(30):9431–9438. 10.1021/jp204407d 21714551 PMC3172817

[506] van der Spoel D, Seibert MM. Protein Folding Kinetics and Thermodynamics from Atomistic Simulations. Phys Rev Lett. 2006;96(3):238102. 16803409 10.1103/PhysRevLett.96.238102

[507] Hess B, Scheek RM. Orientation restraints in molecular dynamics simulations using time and ensemble averaging. J Magn Reson. 2003;164(1):19–27. 12932451 10.1016/s1090-7807(03)00178-2

[508] De Simone A, Richter B, Salvatella X, Vendruscolo M. Toward an Accurate Determination of Free Energy Landscapes in Solution States of Proteins. J Am Chem Soc. 2009;131(11):3810–3811. 10.1021/ja8087295 19292482

[509] De Simone A, Montalvao RW, Vendruscolo M. Determination of Conformational Equilibria in Proteins Using Residual Dipolar Couplings. J Chem Theory Comput. 2011;7(12):4189–4195. 22180735 10.1021/ct200361b PMC3236604

[510] Allison JR, Hertig S, Missimer JH, Smith LJ, Steinmetz MO, Dolenc J. Probing the Structure and Dynamics of Proteins by Combining Molecular Dynamics Simulations and Experimental NMR Data. J Chem Theory Comput. 2012;8(10):3430–3444. 10.1021/ct300393b 26592994

[511] Markwick PRL, Nilges M. Computational approaches to the interpretation of NMR data for studying protein dynamics. J Chem Phys. 2012;396(2):124–134.

[512] Salmon L, Pierce L, Grimm A, Roldan JO, Mollica L, Jensen MR, et al Multi-Timescale Conformational Dynamics of the SH3 Domain of CD2-Associated Protein using NMR Spectroscopy and Accelerated Molecular Dynamics. Angew Chem Int Ed Engl. 2012;51(25):6103–6106. 10.1002/anie.201202026 22565613 PMC3541011

[513] Jaynes ET. Information Theory and Statistical Mechanics. Phys Rev. 1957;106(4):620–630.

[514] Roux B, Weare J. On the statistical equivalence of restrained-ensemble simulations with the maximum entropy method. J Chem Phys. 2013;138(8):084107 10.1063/1.4792208 23464140 PMC3598863

[515] Fu B, Sahakyan AB, Camilloni C, Tartaglia GG, Paci E, Caflisch A, et al ALMOST: An all atom molecular simulation toolkit for protein structure determination. J Comput Chem. 2014;35(14):1101–1105. 10.1002/jcc.23588 24676684

[516] Camilloni C, Cavalli A, Vendruscolo M. Replica-Averaged Metadynamics. J Chem Theory and Comput. 2013;9(12):5610–5617. 26592295 10.1021/ct4006272

[517] Torrie GM, Valleau JP. Monte Carlo free energy estimates using non-Boltzmann sampling: application to the sub-critical Lennard-Jones fluid. Chem Phys Lett. 1974;28(4):578–581.

[518] Roux B. The calculation of the potential of mean force using computer simulations. Computer Physics Communications. 1995;91(1):275–282.

[519] Bartels C, Karplus M. Multidimensional adaptive umbrella sampling: applications to main chain and side chain peptide conformations. J Comput Chem. 1997;18(12):1450–1462.

[520] Kumar S, Rosenberg JM, Bouzida D, Swendsen RH, Kollman PA. The weighted histogram analysis method for free-energy calculations on biomolecules. I. The method. J Comput Chem. 1992;13(8):1011–1021.

[521] Zhu F, Hummer G. Convergence and error estimation in free energy calculations using the weighted histogram analysis method. J Comput Chem. 2012;33(4):453–465. 10.1002/jcc.21989 22109354 PMC3271861

[522] Hub JS, de Groot BL, van der Spoel D. g_whams—A Free Weighted Histogram Analysis Implementation Including Robust Error and Autocorrelation Estimates. J Chem Theory Comput. 2010;6(12):3713–3720.

[523] Wojtas-Niziurski W, Meng Y, Roux B, Berneche S. Self-learning adaptive umbrella sampling method for the determination of free energy landscapes in multiple dimensions. J Chem Theory Comput. 2013;9(4):1885–1895. 23814508 10.1021/ct300978b PMC3694627

[524] Snyder R, Wang B, Roark M, Feller SE. Replica Exchange Umbrella Sampling Simulations Provide Insight into the Role of Docosahexaenoic Acid in Modulating the Stability of Transmembrane Proteins. Biophys J. 2014;106(2):16a.24411233

[525] Krivov SV, Karplus M. Hidden complexity of free energy surfaces for peptide (protein) folding. Proc Natl Acad Sci USA. 2004;101(41):14766–14770. 15466711 10.1073/pnas.0406234101 PMC522040

[526] Zheng W, Rohrdanz MA, Maggioni M, Clementi C. Polymer reversal rate calculated via locally scaled diffusion map. J Chem Phys. 2011;134(14):144109 10.1063/1.3575245 21495744

[527] Rohrdanz MA, Zheng W, Maggioni M, Clementi C. Determination of reaction coordinates via locally scaled diffusion map. J Chem Phys. 2011;134(12):124116 10.1063/1.3569857 21456654

[528] Zheng W, Rohrdanz MA, Clementi C. Rapid Exploration of Configuration Space with Diffusion-Map-Directed Molecular Dynamics. J Phys Chem B. 2013;117(42):12769–12776. 10.1021/jp401911h 23865517 PMC3808479

[529] Preto J, Clementi C. Fast recovery of free energy landscapes via diffusion-map-directed molecular dynamics. Phys Chem Chem Phys. 2014;16(36):19181–19191. 10.1039/c3cp54520b 24955434

[530] Becker OM, Karplus M. The topology of multidimensional potential energy surfaces: Theory and application to peptide structure and kinetics. J Chem Phys. 1997;106(4):1495–1517.

[531] Doye J, Miller M, Wales D. Evolution of the Potential Energy Surface with Size for Lennard-Jones Clusters. J Chem Phys. 1999;111(18):8417–8428.

[532] Krivov SV, Karplus M. Free energy disconnectivity graphs: Application to peptide models. J Chem Phys. 2002;117(23):10894–10903.

[533] Rao F, Caflisch A. The protein folding network. J Mol Biol. 2004;342(1):299–306. 15313625 10.1016/j.jmb.2004.06.063

[534] Muff S, Caflisch A. Kinetic analysis of molecular dynamics simulations reveals changes in the denatured state and switch of folding pathways upon single-point mutation of a *β*-sheet miniprotein. Proteins: Struct Funct Bioinf. 2008;70(4):1185–1195. 10.1002/prot.21565 17847092

[535] Caflisch A. Network and graph analyses of folding free energy surfaces. Curr Opinion Struct Biol. 2006;16(1):71–78. 10.1016/j.sbi.2006.01.002 16413772

[536] Krivov SV, Karplus M. Diffusive reaction dynamics on invariant free energy profiles. Proc Natl Acad Sci USA. 2008;105(37):13841–13846. 10.1073/pnas.0800228105 18772379 PMC2544541

[537] Zhou R. Free energy landscape of protein folding in water: explicit vs. implicit solvent. Proteins: Struct, Funct, Bioinf. 2003;53(2):148–161. 10.1002/prot.10483 14517967

[538] Barron LD, Hecht L, Wilson G. The lubricant of life: A proposal that solvent water promotes extremely fast conformational fluctuations in mobile heteropolypeptide structure. Biochemistry. 1997;36(43):13143–13147. 9376374 10.1021/bi971323j

[539] Singhal N, Pande VS. Error analysis and efficient sampling in Markovian state models for molecular dynamics. J Chem Phys. 2005;123(20):204909 16351319 10.1063/1.2116947

[540] Noe F, Fischer S. Transition networks for modeling the kinetics of conformational change in macromolecules. Curr Opinion Struct Biol. 2008;18:154–162. 10.1016/j.sbi.2008.01.008 18378442

[541] Pérez-Hernández G, Paul F, Giorgino T, De Fabritiis G, Noé F. Identification of slow molecular order parameters for Markov model construction. J Chem Phys. 2013;139(1):015102 10.1063/1.4811489 23822324

[542] Piana S, Lindorff-Larsen K, Shaw DE. Atomic-level description of ubiquitin folding. Proc Natl Acad Sci USA. 2013;110(15):5915–5920. 10.1073/pnas.1218321110 23503848 PMC3625349

[543] Weber JK, Jack RL, Pande VS. Emergence of glass-like behavior in Markov state models of protein folding dynamics. J Amer Chem Soc. 2013;135(15):5501–5504. 23540906 10.1021/ja4002663 PMC3677858

[544] Nj Deng, Dai W, Levy RM. How kinetics within the unfolded state affects protein folding: An analysis based on Markov state models and an ultra-long MD trajectory. J Phys Chem B. 2013;117(42):12787–12799. 10.1021/jp401962k 23705683 PMC3808496

[545] Voelz VA, Jaäger M, Yao S, Chen Y, Zhu L, Waldauer SA, et al Slow unfolded-state structuring in Acyl-CoA binding protein folding revealed by simulation and experiment. J Amer Chem Soc. 2012;134(30):12565–12577. 22747188 10.1021/ja302528z PMC3462454

[546] Weber M, Bujotzek A, Haag R. Quantifying the rebinding effect in multivalent chemical ligand-receptor systems. J Chem Phys. 2012;137(5):054111 10.1063/1.4739501 22894336

[547] Shukla D, Meng Y, Roux B, Pande VS. Activation pathway of Src kinase reveals intermediate states as targets for drug design. Nature Communications. 2014;5. 10.1038/ncomms4397 PMC4465921 24584478

[548] Kohlhoff KJ, Shukla D, Lawrenz M, Bowman GR, Konerding DE, Belov D, et al Cloud-based simulations on Google Exacycle reveal ligand modulation of GPCR activation pathways. Nature Chem. 2014;6(1):15–21. 24345941 10.1038/nchem.1821 PMC3923464

[549] Bowman GR, Geissler PL. Equilibrium fluctuations of a single folded protein reveal a multitude of potential cryptic allosteric sites. Proc Natl Acad Sci USA. 2012;109(29):11681–11686. 10.1073/pnas.1209309109 22753506 PMC3406870

[550] Lin YS, Bowman GR, Beauchamp KA, Pande VS. Investigating how peptide length and a pathogenic mutation modify the structural ensemble of amyloid beta monomer. Biophysic J. 2012;102(2):315–324. 10.1016/j.bpj.2011.12.002 PMC3260686 22339868

[551] Qiao Q, Bowman GR, Huang X. Dynamics of an intrinsically disordered protein reveal metastable conformations that potentially seed aggregation. J Amer Chem Soc. 2013;135(43):16092–16101. 24021023 10.1021/ja403147m

[552] Du WN, Bolhuis PG. Adaptive single replica multiple state transition interface sampling. J Chem Phys. 2013;139(4):044105 10.1063/1.4813777 23901958

[553] Noe F. Beating the millisecond barrier in molecular dynamics simulations. Biophys J. 2015;108:228–229. 10.1016/j.bpj.2014.11.3477 25606670 PMC4302188

[554] Laio A, Rodriguez-Fortea A, Gervasio FL, Ceccarelli M, Parrinello M. Assessing the accuracy of metadynamics. J Phys Chem B. 2005;109(14):6714–6721. 16851755 10.1021/jp045424k

[555] Barducci A, Bonomi M, Parrinello M. Metadynamics. Wiley Interdisciplinary Reviews: Computational Molecular Science. 2011;1(5):826–843.

[556] Bonomi M, Branduardi D, Bussi G, Camilloni C, Provasi D, Raiten P, et al PLUMED: a portable plugin for free-energy calculations wit h molecular dynamics. Comput Phys Communications. 2009;180(10):1961–1972.

[557] Bonomi M, Branduardi D, Gervasio FL, Parrinello M. The unfolded ensemble and folding mechanism of the C-terminal GB1 *β*-hairpin. J Am Chem Soc. 2008;130(42):13938–13944. 10.1021/ja803652f 18811160

[558] Piana S, Laio A, Marinelli F, Van Troys M, Bourry D, Ampe C, et al Predicting the effect of a point mutation on a protein fold: the villin and advillin headpieces and their Pro62Ala mutants. J Mol Biol. 2008;375(2):460–470. 18022635 10.1016/j.jmb.2007.10.020

[559] Berteotti A, Cavalli A, Branduardi D, Gervasio FL, Recanatini M, Parrinello M. Protein conformational transitions: the closure mechanism of a kinase explored by atomistic simulations. J Am Chem Soc. 2008;131(1):244–250. 10.1021/ja806846q 19067513

[560] Melis C, Bussi G, Lummis SC, Molteni C. Trans- cis Switching Mechanisms in Proline Analogues and Their Relevance for the Gating of the 5-HT3 Receptor. J Phys Chem B. 2009;113(35):12148–12153. 10.1021/jp9046962 19663504 PMC2733763

[561] Prakash MK, Barducci A, Parrinello M. Probing the mechanism of pH-induced large-scale conformational changes in dengue virus envelope protein using atomistic simulations. Biophys J. 2010;99(2):588–594. 10.1016/j.bpj.2010.04.024 20643078 PMC2905125

[562] Bocahut A, Bernad S, Sebban P, Sacquin-Mora S. Relating the diffusion of small ligands in human neuroglobin to its structural and mechanical properties. J Phys Chem B. 2009;113(50):16257–16267. 10.1021/jp906854x 19919085

[563] Nishihara Y, Hayashi S, Kato S. A search for ligand diffusion pathway in myoglobin using a metadynamics simulation. Chem Phys Lett. 2008;464(4):220–225.

[564] Provasi D, Bortolato A, Filizola M. Exploring molecular mechanisms of ligand recognition by opioid receptors with metadynamics. Biochemistry. 2009;48(42):10020–10029. 10.1021/bi901494n 19785461 PMC2764813

[565] Limongelli V, Bonomi M, Marinelli L, Gervasio FL, Cavalli A, Novellino E, et al Molecular basis of cyclooxygenase enzymes (COXs) selective inhibition. Proc Natl Acad Sci USA. 2010;107(12):5411–5416. 10.1073/pnas.0913377107 20215464 PMC2851773

[566] Masetti M, Cavalli A, Recanatini M, Gervasio FL. Exploring Complex Protein- Ligand Recognition Mechanisms with Coarse Metadynamics. J Phys Chem B. 2009;113(14):4807–4816. 10.1021/jp803936q 19298042

[567] Cavalli A, Spitaleri A, Saladino G, Gervasio FL. Investigating Drug–Target Association and Dissociation Mechanisms Using Metadynamics-Based Algorithms. Accounts Chem Res. 2014;. 10.1021/ar500356n 25496113

[568] Gur M, Madura JD, Bahar I. Global transitions of proteins explored by a multiscale hybrid methodology: application to adenylate kinase. Biophys J. 2013;105(7):1643–1652. 10.1016/j.bpj.2013.07.058 24094405 PMC3791301

[569] Atilgan A, Durell S, Jernigan R, Demirel M, Keskin O, Bahar I. Anisotropy of fluctuation dynamics of proteins with an elastic network model. Biophys J. 2001;80(1):505–515. 11159421 10.1016/S0006-3495(01)76033-X PMC1301252

[570] Das A, Gur M, Cheng MH, Jo S, Bahar I, Roux B. Exploring the Conformational Transitions of Biomolecular Systems Using a Simple Two-State Anisotropic Network Model. PLoS Comput Biol. 2014;10(4):e1003521 10.1371/journal.pcbi.1003521 24699246 PMC3974643

[571] Baron R. Fast Sampling of A-to-B Protein Global Conformational Transitions: From Galileo Galilei to Monte Carlo Anisotropic Network Modeling. Biophys J. 2013;105(7):1545–1546. 10.1016/j.bpj.2013.08.021 24094393 PMC3791302

[572] Suarez E, Lettieri S, Zwier MC, Stringer CA, Subramanian SR, Chong LT, et al Simultaneous computation of dynamical and equilibrium information using a weighted ensemble of trajectories. J Chem Theory Comput. 2014;10(7):2658–2667. 25246856 10.1021/ct401065r PMC4168800

[573] Rojnuckarin A, Kim S, Subramaniam S. Brownian dynamics simulations of protein folding: access to milliseconds time scale and beyond. Proc Natl Acad Sci USA. 1998;95(8):4288–4292. 9539729 10.1073/pnas.95.8.4288 PMC22481

[574] Bhatt D, Zuckerman DM. Beyond microscopic reversibility: Are observable nonequilibrium processes precisely reversible? J Chem Theory Comput. 2011;7(8):2520–2527. 21869866 10.1021/ct200086k PMC3159166

[575] Bhatt D, Zuckerman DM. Heterogeneous path ensembles for conformational transitions in semiatomistic models of adenylate kinase. J Chem Theory Comput. 2010;6(11):3527–3539. 21660120 10.1021/ct100406t PMC3108504

[576] Echols N, Milburn D, Gerstein M. MolMovDB: analysis and visualization of conformational change and structural flexibility. Nucleic Acids Res. 2003;31(1):478–482. 12520056 10.1093/nar/gkg104 PMC165551

[577] Flores S, Echols N, Milburn D, Hespenheide B, Keating K, Lu J, et al The Database of Macromolecular Motions: new features added at the decade mark. Nucleic Acids Res. 2006;34(suppl 1):D296–D301. 16381870 10.1093/nar/gkj046 PMC1347409

[578] Cecchini M, Houdusse A, Karplus M. Allosteric communication in myosin V: from small conformational changes to large directed movements. PLoS Comput Biol. 2008;4(8):e1000129 10.1371/journal.pcbi.1000129 18704171 PMC2497441

[579] Zhu F, Hummber G. Gating transition of pentameric ligand-gated ion channels. Biophys J. 2009;97(9):2456–2463. 10.1016/j.bpj.2009.08.020 19883588 PMC2770624

[580] Zimmermann MT, Kloczkowski A, Jernigan RL. MAVENs: motion analysis and visualization of elastic networks and structural ensembles. BMC Bioinf. 2011;12(1):264. 10.1186/1471-2105-12-264 PMC3213244 21711533

[581] Go N, Scheraga H. Analysis of contribution of internal vibrations to statistical weights of equilibrium conformations of macromolecules. J Chem Phys. 1969;51(11):4751–4767.

[582] Go N, Scheraga H. On the use of classical statistical-mechanics in treatment of polymer-chain conformation. Macromolecules. 1976;9(4):535–542.

[583] Flory PJ. Statistical thermodynamics of random networks. Proc Royal Soc. 1976;351(1666):351–380.

[584] Tirion MM. Large amplitude elastic motions in proteins from a single parameter, atomic analysis. Phys Rev Lett. 1996;77(9):1905–1908. 10063201 10.1103/PhysRevLett.77.1905

[585] Bahar I, Atilgan A, Erman B. Direct evaluation of thermal fluctuations in proteins using a single-parameter harmonic potential. Fold Des. 1997;2(3):173–181. 9218955 10.1016/S1359-0278(97)00024-2

[586] Micheletti C, Seno F, Banavar JR, Maritan A. Learning effective amino acid interactions through iterative stochastic techniques. Proteins. 2001;42(3):422–431. 11151013 10.1002/1097-0134(20010215)42:3<422::aid-prot120>3.0.co;2-2

[587] Halle B. Flexibility and packing in proteins. Proc Natl Acad Sci USA. 2002;99(3):1274–1279. 11818549 10.1073/pnas.032522499 PMC122180

[588] Haliloglu T, Bahar I, Erman B. Gaussian dynamics of folded proteins. Phys Rev Lett. 1997;79(16):3090–3093.

[589] Kundu S, Melton JS, Sorensen DC, Phillips GN. Dynamics of proteins in crystals: comparison of experiment with simple models. Biophys J. 2002;83(2):723–732. 12124259 10.1016/S0006-3495(02)75203-X PMC1302181

[590] Li G, Cui Q. Analysis of functional motions in Brownian molecular machines with an efficient block normal mode approach: myosin-II and Ca2+-ATPase. Biophys J. 2004;86(2):743–763. 14747312 10.1016/S0006-3495(04)74152-1 PMC1303924

[591] Ming D, Kong Y, Lambert MA, Huang Z, Ma J. How to describe protein motion without amino acid sequence and atomic coordinates. Proc Natl Acad Sci USA. 2002;99(13):8620–8625. 12084922 10.1073/pnas.082148899 PMC124334

[592] Delarue M, Sanejouand YH. Simplified normal mode analysis of conformational transitions in dna-dependent polymerases: the elastic network model. J Mol Biol. 2002;320(5):1011–1024. 12126621 10.1016/s0022-2836(02)00562-4

[593] Tama F, Valle M, Frank J, Brooks CL. Dynamic reorganization of the functionally active ribosome explored by normal mode analysis and cryo-electron microscopy. Proc Natl Acad Sci USA. 2003;100(16):9319–9323. 12878726 10.1073/pnas.1632476100 PMC170916

[594] Reuter N, Hinsen K, Lacapére JJ. Transconformations of the SERCA1 Ca-ATPase: a normal mode study. Biophys J. 2003;85(4):2186–2197. 14507684 10.1016/s0006-3495(03)74644-x PMC1303445

[595] Xu C, Tobi D, Bahar I. Allosteric changes in protein structure computed by a simple mechanical model: hemoglobin T–R2 transition. J Mol Biol. 2003;333(1):153–158. 14516750 10.1016/j.jmb.2003.08.027

[596] Tama F, Sanejouand YH. Conformational change of proteins arising from normal mode calculations. Protein Eng. 2001;14(1):1–6. 11287673 10.1093/protein/14.1.1

[597] Zheng W, Doniach S. A comparative study of motor-protein motions by using a simple elastic-network model. Proc Natl Acad Sci USA. 2003;100(23):13253–13258. 14585932 10.1073/pnas.2235686100 PMC263771

[598] Ikeguchi M, Ueno J, Sato M, Kidera A. Protein structural change upon ligand binding: linear response theory. Phys Rev Lett. 2005;94(7):078102 15783858 10.1103/PhysRevLett.94.078102

[599] Kim MK, Chirikjian GS, Jernigan RL. Elastic models of conformational transitions in macromolecules. J Mol Graph Model. 2002;21(2):151–160. 12398345 10.1016/s1093-3263(02)00143-2

[600] Tama F, Brooks CL. Diversity and identity of mechanical properties of icosahedral viral capsids studied with elastic network normal mode analysis. J Mol Biol. 2005;345(2):299–314. 15571723 10.1016/j.jmb.2004.10.054

[601] Tama F, Feig M, Liu J, Brooks CL, Taylor KA. The requirement for mechanical coupling between head and s2 domains in smooth muscle Myosin ATPase regulation and its implications for dimeric motor function. J Mol Biol. 2005;345(4):837–854. 15588830 10.1016/j.jmb.2004.10.084

[602] conformational transitions explored by mixed elastic network models P. Protein conformational transitions explored by mixed elastic network models. Proteins: Struct Funct Bioinf. 2007;69(1):43–57. 10.1002/prot.21465 17596847

[603] Maragakis P, Karplus M. Large amplitude conformational change in proteins explored with a plastic network model: adenylate kinase. J Mol Biol. 2005;352(4):807–822. 16139299 10.1016/j.jmb.2005.07.031

[604] Miayshita O, Onuchic JN, Wolynes PG. Nonlinear elasticity, proteinquakes, and the energy landscapes of functional transitions in proteins. Proc Natl Acad Sci USA. 2003;100(22):12570–12575. 14566052 10.1073/pnas.2135471100 PMC240658

[605] Miayshita O, Wolynes PG, Onuchic JN. Simple energy landscape model for the kinetics of functional transitions in proteins. J Phys Chem B. 2005;5(1959–1969):109. 10.1021/jp046736q 16851180

[606] Chu JW, Voth GA. Coarse-grained free energy functions for studying protein conformational changes: a double-well network model. Biophys J. 2007;93(11):3860–3871. 17704151 10.1529/biophysj.107.112060 PMC2084241

[607] Fischer S, Karplus M. Conjugate peak refinement: an algorithm for finding reaction paths and accurate transition states in systems with many degrees of freedom. Chem Phys Lett. 1992 6;194(3):252–261. Available from: 10.1016/0009-2614(92)85543-j.

[608] Weiss DR, Koehl P. Morphing Methods to Visualize Coarse-Grained Protein Dynamics In: Protein Dynamics. Springer; 2014 p. 271–282. 10.1007/978-1-62703-658-0_15 24061927

[609] Seo S, Kim MK. KOSMOS: a universal morph server for nucleic acids, proteins and their complexes. Nucleic Acids Res. 2012;40(Web Server issue):W531–W536. 10.1093/nar/gks525 22669912 PMC3394317

[610] Pratt L. A statistical method for identifying transition states in high dimensional problems. J Chem Phys. 1986;85(9):5045–5048.

[611] Dellago C, Bolhuis PG, Csajka FS, Chandler D. Transition path sampling and the calculation of rate constants. J Chem Phys. 1998;108(5):1964–1977.

[612] Woolf T. Path corrected functionals of stochastic trajectories: Towards relative free energy and reaction coordinate calculations. Chem Phys Lett. 1998;289(5–6):433–441.

[613] van Erp TS, Moroni D, Bolhuis PG. A novel path sampling method for the calculation of rate constants. J Chem Phys. 2003;118(17):7762–7774.

[614] Faradjian AK, Elber R. Computing time scales from reaction coordinates by milestoning. J Chem Phys. 2004;120(23):10880–10889. 15268118 10.1063/1.1738640

[615] Allen RJ, Warren PB, Ten Wolde PR. Sampling rare switching events in biochemical networks. Phys Rev Lett. 2005;94(1):018104 15698138 10.1103/PhysRevLett.94.018104

[616] Warmflash A, Bhimalapuram P, Dinner AR. Umbrella sampling for nonequilibrium processes. J Chem Phys. 2007;127(15):154112 17949137 10.1063/1.2784118

[617] Bolhuis PG, Chandler D, Dellago C, Geissler PL. Transition path sampling: throwing ropes over mountain passes in the dark. Annu Rev Phys Chem. 2002;53:291–318. 11972010 10.1146/annurev.physchem.53.082301.113146

[618] Dellago C, Bolhuis PG. Transition path sampling and other advanced simulation techniques for rare events In: Holm C, Kremer K, editors. Advanced Computer Simulation Approaches for Soft Matter Sciences III. vol. 221 of Advances in Polymer Science Springer Berlin Heidelberg; 2009 p. 167–233.

[619] Vanden-Eijnden EW. Towards a theory of transition paths. J Stat Phys. 2006;123(3):503–523.

[620] Elber R, Karplus M. A method for determining reaction paths in large molecules: Application to myoglobin. Chem Phys Lett. 1987;139(5):375–380.

[621] Henkelmann G, Jónsson H. Improved tangent estimate in the nudged elastic band method for finding minimum energy paths and saddle points. J Chem Phys. 2000;113:9978–9985.

[622] Weinan E, Ren W, Vanden-Eijnden E. String method for the study of rare events. Phys Rev B. 2002;66:052301. 10.1021/jp0455430 16851751

[623] Bohner MU, Zeman J, Smiatek J, Arnold A, Kästner J. Nudged-elastic band used to find reaction coordinates based on the free energy. J Chem Phys. 2014;140(7):074109 10.1063/1.4865220 24559340

[624] Jónsson H, Mills G, Jacobsen KW. Nudged Elastic Band Method for Finding Minimum Energy Paths of Transitions In: Berne BJ, Ciccotti G, Coker DF, editors. Classical and Quantum Dynamics in Condensed Phase Simulations. Singapore: World Scientific; 1998 p. 385–404.

[625] Olender R, Elber R. Yet another look at the steepest descent path. J Mol Struct THEOCHEM. 1997;398–399:63–71.

[626] Crehuet R, Field MJ. A temperature-dependent nudged-elastic-band algorithm. J Chem Phys. 2003;118(21):9653–9571.

[627] Ren W, Vanden-Eijnden E. Finite temperature string method for the study of rare events. J Phys Chem B. 2005;109(14):6688–6693. 16851751 10.1021/jp0455430

[628] Pan AC, Weinreich TM, Shan Y, Scarpazza DP, Shaw DE. Assessing the accuracy of two enhanced sampling methods using EGFR kinase transition pathways: the influence of collective variable choice. J Chem Theory and Comput. 2014;10(7):2860–2865. 26586510 10.1021/ct500223p

[629] Ovchinnikov V, Karplus M. Investigations of *α*-helix—*β*-sheet transition pathways in a miniprotein using the finite-temperature string method. J Chem Phys. 2014;140(17):175103 10.1063/1.4871685 24811667 PMC4032436

[630] Ovchinnikov V, Karplus M, Vanden-Eijnden E. Free energy of conformational transition paths in biomolecules: The string method and its application to myosin VI. J Chem Phys. 2011;134(8):085103 10.1063/1.3544209 21361558 PMC3060930

[631] Stober ST, Abrams CF. Energetics and mechanism of the normal-to-amyloidogenic isomerization of *β*2-microglobulin: On-the-fly string method calculations. J Phys Chem B. 2012;116(31):9371–9375. 10.1021/jp304805v 22793795 PMC3437544

[632] Matsunaga Y, Fujisaki H, Terada T, Kidera A. Conformational Transition Pathways of Adenylate Kinase Explored by the String Method. Biophys J. 2012;102(3):733a.

[633] Kumari M, Kozmon S, Kulhanek P, Stepan J, Tvaroska I, Koča J. Exploring Reaction Pathways for O-GlcNAc Transferase Catalysis. A String Method Study. J Phys Chem B. 2015;. 10.1021/jp511235f 25731954

[634] Fajer M, Meng Y, Roux B. Simulation of the Conformational Transition Pathway for the Activation of Full-Length C-Src Kinase using the String Method. Biophys J. 2014;106(2):639a–640a.24507604

[635] Ovchinnikov V, Cecchini M, Vanden-Eijnden E, Karplus M. Free energy of conformational transition paths in biomolecules: The string method and its application to myosin VI. Biophys J. 2011;101(10):2436–2444. 10.1016/j.bpj.2011.09.044 21361558 PMC3060930

[636] Adelman JL, Grabe M. Simulating rare events using a weighted ensemble-based string method. J Chem Phys. 2013;138(4):044105 10.1063/1.4773892 23387566 PMC3568092

[637] Gan W, Yang S, Roux B. Atomistic view of the conformational activation of Src kinase using the string method with swarms-of-trajectories. Biophys J. 2009;97(4):L8–L10. 10.1016/j.bpj.2009.06.016 19686639 PMC2726321

[638] Maragliano L, Roux B, Vanden-Eijnden E. Comparison between Mean Forces and Swarms-of-Trajectories String Methods. J Chem Theory Comput. 2014;10(2):524–533. 10.1021/ct400606c 26580029 PMC6980172

[639] Sanchez-Martinez M, Field M, Crehuet R. Enzymatic Minimum Free Energy Path Calculations Using Swarms of Trajectories. J Phys Chem B. Epub ahead of print 2014 Oct 17. 10.1021/jp506593t 25286154

[640] Peters B, Heyden A, Bell AT, Chakraborty A. A growing string method for determining transition states: comparison to the nudged elastic band and string methods. J Chem Phys. 2004;120(17):7877–7886. 15267702 10.1063/1.1691018

[641] Quapp W. A growing string method for the reaction pathway defined by a Newton trajectory. J Chem Phys. 2005;122(17):174106/1–174106/11. 15910022 10.1063/1.1885467

[642] Goodrow A, Bell AT, Head-Gordon M. Development and application of a hybrid method involving interpolation and ab initio calculations for the determination of transition states. J Chem Phys. 2008;129(17):174109/1–174109/12. 19045335 10.1063/1.2992618

[643] Goodrow A, Bell AT, Head-Gordon M. Transition state-finding strategies for use with the growing string method. J Chem Phys. 2009;130(24):244108/1–244108/14. 19566143 10.1063/1.3156312

[644] Goodrow A, Bell AT, Head-Gordon M. A strategy for obtaining a more accurate transition state estimate using the growing string method. Chem Phys Lett. 2010;484(4–6):392–398.

[645] Behn A, Zimmerman PM, Bell AT, Head-Gordon M. Efficient exploration of reaction paths via a freezing string method. J Chem Phys. 2011;135(22):224108–224116. 10.1063/1.3664901 22168681

[646] Mallikarjun Sharada S, Zimmerman PM, Bell AT, Head-Gordon M. Automated transition state searches without evaluating the Hessian. J Chem Theory Comput. 2012;8(12):5166–5174. 10.1021/ct300659d 26593206

[647] De Jong KA. Evolutionary Computation: A Unified Approach. Cambridge, MA: MIT Press; 2006.

[648] Unger R. The Genetic Algorithm Approach to Protein Structure Prediction. Structure and Bonding. 2004;110:153–175.

[649] Wales DJ, Doye JPK. Global Optimization by Basin-Hopping and the Lowest Energy Structures of Lennard-Jones Clusters Containing up to 110 Atoms. J Phys Chem A. 1997;101(28):5111–5116.

[650] Shehu A. Probabilistic Search and Optimization for Protein Energy Landscapes In: Aluru S, Singh A, editors. Handbook of Computational Molecular Biology. Chapman & Hall/CRC Computer & Information Science Series; 2013.

[651] Shehu A. omputer-Aided Drug Discovery In: Zhang W, editor. Methods in Pharmacology and Toxicology. Springer Verlag; 2015.

[652] Hoque M, Chetty M, Sattar A. Genetic Algorithm in Ab Initio Protein Structure Prediction Using Low Resolution Model: A Review. Biomed Data and Applications. 2009;p. 317–342.

[653] Dotu II, Cebrián MM, Van Hentenryck PP, Clote PP. On lattice protein structure prediction revisited. IEEE/ACM Trans Comput Biol Bioinf. 2011 11;8(6):1620–1632. 10.1109/TCBB.2011.41 21358007

[654] Prentiss MC, Wales DJ, Wolynes PG. Protein structure prediction using basin-hopping. J Chem Phys. 2008;128(22):225106–225106. 10.1063/1.2929833 18554063 PMC2674628

[655] Olson B, Shehu A. Multi-Objective Optimization Techniques for Conformational Sampling in Template-Free Protein Structure Prediction. In: Intl Conf on Bioinf and Comp Biol (BICoB). Las Vegas, NV; 2014.

[656] Verma A, Schug A, Lee KH, Wenzel W. Basin hopping simulations for all-atom protein folding. J Chem Phys. 2006;124(4):044515 16460193 10.1063/1.2138030

[657] Baldwin JM. A new factor in evolution. American Naturalists. 1896;p. 441–451.

[658] Rusu M, Birmanns S. Evolutionary tabu search strategies for the simultaneous registration of multiple atomic structures in cryo-EM reconstructions. J Struct Biol. 2010;170(1):164–171. 10.1016/j.jsb.2009.12.028 20056148 PMC2872094

[659] Rusu M, Wriggers W. Evolutionary bidirectional expansion for the tracing of alpha helices in cryo-electron microscopy reconstructions. J Struct Biol. 2012;177(2):410–419. 10.1016/j.jsb.2011.11.029 22155667 PMC3288247

[660] Clausen R, Ma B, Nussinov R, Shehu A. Mapping the Conformation Space of Wildtype and Mutant H-Ras with a Memetic, Cellular, and Multiscale Evolutionary Algorithm. PLoS Comput Biol. 2015;11(9):e1004470 10.1371/journal.pcbi.1004470 26325505 PMC4556523

[661] Kim D, Blum B, Bradley P, Baker D. Sampling bottlenecks in de novo protein structure prediction. J Mol Biol. 2009;393(1):249–60. 10.1016/j.jmb.2009.07.063 19646450 PMC2760740

[662] Choset H, et al Principles of Robot Motion: Theory, Algorithms, and Implementations. 1st ed. Cambridge, MA: MIT Press; 2005.

[663] Kavraki LE, Svetska P, Latombe JC, Overmars M. Probabilistic roadmaps for path planning in high-dimensional configuration spaces. IEEE Trans Robot Autom. 1996;12(4):566–580.

[664] Amato NM, Dill KA, Song G. Using motion planning to map protein folding landscapes and analyze folding kinetics of known native structures. J Comp Biol. 2002;10(3–4):239–255. 10.1089/10665270360688002 12935327

[665] Song G, Amato NM. A Motion Planning Approach to Folding: From Paper Craft to Protein Folding. IEEE Trans Robot Autom. 2004;20(1):60–71.

[666] Molloy K, Shehu A. Interleaving Global and Local Search for Protein Motion Computation In: Harrison R, Li Y, Mandoiu I, editors. LNCS: Bioinformatics Research and Applications. vol. 9096 Norfolk, VA: Springer International Publishing; 2015 p. 175–186.

[667] Chiang TH, Apaydin MS, Brutlag DL, Hsu D, Latombe JC. Using stochastic roadmap simulation to predict experimental quantities in protein folding kinetics: folding rates and phi-values. J Comp Biol. 2007;14(5):578–593. 10.1089/cmb.2007.R004 17683262

[668] Cortes J, Simeon T, de Angulo R, Guieysse D, Remaud-Simeon M, Tran V. A path planning approach for computing large-amplitude motions of flexible molecules. Bioinformatics. 2005;21(S1):116–125. 15961448 10.1093/bioinformatics/bti1017

[669] Shehu A. An Ab-initio tree-based exploration to enhance sampling of low-energy protein conformations. In: Trinkle J, Matsuoka Y, A CJ, editors. Robotics: Science and Systems V. Seattle, WA, USA; 2009. p. 241–248.

[670] Olson B, Molloy K, Shehu A. In Search of the Protein Native State with a Probabilistic Sampling Approach. J Bioinf & Comp Biol. 2011;9(3):383–398. 10.1142/s0219720011005574 21714131

[671] Behzadi M, Roonasi P, Assle taghipoura K, van der Spoel D, Manzetti S. Relationship between electronic properties and drug activity of seven quinoxaline compounds: A DFT study. J Phys Chem. 2015;1091(5):196–202.

[672] Khaliullin RZ, VandeVondele J, Hutter J. Efficient Linear-Scaling Density Functional Theory for Molecular Systems. J Chem Theory Comput. 2013;9(10):4421–4427. 10.1021/ct400595k 26589159

[673] Senn HM, Thiel W. QM/MM methods for biomolecular systems. Angew Chem Int Ed Engl. 2009;48(7):1198–1229. 10.1002/anie.200802019 19173328

[674] Larsson DSD, Liljas L, van der Spoel D. Virus capsid dissolution studied by microsecond molecular dynamics simulations. PLoS Comput Biol. 2012;8(5):e1002502 10.1371/journal.pcbi.1002502 22589708 PMC3349721

[675] Roy A, Zhang Y. Protein Structure Prediction In: Encyclopeda of Life Sciences. John Wiley & Sons, Ltd; 2012 p. a0003031.

[676] Scheres SHW. A Bayesian View on Cryo-EM Structure Determination. J Mol Biol. 2012;415(2):406–418. 10.1016/j.jmb.2011.11.010 22100448 PMC3314964

[677] Topf M, Lasker K, Webb B, Wolfson H, Chiu W, Sali A. Protein Structure Fitting and Refinement Guided by cryoEM Density. Structure. 2008;16(2):295–307. 10.1016/j.str.2007.11.016 18275820 PMC2409374

[678] Engel A, Gaub HE. Structure and Mechanics of Membrane Proteins. Annu Rev Biochem. 2008;77:127–148. 10.1146/annurev.biochem.77.062706.154450 18518819

