# Supplementary material for: Principles and Overview of Sampling Methods for Modeling Macromolecular Structure and Dynamics
Source: PLoS Comput Biol. 2016 Apr 28;12(4):e1004619. doi: 10.1371/journal.pcbi.1004619 (PMC4849799; doi:10.1371/journal.pcbi.1004619)
Supplement: S1 Text — Abbreviations are provided for names of methods and proteins. (PDF) [file pcbi.1004619.s001.pdf]

Supporting Information

S1 Text

Abbreviations in alphabetical order

AdK, **Ad**enylate **K**inase;
ANM, **A**nisotropic **N**etwork **M**odel;
CADD, **C**omputer-**A**ssisted **D**rug **D**iscovery;
CASP, **C**ritical **A**ssessment of protein **S**tructure **P**rediction;
cryo-EM, cryo-**E**lectron **M**icroscopy;
DIMS, **D**ynamic **Im**portance **S**ampling;
EA, **E**volutionary **A**lgorithm;
ENM, **E**lastic **N**etwork **M**odel;
EST, **E**xpansive **S**paces **T**ree;
GA, **G**enetic **A**lgorithm;
IDP, **I**ntrinsically-**D**isordered **P**rotein;
IMP, **I**ntegrative **M**odeling **P**latform;
MA, **M**emetic Evolutionary **A**lgorithm;
MC, **M**onte **C**arlo MC-SA, **M**onte **C**arlo-**S**imulated **A**nnealing;
MD, **M**olecular **D**ynamics;
MEP, **M**inimum **E**nergy **P**ath
MFEP, **M**inimum **F**ree **E**nergy **P**ath
MGA, **M**emetic **G**enetic **A**lgorithm;
MM, **M**olecular **M**echanics;
MO-EA, **M**ulti**O**bjective EA;
MO-GA, **M**ulti**O**bjective GA
MSM, **M**arkov **S**tate **M**odel;
MWC, **M**onod-**W**yman-**C**hangeaux;
NEB, **N**udged **E**lastic **B**and;
NMA, **N**ormal **M**ode **A**nalysis
NMR, **N**uclear **M**agnetic **R**esonance;
NOE, **N**uclear **O**verhauser **E**ffect;
PDB, **P**rotein **D**ata **B**ank;
PDST, **P**ath-directed **S**ubdivision **T**ree;
PNM, **P**lastic **N**etwork **M**odel;
PRM, **P**robabilistic **R**oad **M**ap;
QM, **Q**uantum **M**echanics;
RDC, **R**esidual **D**ipolar **C**oupling;
RMSD, **R**oot-**M**ean-**S**quared **D**eviation;
RRT, **R**apidly-exploring **R**andom **T**ree;
TPS, **T**ransition **P**ath **S**ampling;
TST, **T**ransition **S** **T**heory;
VMD, **V**isual **M**olecular **D**ynamics;
WEM, **W**eighted **E**nsemble **M**ethod;
